# Dupilumab treatment has no effect on the nasal microbiome in patients with NSAID-exacerbated respiratory disease: a longitudinal pilot study

**DOI:** 10.3389/fimmu.2025.1508500

**Published:** 2025-05-28

**Authors:** Tina Bartosik, Petra Pjevac, Joana Séneca, Christina Morgenstern, Tamara Arnoldner, Katharina Gangl, Christoph Sinz, Nicholas James Campion, Aldine Tu, Victoria Stanek, Christine Bangert, Sven Schneider, Julia Eckl-Dorna

**Affiliations:** ^1^ Department of Otorhinolaryngology, General Hospital and Medical University of Vienna, Vienna, Austria; ^2^ Department of Microbiology and Ecosystem Science, Centre for Microbiology and Environmental Systems Science, University of Vienna, Vienna, Austria; ^3^ Joint Microbiome Facility of the Medical University of Vienna and the University of, Vienna, Austria; ^4^ Department of Dermatology, General Hospital and Medical University of Vienna, Vienna, Austria

**Keywords:** *Staphylococcus aureus*, CRSwNP, N-ERD, dupilumab, microbiome

## Abstract

**Background:**

Non-steroidal anti-inflammatory drugs-exacerbated respiratory disease (N-ERD) affects up to 10% of patients suffering from nasal polyps and has a severe impact on quality of life. Dupilumab, a monoclonal antibody targeting the IL-4 receptor α chain, leads to symptom relief and reduction in nasal type 2 mediator levels. Here, we investigated the impact of dupilumab treatment on the composition and diversity of the nasal microbiome.

**Methodology:**

Nasal microbiome was analyzed by 16s rRNA gene amplicon sequencing in 28 patients before, 4, 12, and 24 weeks after dupilumab therapy.

**Results:**

After stringent decontamination and removal of patients whose samples contained less than 500 reads at ≥ one of the four visits, full datasets from 8 out of 28 patients remained for downstream analysis of microbiome data. All 8 patients showed significant reduction in TPS (total polyp score; p=0.0078) and an improvement in SNOT-22 (Sino-nasal outcome test-22, a quality of life questionnaire; p=0.0781) after dupilumab therapy. During 24 weeks of dupilumab therapy, there were no major changes in microbiome diversity or composition observed (Shannon index: V1-V4:p-adj=0.25, Chao 1 Index V1-V4:p-adj=0.31), and only 2 out of 8 patients showed a decrease in staphylococci abundance.

**Conclusions:**

More than 70% of the samples did not pass quality control, this warrants further refinement of nasal microbiome sampling techniques and mandatory guidelines on stringent quality control for analysis of this low biomass data in future. Furthermore, dupilumab did not have an impact on microbiome diversity or composition.

## 1 Introduction

Non-steroidal anti-inflammatory drug (NSAID)-exacerbated respiratory disease (N-ERD) affects 8-10% of patients suffering from chronic rhinosinusitis with nasal polyps (CRSwNP) (1). In addition to nasal polyps, N-ERD patients suffer from asthma and hypersensitivity to NSAIDs. The disease burden is evidenced by a high incidence of polyp recurrence and severely impaired quality of life (2, 3). In the US and Europe, patients with nasal polyps show predominantly a type 2 inflammation profile, marked by high local T helper 2 cytokine levels and a strong eosinophilic influx in the nasal polyp tissue (4). This pattern is even more pronounced in N-ERD patients, where additionally increased levels of nasal IL-5 and eosinophilic cationic protein (ECP) are observed as compared to aspirin-tolerant CRSwNP patients (5, 6). A recent study employing global Transcriptomics in combination with genome-wide methylomics and targeted metabolomics has demonstrated that these cells display an overall reduction in DNA methylation, combined with aberrant metabolic profiles and increased chemokine expression in N-ERD patients (7). Therefore the persistent metabolic and epigenetic reprogramming in macrophages could play an important role in the pathomechanism of N-ERD.

In the last decade, monoclonal antibodies targeting mediators of the type 2 inflammation pathway have become available and are a therapeutic add-on for patients with severe, refractory disease (8, 9). For example, omalizumab, a monoclonal antibody targeting free IgE, reduces the polyp burden (10) and induces complete aspirin tolerance in more than 60% of patients (11, 12). The IL-4 receptor α chain-blocking antibody dupilumab, which targets both the IL-4 and IL-13 receptors, also improves aspirin tolerance (13) and leads to significant clinical improvement in N-ERD patients (14, 15). Furthermore, it leads to a significant reduction of type 2 inflammation associated biomarkers such as Eotaxin-3, IgE and ECP in nasal secretions (13, 16). In tissue homogenates, ECP, eotaxins and pulmonary and activation-regulated chemokine (PARC) are also significantly reduced after dupilumab therapy (16). Thus, it is thought that dupilumab reduces type 2 inflammation, IgE production, drivers of and eosinophilic inflammation locally in nasal tissue.

CRSwNP, N-ERD and other chronic type 2 inflammatory diseases such as type 2 asthma or atopic dermatitis (AD) are associated with epithelial barrier dysfunction. This epithelial impairment can lead to colonization with opportunistic bacteria such as *Moraxella*, *Haemophilus*, or *Staphylococcus aureus (* 17), the latter being considered a major contributor to symptom burden in all above-mentioned diseases (18). In this context, recent findings have shown that treatment with dupilumab does not only lead to a significant clinical improvement in AD patients, but also to improved skin barrier function accompanied by reduced colonization with *Staphylococcus aureus* in both lesional and non-lesional skin (19–21). This reduction was shown to occur as early as 3 days after the beginning of treatment (22). However, whether and to what extent blockage of IL-4 and IL-13 may alter the respiratory microbiome in CRS or asthma patients is currently unknown (23). A recent cultivation based study assessing microbial profiles in CRSwNP patients after surgery or dupilumab indicates that *Staphylococcus aureus* prevalence remained stable under IL-4Rα blockage, while it increased in patients after surgery (24).

In this study, we longitudinally assessed the effect of 24 weeks of dupilumab treatment on the composition of the nasal microbiome in patients suffering from N-ERD. Patients with N-ERD have very high levels of type 2 inflammation (6) and the cohort used for this study has been carefully selected and well characterized (13). Furthermore, we previously showed that N-ERD patients on average display a higher abundance of *Staphyloccus aureus* than CRSwNP or CRSsNP patients (25). As *Staphylococcus aureus* is thought to play an important role in driving the disease, this group is very well suited to investigate changes in this amplicon sequence variant (ASV) under dupiluma therapy. As the nasal microbiome is a low (microbial) biomass habitat, making samples particularly susceptible to sampling, handling, and sequencing contamination (26–28), we included numerous sampling and DNA extraction controls and applied stringent contamination filtering workflows to ensure the validity of our data.

## 2 Materials and methods

### 2.1 Study population and clinical assessments

The samples of N-ERD patients used in this study are derived from a prospective open-label single-center study at the Department of Dermatology and the Department of Otorhinolaryngology at the Medical University of Vienna, Austria (EK 1044/2020). The study was registered with EudraCT (2019–004889–18) and ClinicalTrials.gov (NCT04442256), for further details on study conduct, in- and exclusion criteria as well as clinical assessments of the study and aspirin provocation schedule, please refer to Schneider et al (13). In the aforementioned study, nasal secretions (Nasosorption FX-I, Hunt Developments (UK) Limited, Midhurst, West Sussex, United Kingdom) and nasal microbiome samples (CLASSIQSwabs, Copan Diagnostics Inc. Murietta, CA, USA) were collected from the inferior nasal turbinate at baseline, 6 to 4 weeks after start of dupilumab treatment. In parallel to collecting patient samples, air control swabs were also collected for each individual patient at each timepoint. Patients underwent aspirin provocation at baseline and 24 weeks after beginning dupilumab treatment. For clinical characteristics of patients included in the final nasal microbiome analysis, please refer to **Table 1**. For clinical characteristics of all 31 patients initially sampled in the study, please refer to Schneider et al (13).

**Table 1:** Patient characteristics.

Age, years	Min-Max	33-68
	Median	49
Sex, n (%)	Male	5 (63%)
	Female	3 (37%)
Comorbidities, n (%)	Asthma	8 (100%)
	Allergy	5 (63%)
Number of previous surgeries	Min-Max	0-4
	Median	2.5
Clinical scores (scale), median	TPS combined (0-8)	3
	UPSIT score (0-40)	10
	SNOT-22	35

### 2.2 Inflammatory mediators and IgE measurements

The following inflammatory mediators were measured in nasal secretions of patients pooled from both nostrils at baseline and 24 weeks after start of dupilumab therapy using the electrochemiluminescence technology MSD multiplex U-Plex platform: Interleukin (IL)-1α, IL-1IR, IL-1RA, IL-2, IL-3, IL-4, IL-5, IL-6, IL-7, IL-8, IL-9, IL-10, IL-12/IL-23p40, IL-12p70, IL-13, IL-15, IL-16, IL-17A, IL-17E/IL-25, IL-21, IL-22, IL-27, IL-33, Tumor Necrosis Factor-alpha (TNF-α), TNF-β, Interferon-gamma (IFN-γ), Thymic Stromal Lymphopoietin (TSLP), Eotaxin, Eotaxin-3, Thymus and Activation-Regulated Chemokine (TARC=CCL17), Vascular Endothelial Growth Factor-A (VEGF-A), Granulocyte Colony-Stimulating Factor (G-CSF) and Granulocyte-Macrophage Colony-Stimulating Factor (GM-CSF). All measurements were performed according to the manufacturer´s instructions (https://www.mesoscale.com/en) and as described elsewhere (13). Concentration values that fell below or above the detection limits were imputed by using the mean of the estimated lower and upper detection limits for the specific cytokine across all batch readings (13). None of the reported cytokines had missing values for the higher detection limit. Higher and lower detection limits for cytokines displayed in the figures are listed in **Supplementary Table S1**. For IL-13, there were a total of 3 missing values at V1 and V4 as displayed in **Supplementary Table S2**. Additionally, IgE was determined in nasal secretions before and after 24 weeks of dupilumab therapy by ELISA as previously described (13).

### 2.3 DNA extraction and 16S rRNA gene sequencing

DNA was extracted from nasal swab samples, sampling controls (air control, where sterile swabs were opened and exposed to the air of the room in which nasal sample collection was performed) and sterile, unopened swab controls with the QIAamp DNA Microbiome Kit (Qiagen, Hildesheim, Germany) following manufacturer’s instructions. The V3V4 region of bacterial 16S rRNA genes was amplified and barcoded following a standardized 2-step PCR protocol (29), and amplicons libraries were sequenced on the Illumina MiSeq platform (v3 chemistry, 600 cycles) at the Joint Microbiome Facility of the Medical University of Vienna and the University of Vienna under project IDs JMF-2111–09 and JMF-2306-01. Amplicon pools were extracted from sequencing data using the default FASTQ workflow (Illumina, Basespace), and demultiplexed into individual libraries using the Python package demultiplex (Laros JFJ, https://github.com/jfjlaros/demultiplex), allowing 1 mismatch for barcodes and 2 mismatches for linkers and primers. Thereafter, amplicon sequence variants (ASVs) were inferred using the recommended workflow in the DADA2 R package v1.20 (30, 31). Forward and reverse sequencing reads were both trimmed at 230 nt allowing 4 and 6 expected errors, respectively. ASVs were classified using DADA2 against the SILVA database SSU Ref NR 99 release 138.1 (32) using a confidence threshold of 0.5.

Prior to downstream analysis, a stringent, multi-step decontamination procedure was employed. First, ASVs without classification or classified as eukaryotes, mitochondria, or chloroplasts, as well as well-known buffer contaminations were removed. We further used the *R* package decontam v. 0.20 to stringently decontaminate the dataset against a set of negative controls. Including swab controls, sampling controls, and extraction blanks (i.e. reagent blanks for the DNA extraction and PCR reagents). Decontamination with these settings resulted in the removal of all ASVs that were detected in only one out of two batches the samples were sequenced in, excluding any possible sequencing batch effect. ASVs flagged as contaminants after using a threshold of 0.99, as well as ASVs shorter than 300 bp were removed. After merging reads from left and right nostrils, all samples with >500 reads after decontamination for all 4 timepoints/patient were kept. The threshold of 500 reads was selected based on previous knowledge (25) about the low complexity of stringently decontaminated nasal microbiome samples. We additionally excluded patients 12 and 23, since at least one of their corresponding sampling controls (sterile swabs exposed to the air of the room in which sample collection was performed) had a very high yield, and patient 31 because he did not receive a second aspirin provocation. Following decontamination, all downstream analyses were performed using R v4.3.2 and Bioconductor v3.16 packages SummarizedExperiment v1.32, SingleCellExperiment v1.24, TreeSummarizedExperiment v2.8 (33), mia v1.8 (https://github.com/microbiome/mia), vegan v2.6-4 (https://CRAN.R-project.org/package=vegan), phyloseq v1.44 (34), and microbiome v1.22 (http://microbiome.github.io), microViz v0.10.8 (35). Alpha diversity (i.e., richness and diversity indexes) was calculated on rarefied data (557 read pairs/sample) using R packages vegan and mia. Differences in richness and diversity were estimated using pairwise Wilcoxon rank tests. All p-values were adjusted for the false discovery rate using the Benjamini-Hochberg method. Beta diversity (i.e. differences in bacterial community structure) was estimated by performing a PCoA using the Aitchison distance, with R package ade4 v 1.7-22 (36). The difference in per-group centroids was tested with a PERMANOVA on Aitchison distances using R packages vegan and microviz. Differential ASV abundance testing between V1 and V4 was performed using DESeq2 level with alpha=0.05 and otherwise default parameters after adding a pseudocount of 1 to the data (37).

### 2.4 Statistical analysis of clinical parameters

Statistical analyses of the clinical data were done using GraphPad Prism 8.4.1 (GraphPad Software, Inc., La Jolla, San Diego, CA, USA). Differences between V1 and V4 were evaluated using the Wilcoxon matched-paired signed rank test. Non-parametric tests were used due to small sample size (8 patients). Correlations between centered log ratio transformed counts of all ASVs across all four timepoints and clinical variables were estimated using R package ALDEx2 (38) using the Spearman’s correlation with FDR multiple testing correction.

## 3 Results

### 3.1 Study design and baseline characteristics

**Figure 1:**
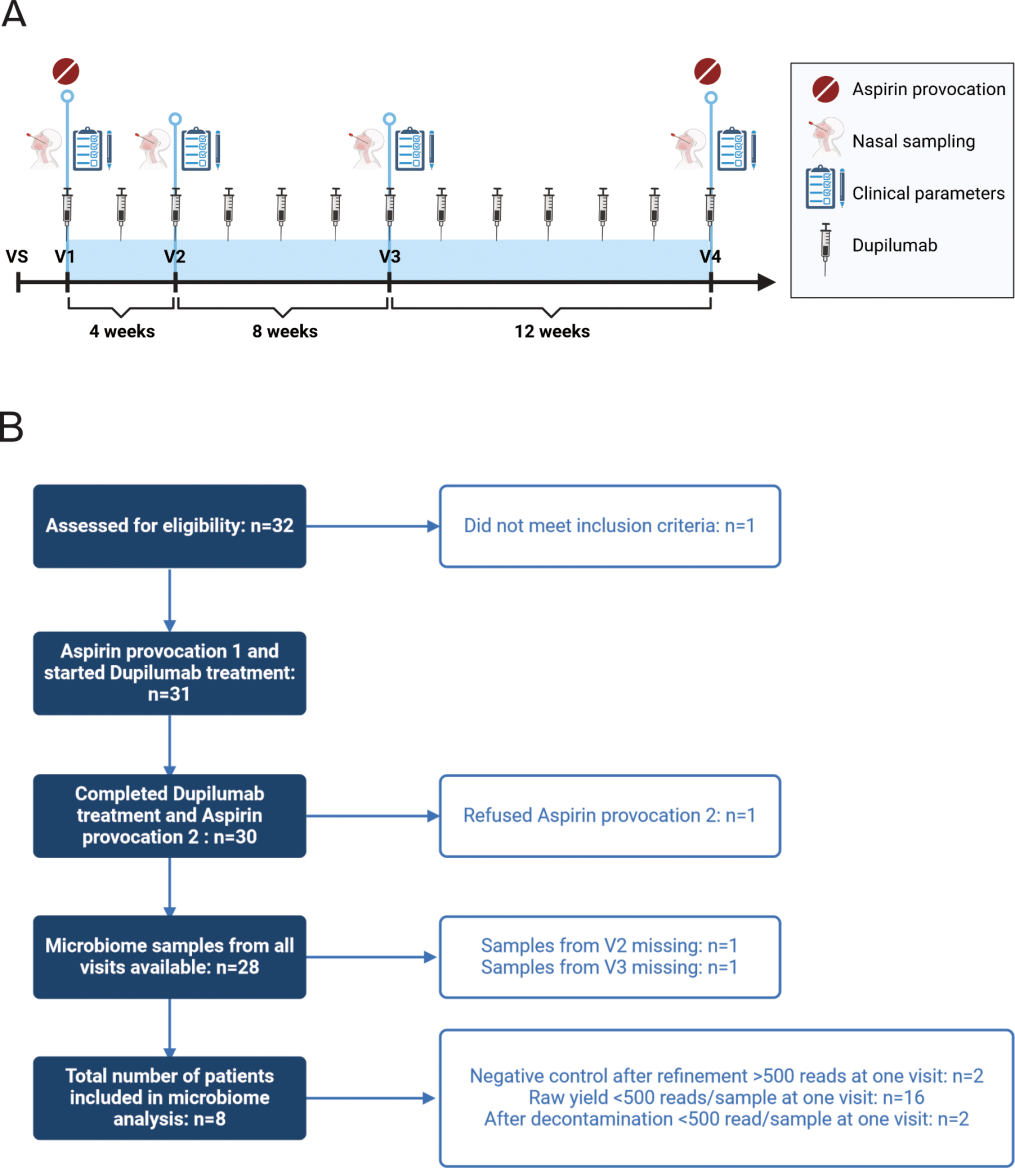
Study design. **(A)** After a screening visit (VS) to assess eligibility, patients’ nasal samples and clinical parameters were collected at the first study visit (V1) before aspirin provocation. Thereafter, dupilumab was administered in a biweekly manner, and nasal sampling and assessment of clinical parameters were performed 4 weeks (V2), 12 weeks (V3), and 24 weeks (V4) after the start of dupilumab treatment. At V4, the second aspirin provocation was performed. **(B)** Flowchart of inclusion and exclusion of samples for final microbiome analysis.

All patients in this study suffered from N-ERD and were treated with dupilumab. They received aspirin challenges at baseline before treatment start (Visit=V1) and after 24 weeks (V4) of treatment to assess tolerance development toward aspirin (**Figure 1A**). Clinical parameters and sampling of nasal secretions and nasal microbiome were performed before (V1) as well as 4 (V2), 12 (V3) and 24 (V4) weeks after start of dupilumab treatment. Microbiome samples were available from all 4 visits from 28 patients who completed the treatment including two aspirin provocations. Thus, in total 112 microbiome samples were used for 16S rRNA gene amplicon sequencing (**Figure 1B**). After stringent decontamination (including PCR negative controls, DNA extraction control, swab controls and sampling controls) and removal of patients whose samples contained less than 500 reads at least one of the four visits, full datasets from 8 patients (32 samples) remained for downstream analysis (**Supplementary Table S3**). The baseline characteristics of selected patients are displayed in **Table 1**, for characteristics of the initial cohort of patients please refer to Schneider et al (13). 5 out of 8 patients suffered from respiratory or venom allergies (**Supplementary Table S4**) and all patients continued to take their medications throughout the study (**Supplementary Table S5**). All patients took either intranasal Mometasone and/or Fluticasone.

### 3.2 Reduced total polyp score (TPS), improved quality of life, and development of aspirin tolerance during 24 weeks of dupilumab treatment

**Figure 2:**
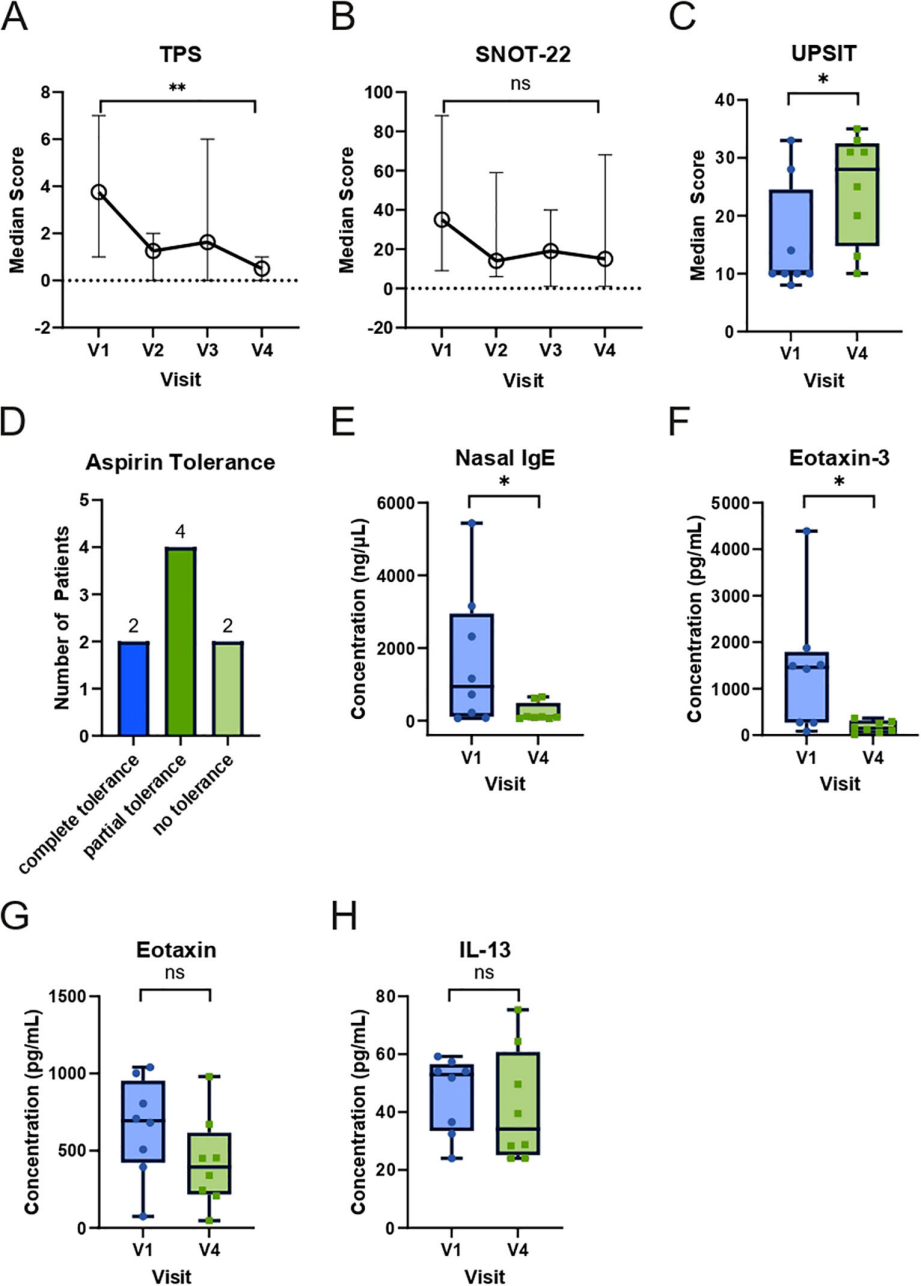
Changes in clinical and nasal mediator responses in N-ERD patients during 24 weeks of dupilumab treatment. **(A-C)** Plots display median score (y-axes) of **(A)** total polyp score (TPS, n=8), **(B)** Sino-Nasal Outcome Test-22 (SNOT-22, n=7 for V1 and V2, n=8 for V3 and V4), **(C)** University of Pennsylvania Smell Identification Test (UPSIT, n=8) at first study visit (V1) before as well as indicated visits after start of dupilumab treatment. **(D)** Number of patients (y-axis) achieving complete, partial, or no tolerance during the second aspirin provocation 24 weeks after the start of dupilumab treatment. **(E-H)** Levels of **(E)** nasal IgE, **(F)** eotaxin-3, **(G)** eotaxin, and **(H)** IL-13 in N-ERD patients (n=8) are displayed at baseline (V1) and after 24 weeks (V4) of dupilumab treatment. The line within each box represents the median, the bottom border represents the 25th percentile, and top border the 75th percentile of the data. Whiskers extend 1.5 times the interquartile range. The significance of changes between baseline and week 24 are indicated in individual graphs. *: p<0.05; **: p<0.01; ns, non-significant.

After 24 weeks of dupilumab therapy, all eight patients from whom microbiome samples were available from all visits experienced a significant reduction in TPS (p=0.0078) and an improvement in SNOT-22 (p=0.0781) and UPSIT (p=0.0156) (**Figures 2A–C**). This was accompanied by the development of partial to complete tolerance toward aspirin in 6 out of 8 patients (**Figure 2D**). In nasal secretions, a significant reduction in IgE (p=0.0234, **Figure 2E**) and eotaxin-3 (p=0.156, **Figure 2F**) was observed. Nasal eotaxin (p=0.0547, **Figure 2G**) and IL-13 (p=0.7422, **Figure 2H**) levels also dropped, but did not reach statistical significance. In addition, the other investigated cytokines did not show statistical significant changes in this timeframe (**Supplementary Figure 3**).

### 3.3 Microbial diversity and relative abundance of selected bacterial genera in the nose do not change during 24 weeks of dupilumab therapy

**Figure 3:**
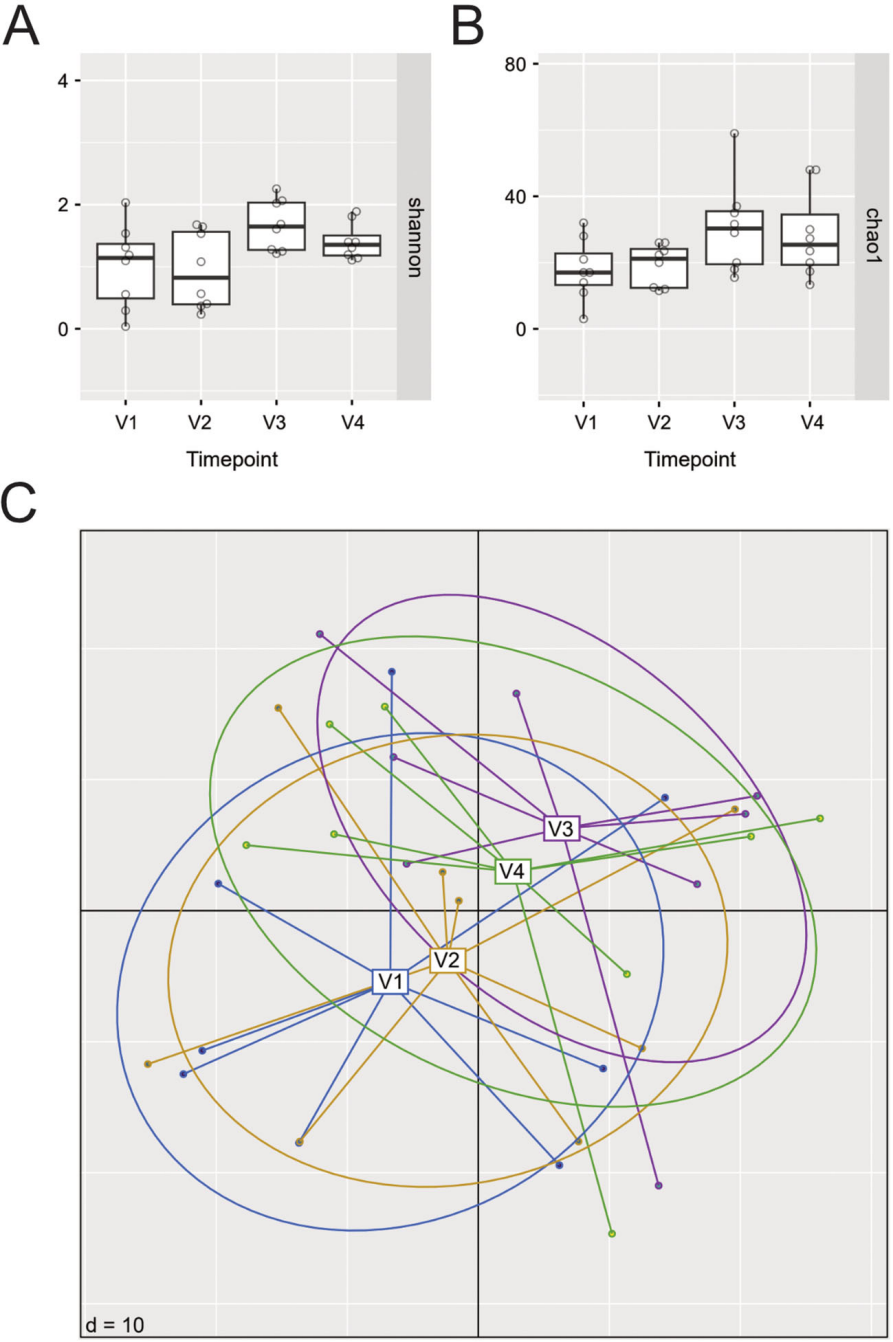
Changes in alpha and beta diversity as well as relative abundance of selected ASVs in N-ERD patients during 24 weeks of dupilumab treatment. **(A, B)** Alpha diversity before (V1) and at selected time points up to 24 weeks (V2-V4) after dupilumab treatment as shown by **(A)** Shannon and **(B)** Chao1 diversity indices. No significant differences were observed, p-values of individual comparisons are listed in **Supplementary Table 2**. **(C)** Principal Coordinates Analysis plots (PCoA) displaying beta diversity results from the 4 different visits (V1: blue, V2: yellow, V3: purple, V4: green); PERMANOVA: Timepoint, p=0.06).

We next assessed potential changes in the nasal microbiome during dupilumab therapy. To that aim, we first calculated intra-community diversity by means of the Shannon index, and species richness using the Chao1 index, and compared them across visits (**Figures 3A, B**). Both indices did not show significant changes at any of the selected time points during 24 weeks of dupilumab therapy as compared to baseline (**Figures 3A, B**) (Shannon index: V1-V4:p-adj=0.25, Chao 1 Index V1-V4:p-adj=0.31). We next calculated beta diversity to assess inter-sample diversity, community dissimilarity and potential community composition shifts during dupilumab treatment, but again no significant differences were observed between samples from the four visits (**Figure 3C**). Differential abundance testing revealed significantly increasing abundances of a few *Corynebacterium*, *Staphylococcus* (among them several *S. aureus* associated ASVs: ASV_ssr_vha (p=0.002), ASV_r8p_kle (p<0.001), ASV_mm9_js5 (p=0.002), ASV_il5_6pn (p=0.002) and ASV_906_1ja (p=0.008)), *Peptoniphilus* and *Finegoldia* after 24 weeks of dupilumab therapy as compared to baseline (**Supplementary Figure 1**). However, the majority of significantly differentially abundant ASVs were of very low relative abundance and prevalence, and given the low sample number, the observed differential abundances could be spurious. The most abundant *Staphylococcus* ASV (ASV_4pz_tc7) detected to be significantly differentially abundant between V1 and V4 (p=0.01), displayed highly variable relative abundance dynamics between patients (**Supplementary Figure 2**). Considering these constraints and the limited cohort size, these results should be interpreted with caution.

**Figure 4:**
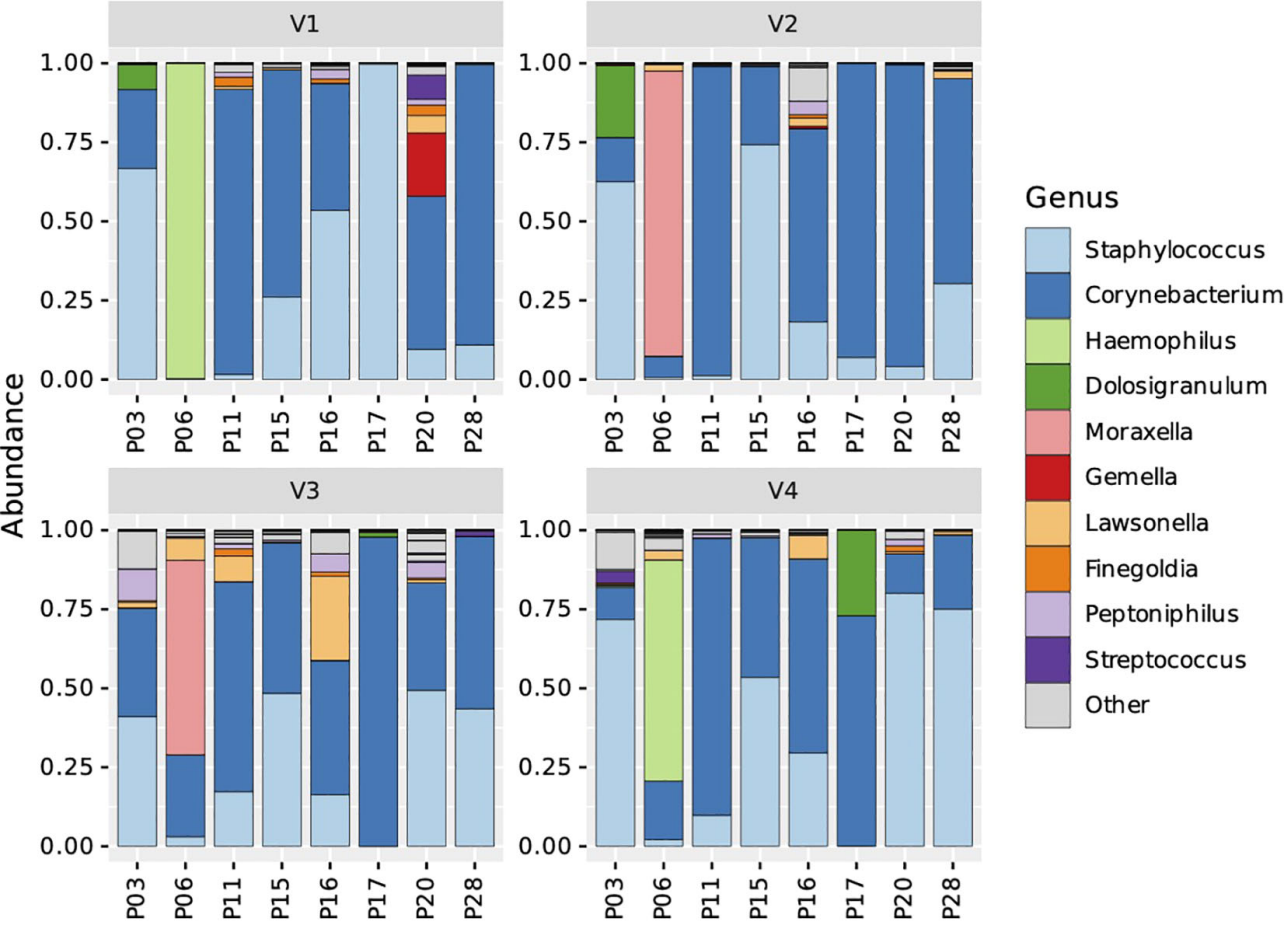
Stacked bar charts representing microbial taxonomic profiles of individual N-ERD patients (n=8) during 24 weeks of dupilumab treatment. Samples are ordered by visits (panel V1-V4) and individual patients (x-axes). The fraction of a given bar marked by specific color depicts the relative abundance of the indicated genus in the individual patient.

Finally, we analyzed the overall relative abundance of bacterial genera on an individual patient level and found that despite the general trend in increased abundance of *Staphylococcus* related ASVs in this patient cohort, a dramatic decrease was observed in 2 out of 8 patients: patient (P) 17 showed a drop from 99.8% to 0.1% and abundance in patient P16 decreased from 53.5% to 29.5% (**Figure 4**; **Supplementary Figure 4** depicts also results of patients with missing intermediate samples). The ASVs associated with genera *Haemophilus, Moraxella, Dolosigranulum* and *Gemella* were found only in single patients. Furthermore, the prevalence of change of ASV relative abundance in individual patients was also not associated with changes in TPS, SNOT-22, UPSIT score, IL-5 or IL-13 (**Supplementary Table S2**).

## 4 Discussion

Here we report on the nasal microbiome composition and longitudinal changes therein in an initial cohort of 31 N-ERD patients undergoing dupilumab treatment for 24 weeks. After stringent decontamination and filtering, samples from 8 patients with full microbiome datasets were included for downstream analysis. We found significant clinical and nasal biomarkers improvement, although no major consistent changes in microbiome composition and diversity during dupilumab therapy were observed. Selected, predominantly relatively low abundant ASVs, showed significant changes in pairwise abundance testing. While patients showed a general trend toward increased abundance of *Staphylococcus* ASVs, two patients displayed a severe drop in *Staphylococcus* abundance independently development of aspirin tolerance after 24 weeks of therapy.

Out of the samples from 28 patients subjected to 16S rRNA gene amplicon sequencing, full datasets of only 8 patients (29%) remained for the final analysis. This considerable loss in data is due to the known issue of very low initial microbial biomass of nasal samples (27). In another study involving nasopharyngeal swabs of 82 patients in the context of tuberculosis, a recovery rate of only 53% of samples using comparable filtering criteria was achieved (39). Including all samples in the downstream analysis, regardless of read counts and presence or absence of air and reagent control contaminations, bears the substantial risk of non-proportional target amplification of contaminant DNA deriving from environmental or reagent sources, which can severely impact the outcome (40). In this context, Salter et al. demonstrated in a longitudinal study of children that the differences in nasal microbial composition were not - as initially thought - age-dependent, but could be attributed solely to the usage of four extraction kits bearing different contaminations during the study (41). Thus, to avoid potential sampling or experimental bias, we employed in the present study stringent criteria as proposed in the “RIDE” checklist (26, 42) thereby accepting the exclusion of more than 70% of the samples. This high loss shows that there is an urgent need to test and develop alternative methods for nasal sampling, ideally yielding higher biomass (43), and to implement mandatory guidelines for stringent quality control during sampling allowing for comparison between published results (27). Methodologic studies in saliva microbiome, have shown that stimulated (i.e. paraffin-stimulation) and unstimulated saliva as well as simple mouth wash show comparable results with regards to microbiome diversity (44, 45). Similar studies are warranted in the field of nasal sampling comparing i.e. brushes, scrapes or nasal washes with currently used swabs using appropriate controls.

The impact of CRS on the overall diversity of the nasal microbiome has yet not fully been elucidated: While some studies observed a reduced microbial diversity in CRS-affected patients (46–49), we and others could not detect any statistically significant difference in well-established diversity indices (25, 50, 51). However, there is clear evidence for the role of *Staphylococcus aureus* and corynebacteria in the pathogenesis of CRS and their abundance is associated with type 2 mediator levels (25, 52–54). Due to the tight interaction between the microbiome, epithelium, and type 2 cytokines, it is conceivable that dupilumab may also have an influence on barrier function and, as a consequence, on microbial composition. In AD, a significant reduction of *Staphylococcus aureus* colonization of the skin was demonstrated during dupilumab treatment regardless of whether samples were taken from healthy skin or lesions (19–22, 55). Interestingly, this reduction was observed as early as 3 days after the start of treatment and preceded clinical improvements or changes in serum CCL17, a marker of type 2 disease, by 11 days (22). These results suggest an important role of the microbiome in shaping the epithelial barrier. However, data regarding the effect of dupilumab on nasal microbiome are scarce: to date only one study performed in AD patients also collected swabs from the anterior nares before and 16 weeks after dupilumab therapy (20). They initially observed a significant increase of *S. hominis* in patients achieving significant disease improvement, but subgroup analysis showed that this result was mainly driven by a few patients. This is in accordance with our observation that there is a strong inter-individual difference between patients with regards to relative abundance changes of *Staphylococcus* associated ASVs during dupilumab therapy.

In contrast to the reported decrease in *Staphylococcus aureus* colonization of skin in AD patients during dupilumab therapy, we observed no consistent significant changes in the relative abundance of staphylococci in this cohort. This is in line, a recent study showing that dupilumab as opposed to surgery stabilizes *Staphylococcus aureus* prevalence using a cultivation-based approach (24). However, our longitudinal study design does show that large, individuum-specific fluctuations in the relative abundance of *Staphylococcus-related* ASVs occur during dupilumab therapy. One explanation for the observed discrepancy between observations made in nasal and oral samples may be the differential microbial composition between nose and skin: In the nasal cavity, staphylococci usually shows (depending on the underlying inflammatory disease) a lower abundance of 2-30% within the local microbiome (25, 56, 57) as compared to AD, where staphylococci make up 75% or more of the skin microbiome (22, 58). Thus, as relative *Staphylococcus aureus* abundance drops in the skin are on average around 10% in non-lesional skin during dupilumab therapy (21, 22), such relatively small changes may not be detectable even if present in our cohort due to the small sample size, large intra-individual variability and initially low *S. aureus* abundance in many patients. Based on the results reported here, including the lack of any association of changes in *Staphylococcus* relative abundance with clinical parameters such as the development of aspirin tolerance, we propose that the link between *S. aureus* colonization and disease burden might not be as strong or omnipresent in type 2 diseases affecting respiratory epithelia as it is for those affecting skin, like AD.

The quest for biomarkers for evaluation of treatment success of biological treatment is ongoing. Here, we observed a significant reduction of type 2-associated biomarkers IgE and eotaxin during therapy. In this line, it would have been interesting to assess also non-typical biomarkers such as IL-24, which is tightly associated with IL-4 stimulation (59). Interestingly the effect of IL-4 can be directly counteracted by IFN-γ stimulation and this could be exploited as a novel treatment option for severe CRS. In this line, exogenous IFN-gamma application has been shown to lead to a better symptom control in a small scale study in ten treatment-resistant CRS patients with dysregulated IFN-γ production (60).

Limitations of the current study include the small initial participant number due to the design as a pilot trial and that the initial samples size calculation was based on the clinical primary endpoint of maximally tolerated aspirin levels (13). To calculate the number of patients required for detecting significant changes in the total microbial community in future larger trials, we conducted a power calculation based on Mattiello et al (61). The distribution from the included “anterior nares” dataset was applied; the estimated number of taxa was set to 50 (based on assuming a maximally double as high diversity as observed in our previous study (25)); and 5% of the most abundant taxa were assumed to be either 50% more or 50% less abundant each. At a sample size of n=90 and n=30 for case vs control this resulted in a power of 0.87, 0.95, and 0.51 for the significant detection of changes in the total microbial community, in the abundance of at least one taxon, and in the abundance of all taxa, respectively. However, the sample size of 90 patients with N-ERD and treated with dupilumab is beyond the scope of a pilot trial. This was further reduced by stringent quality control. A confounding factor might be the continuation of standard CRS therapy during the clinical trial which includes topical corticosteroid therapy. But due to the severe burden in N-ERD patients, mainstay therapy had to be continued following current guidelines (9). As in the current sub study we included clinical data of only 8 patients of the initial trial involving 31 subjects, the higher tolerance development toward aspirin observed as compared to our previous observation (13) is most likely due to the small sample size. Another limitation is that patients also received standard nasal corticosteroids for the duration of the study. This may have partly confounded our findings however due to the severe symptom burden of N-ERD patients it was not possible to stop nasal corticosteroids for the duration of the trial. Importantly, none of the patients received oral steroids or antibiotics for the duration of the study.

In summary, we showed in this pilot trial, that dupilumab does not affect the overall diversity of the nasal microbiome, but may lead to alterations in microbiome composition, including changes in *Staphylococcus* relative abundance in selected individuals. The fact that more than 70% of the samples did not yield enough biomass to be processed warrants further refinement of nasal microbiome sampling techniques and mandatory guidelines on stringent quality control for analysis of this low biomass data.

## Data Availability

The datasets presented in this study can be found in online repositories. The names of the repository/repositories and accession number(s) can be found below: https://www.ncbi.nlm.nih.gov/, PRJNA1090288.
