# Supplementary material for: Dupilumab treatment has no effect on the nasal microbiome in patients with NSAID-exacerbated respiratory disease: a longitudinal pilot study
Source: Front Immunol. 2025 May 28;16:1508500. doi: 10.3389/fimmu.2025.1508500 (PMC12151831; doi:10.3389/fimmu.2025.1508500)
Supplement: Supplementary file 1 [file DataSheet1.pdf]

Supplementary Material

1 Supplementary Figures and Tables

Table S1. Calculated lower and higher detection limits for MSD cytokine assays.

**Assay**	**Lower detection limit [pg/mL]**	**Higher detection limit [pg/mL]**
**Eotaxin**	93.842938	31275.000000
**Eotaxin-3**	30.753878	99500.000000
**IL-13**	24.051413	10350.000000

Table S2. Missing data in MSD cytokine assays (n=8 patients).

	**n missing**		**% missing**	
**Assay**	**V1**	**V4**	**V1**	**V4**
**Eotaxin**	0	0	0	0
**Eotaxin-3**	0	0	0	0
**IL-13**	1	2	14.29	28.57

Table S3: Baseline data of patients and reason for exclusion (grey) of microbiome samples from further analysis. SNOT-22: sino-nasal outcome test-22; TPS: total polyp score; M: male; F: female

**Patient Number**	**age**	**sex**	**SNOT-22**	**TPS**	**Reason for exclusion of patient**
001	54	M	24	2	Raw reads <500 reads/sample at V4
002	43	F	61	3	Raw reads <500 reads/sample at V3
003	47	F	34	1	Included
004	31	F	40	6	Raw reads <500 reads/sample at V1 and V3
005	49	F	17	2	After decontamination <500 reads/sample at V4
006	33	F	83	3	Included
007	48	F	8	3	Raw reads <500 reads/sample at V1, V2 and V3
008	50	M	64	0	After decontamination <500 reads/sample at V4
009	27	F	28	0	Swab V3 missing and raw reads <500 reads/sample at V1 and V2
010	70	F	20	0	Raw reads <500 reads/sample at V4
011	36	M	28	3	Included
012	43	M	75	7	V3 negative control after refinement had > 500 reads
013	38	M	40	7	Raw reads <500 reads/sample at V1 and V4
014	67	F	5	0	Raw reads <500 reads/sample at V1, V2 and V3
015	68	M	8	3	Included
016	33	F	NA	3	Included
017	65	M	56	4	Included
018	40	M	74	8	Swab V2 missing and raw reads <500 reads/sample at V4
019	36	M	65	6	Raw reads <500 reads/sample at V3
020	51	M	80	7	Included
021	47	F	27	3	Raw reads <500 reads/sample at V3
022	56	M	62	4	Raw reads <500 read/sample at V3
023	63	M	41	5	V1 negative control after refinement > 500 reads
024	33	M	26	1	Raw reads <500 reads/sample at V1, V2 and V3
025	35	M	39	7	Raw reads <500 reads/sample at V1 and V3
026	42	F	23	2	Raw reads <500 reads/sample at V1, V2 and V4
027	51	M	13	1	Raw reads <500 reads/sample at V1
028	56	M	28	6	Included
029	47	F	56	5	Raw reads <500 reads/sample at V1, V2 and V3
030	45	F	71	3	Raw reads <500 reads/sample at V2, V3 and V4
031	40	M	40	6	No second aspirin provocation performed

Table S4. Demographics and allergies of included patients (n=8 patients). Sex: M: Male, F: Female; Allergy: A=Animal dander, F=Food, G= Grass, HDM= House dust mite; M=Mould, T=Tree, V=Venom

Patient number	Sex	Age	Allergy according to skin prick test/ImmunoCAP
3	F	47	none
6	F	33	A
11	M	36	V
15	M	68	none
16	F	33	G, HDM, T
17	M	65	none
20	M	851	A, G, HDM, T
28	M	56	T,V

Table S5. Medication of included patients (n=8 patients). Pat nr = Patient number, Y = Yes, SOS = if necessary

	**nose**			**lungs**								**allergy**				**others**
**Pat nr**	Mometason	Fluticason/Azelastin	Budesonid	Tiotropium/Olodaterol	Formoterol/Budesonid	Fenoterol/Ipratropium	Fluticason/Salmeterol	Ciclesonid	Beclometason/Formoterol	Salbutamol	Montelukast	Levocetirizin	Cetirizin	Desloratadin	Prednisolon	
3		Y					SOS									
6	Y								Y		SOS			Y		Lisinopril
11	Y															
15							Y						Y			Levothyrexin, Metformin, Carvedilol, Enalapril, Enalapril/Hydrochlorothiazid, Rilmenidin
16	Y	SOS										Y				
17	Y		Y			SOS	Y	Y		SOS					Y	Candesartan, Amlodipin, Calcium/Colecalciferol, Theophyllin
20	Y			Y	Y	SOS										
28	Y						Y									

Table S6: Correlations between centered log ratio transformed counts of all ASVs across all four timepoints and clinical variables (Total polyp score=TPS, Sinonasal-outcome test-22=SNOT-22, University of Pennsylvania Smell Test score=UPSIT score) as well as cytokines (interleukin 5=IL-5, IL-13). Spearman.ep:p-value of Spearman test, Spearman.eBH: p-value after correction.

Variable	FeatureID	spearman.erho	spearman.ep	spearman.eBH	Domain	Phylum	Class	Order	Family	Genus	Species
TPS	ASV_17p_uuy	-0,090074821	0,595300184	0,959434011	Bacteria	Actinobacteriota	Actinobacteria	Corynebacteriales	Corynebacteriaceae	Corynebacterium	NA
TPS	ASV_19p_rjx	-0,06152588	0,55476985	0,925252027	Bacteria	Bacteroidota	Bacteroidia	Bacteroidales	Prevotellaceae	Prevotella_9	NA
TPS	ASV_1af_96k	-0,032668609	0,484268369	0,910969966	Bacteria	Firmicutes	Bacilli	Lactobacillales	Carnobacteriaceae	Dolosigranulum	NA
TPS	ASV_1dc_ew y	-0,184206407	0,343553198	0,857789722	Bacteria	Firmicutes	Bacilli	Lactobacillales	Carnobacteriaceae	Dolosigranulum	NA
TPS	ASV_1kn_zel	0,099242243	0,525123829	0,935829478	Bacteria	Actinobacteriota	Actinobacteria	Corynebacteriales	Corynebacteriaceae	Lawsonella	NA
TPS	ASV_1ob_3tg	-0,031618841	0,567953746	0,93309127	Bacteria	Firmicutes	Bacilli	Staphylococcales	Staphylococcaceae	Staphylococcus	NA
TPS	ASV_1wh_81 x	0,172728052	0,36596138	0,869104076	Bacteria	Firmicutes	Negativicutes	Veillonellales-Selenomonadales	Veillonellaceae	Negativicoccus	succinicivorans
TPS	ASV_20k_5qe	-0,008098061	0,494489422	0,91953375	Bacteria	Actinobacteriota	Actinobacteria	Corynebacteriales	Nocardiaceae	Millisia	NA
TPS	ASV_242_lzz	0,073926134	0,671440921	0,966052381	Bacteria	Actinobacteriota	Actinobacteria	Corynebacteriales	Corynebacteriaceae	Corynebacterium	aurimucosum/pseudogenitalium/tuberculostearicum
TPS	ASV_2d3_euj	0,053396883	0,6513549	0,959760034	Bacteria	Actinobacteriota	Actinobacteria	Corynebacteriales	Corynebacteriaceae	Corynebacterium	NA
TPS	ASV_2db_0u m	-0,063186122	0,50572694	0,929733569	Bacteria	Firmicutes	Negativicutes	Veillonellales-Selenomonadales	Veillonellaceae	Dialister	NA
TPS	ASV_2ee_ogi	0,023963167	0,600425743	0,952134057	Bacteria	Firmicutes	Bacilli	Staphylococcales	Staphylococcaceae	Staphylococcus	lugdunensis
TPS	ASV_2iq_1yo	0,039296171	0,49059209	0,926074775	Bacteria	Firmicutes	Clostridia	Peptococcales	Peptococcaceae	Peptococcus	NA
TPS	ASV_2o1_p31	-0,036758372	0,534468082	0,92505374	Bacteria	Firmicutes	Clostridia	Lachnospirales	Lachnospiraceae	Lachnospiraceae AC2044 group	bacterium
TPS	ASV_2x6_j18	0,067992574	0,506585036	0,921208499	Bacteria	Firmicutes	Clostridia	Peptostreptococcales-Tissierellales	Peptostreptococcace ae	Criibacterium	NA
TPS	ASV_2xa_4rw	0,043095134	0,509824834	0,927614595	Bacteria	Bacteroidota	Bacteroidia	Bacteroidales	Prevotellaceae	Prevotella_9	NA
TPS	ASV_2xm_w9 6	-0,055520136	0,519187166	0,931227061	Bacteria	Firmicutes	Clostridia	Lachnospirales	Lachnospiraceae	Dorea	formicigenerans
TPS	ASV_30c_6sw	0,077943713	0,498052755	0,925285057	Bacteria	Proteobacteria	Gammaproteobacteria	Burkholderiales	Oxalobacteraceae	Massilia	timonae
TPS	ASV_339_mv y	0,027563108	0,529741869	0,929346884	Bacteria	Proteobacteria	Gammaproteobacteria	Enterobacterales	Pasteurellaceae	Haemophilus	NA
TPS	ASV_3em_n0 d	-0,035513707	0,557874341	0,939693236	Bacteria	Firmicutes	Bacilli	Lactobacillales	Lactobacillaceae	Liquorilactobacillus	NA
TPS	ASV_3en_7o w	-0,090164536	0,510747328	0,930494593	Bacteria	Firmicutes	Bacilli	Staphylococcales	Gemellaceae	Gemella	morbillorum
TPS	ASV_3i5_y1w	0,041563793	0,529699702	0,940849479	Bacteria	Proteobacteria	Gammaproteobacteria	Enterobacterales	Pasteurellaceae	Haemophilus	NA
TPS	ASV_3in_1yl	-0,021193348	0,537809741	0,932309356	Bacteria	Firmicutes	Bacilli	Staphylococcales	Staphylococcaceae	NA	NA
TPS	ASV_3kg_4bk	-0,229639285	0,25397846	0,833570976	Bacteria	Firmicutes	Bacilli	Lactobacillales	Carnobacteriaceae	Dolosigranulum	pigrum
TPS	ASV_3kh_nw 9	0,020308573	0,619930547	0,956329537	Bacteria	Actinobacteriota	Actinobacteria	Corynebacteriales	Corynebacteriaceae	Corynebacterium	NA
TPS	ASV_3pn_tj1	-0,100811739	0,469473871	0,906181054	Bacteria	Actinobacteriota	Actinobacteria	Corynebacteriales	Corynebacteriaceae	Corynebacterium	NA
TPS	ASV_3po_bb2	-0,022447294	0,494404233	0,919371761	Bacteria	Actinobacteriota	Actinobacteria	Corynebacteriales	Corynebacteriaceae	Corynebacterium	NA
TPS	ASV_3tk_6ez	-0,05953359	0,49205058	0,923994015	Bacteria	Actinobacteriota	Actinobacteria	Corynebacteriales	Corynebacteriaceae	Corynebacterium	NA
TPS	ASV_3tp_m3o	0,036591317	0,514160626	0,918128964	Bacteria	Firmicutes	Bacilli	Staphylococcales	Staphylococcaceae	Staphylococcus	NA

Variable	FeatureID	spearman.erho	spearman.ep	spearman.eBH	Domain	Phylum	Class	Order	Family	Genus	Species
TPS	ASV_3w7_jlb	0,012545653	0,489542274	0,919704313	Bacteria	Firmicutes	Bacilli	Staphylococcales	Staphylococcaceae	NA	NA
TPS	ASV_440_o98	0,218881743	0,297650361	0,819481418	Bacteria	Firmicutes	Clostridia	Peptostreptococcales-Tissierellales	Family XI	Anaerococcus	NA
TPS	ASV_46z_g65	-0,073823013	0,451447927	0,915576613	Bacteria	Firmicutes	Negativicutes	Veillonellales-Selenomonadales	Veillonellaceae	Negativicoccus	NA
TPS	ASV_47r_9ra	0,007983598	0,54529055	0,936560406	Bacteria	Actinobacteriota	Actinobacteria	Corynebacteriales	Corynebacteriaceae	Corynebacterium	NA
TPS	ASV_49k_gjb	-0,041558637	0,56159924	0,939730081	Bacteria	Firmicutes	Clostridia	Oscillospirales	Oscillospiraceae	UCG-002	NA
TPS	ASV_4b4_zwy	0,048235696	0,496954343	0,929135741	Bacteria	Campylobacterota	Campylobacteria	Campylobacterales	Campylobacteraceae	Campylobacter	ureolyticus
TPS	ASV_4cz_ka1	-0,073200165	0,539618921	0,924917628	Bacteria	Firmicutes	Bacilli	Staphylococcales	Staphylococcaceae	Staphylococcus	NA
TPS	ASV_4kw_hck	-0,071302745	0,514426954	0,930090702	Bacteria	Firmicutes	Clostridia	Peptostreptococcales-Tissierellales	Family XI	Anaerococcus	NA
TPS	ASV_4m8_kdu	-0,045564873	0,548453182	0,936951886	Bacteria	Bacteroidota	Bacteroidia	Bacteroidales	Prevotellaceae	Prevotella_9	NA
TPS	ASV_4n2_dht	-0,055627382	0,545657176	0,938836031	Bacteria	Bacteroidota	Bacteroidia	Chitinophagales	Chitinophagaceae	Chitinophaga	humicola
TPS	ASV_4ni_ro3	0,020308573	0,516739402	0,926254235	Bacteria	Actinobacteriota	Actinobacteria	Corynebacteriales	Corynebacteriaceae	Corynebacterium	NA
TPS	ASV_4nj_u2c	-0,033120277	0,508844813	0,924173121	Bacteria	Actinobacteriota	Actinobacteria	Corynebacteriales	Corynebacteriaceae	NA	NA
TPS	ASV_4pz_tc7	0,219557183	0,215626026	0,88731392	Bacteria	Firmicutes	Bacilli	Staphylococcales	Staphylococcaceae	Staphylococcus	argenteus/aureus/equorum/phage/schweitzeri/simiae
TPS	ASV_4qr_yd2	-0,029167664	0,497429815	0,932560987	Bacteria	Firmicutes	Bacilli	Lactobacillales	Streptococcaceae	Streptococcus	NA
TPS	ASV_4rd_dxc	-0,164339191	0,383912872	0,912964163	Bacteria	Actinobacteriota	Actinobacteria	Corynebacteriales	Corynebacteriaceae	Corynebacterium	NA
TPS	ASV_513_wtm	0,041440048	0,526881717	0,924329822	Bacteria	Proteobacteria	Gammaproteobacteria	Enterobacterales	Enterobacteriaceae	Raoultella	NA
TPS	ASV_53d_zfm	-0,018945318	0,863893625	0,980042821	Bacteria	Actinobacteriota	Actinobacteria	Corynebacteriales	Corynebacteriaceae	Lawsonella	NA
TPS	ASV_53v_62f	0,151493456	0,403840409	0,933114758	Bacteria	Firmicutes	Bacilli	Staphylococcales	Staphylococcaceae	Staphylococcus	NA
TPS	ASV_566_wbp	0,046717761	0,520446092	0,939923899	Bacteria	Actinobacteriota	Actinobacteria	Corynebacteriales	Corynebacteriaceae	Corynebacterium	NA
TPS	ASV_5do_e0k	0,006651279	0,538530729	0,942589539	Bacteria	Actinobacteriota	Actinobacteria	Corynebacteriales	Corynebacteriaceae	Corynebacterium	NA
TPS	ASV_5j9_202	-0,349419029	0,068051214	0,697930346	Bacteria	Actinobacteriota	Actinobacteria	Corynebacteriales	Corynebacteriaceae	Corynebacterium	NA
TPS	ASV_5mh_1v6	0,045000803	0,560788586	0,941030995	Bacteria	Firmicutes	Bacilli	Staphylococcales	Staphylococcaceae	Staphylococcus	NA
TPS	ASV_5oo_dg6	-0,138146556	0,443284618	0,9023229	Bacteria	Actinobacteriota	Actinobacteria	Micrococcales	Micrococcaceae	Rothia	mucilaginosa
TPS	ASV_5r4_ie2	-0,046613609	0,555784503	0,939327178	Bacteria	Actinobacteriota	Actinobacteria	Corynebacteriales	Corynebacteriaceae	Corynebacterium	NA
TPS	ASV_5rr_zcf	0,027655916	0,486701192	0,921927815	Bacteria	Actinobacteriota	Actinobacteria	Corynebacteriales	Corynebacteriaceae	Corynebacterium	NA
TPS	ASV_5ry_ol3	0,012139358	0,529161866	0,928527346	Bacteria	Actinobacteriota	Actinobacteria	Corynebacteriales	Corynebacteriaceae	Corynebacterium	NA
TPS	ASV_5xz_bqw	0,084774422	0,447537557	0,903787801	Bacteria	Firmicutes	Bacilli	Lactobacillales	Lactobacillaceae	Liquorilactobacillus	NA
TPS	ASV_62i_805	0,08371228	0,508325993	0,915770323	Bacteria	Proteobacteria	Gammaproteobacteria	Enterobacterales	Enterobacteriaceae	Pseudocitrobacter	NA
TPS	ASV_66n_hd0	0,291369345	0,112959707	0,789474381	Bacteria	Firmicutes	Bacilli	Staphylococcales	Staphylococcaceae	Staphylococcus	aureus
TPS	ASV_6dr_o2s	-0,03140435	0,502013815	0,915703983	Bacteria	Firmicutes	Bacilli	Staphylococcales	Staphylococcaceae	Staphylococcus	NA
TPS	ASV_6ut_tpw	0,037458561	0,559783033	0,935179946	Bacteria	Proteobacteria	Gammaproteobacteria	Burkholderiales	Comamonadaceae	Tepidimonas	NA
TPS	ASV_6zj_cil	0,044422296	0,559718494	0,946327186	Bacteria	Proteobacteria	Gammaproteobacteria	Pseudomonadales	Moraxellaceae	Moraxella	nonliquefaciens

Variable	FeatureID	spearman.erho	spearman.ep	spearman.eBH	Domain	Phylum	Class	Order	Family	Genus	Species
TPS	ASV_727_4zo	-0,25398503	0,226006325	0,790187636	Bacteria	Firmicutes	Bacilli	Lactobacillales	Carnobacteriaceae	Dolosigranulum	NA
TPS	ASV_79g_5uy	-0,058395139	0,513404929	0,923413626	Bacteria	Firmicutes	Clostridia	Lachnospirales	Lachnospiraceae	Roseburia	NA
TPS	ASV_7cr_wom	0,133085396	0,460922944	0,91727728	Bacteria	Firmicutes	Bacilli	Staphylococcales	Staphylococcaceae	Staphylococcus	NA
TPS	ASV_7jt_wq3	-0,150230229	0,421645531	0,901111734	Bacteria	Actinobacteriota	Actinobacteria	Corynebacteriales	Corynebacteriaceae	Corynebacterium	NA
TPS	ASV_7ka_s2c	-0,053179298	0,524664844	0,928302362	Bacteria	Actinobacteriota	Actinobacteria	Corynebacteriales	Corynebacteriaceae	Corynebacterium	NA
TPS	ASV_7sw_rnc	-0,049206061	0,477314291	0,909004604	Bacteria	Proteobacteria	Gammaproteobacteria	Enterobacterales	Pasteurellaceae	Haemophilus	NA
TPS	ASV_7x9_tvb	0,093601546	0,590123545	0,959883225	Bacteria	Firmicutes	Clostridia	Peptostreptococcales-Tissierellales	Family XI	Peptoniphilus	gorbachii/lacydonensis/rhinitidis
TPS	ASV_873_9b5	0,085280744	0,547511314	0,936508521	Bacteria	Firmicutes	Bacilli	Staphylococcales	Staphylococcaceae	NA	NA
TPS	ASV_8fh_5b9	0,025739935	0,484535464	0,917127159	Bacteria	Proteobacteria	Gammaproteobacteria	Enterobacterales	Pasteurellaceae	Haemophilus	NA
TPS	ASV_8gj_ney	0,004050578	0,53028515	0,926988281	Bacteria	Firmicutes	Bacilli	Staphylococcales	Staphylococcaceae	Staphylococcus	NA
TPS	ASV_8lf_xek	-0,066040501	0,514044154	0,926361678	Bacteria	Bacteroidota	Bacteroidia	Bacteroidales	Prevotellaceae	Prevotella_9	copri
TPS	ASV_8lv_u9v	0,068305029	0,48442962	0,909751708	Bacteria	Firmicutes	Bacilli	Lactobacillales	Streptococcaceae	Streptococcus	NA
TPS	ASV_8pb_fin	-0,051643832	0,532957127	0,934962214	Bacteria	Bacteroidota	Bacteroidia	Cytophagales	Hymenobacteraceae	Hymenobacter	NA
TPS	ASV_8qr_nog	0,026096733	0,54189224	0,923265264	Bacteria	Actinobacteriota	Actinobacteria	Corynebacteriales	Corynebacteriaceae	Corynebacterium	NA
TPS	ASV_8re_um4	-0,364973742	0,065732361	0,628610434	Bacteria	Firmicutes	Bacilli	Staphylococcales	Staphylococcaceae	Staphylococcus	NA
TPS	ASV_8v2_sgp	-0,168636226	0,38402577	0,89662431	Bacteria	Actinobacteriota	Actinobacteria	Corynebacteriales	Corynebacteriaceae	Corynebacterium	NA
TPS	ASV_8zu_e97	0,037538995	0,58353881	0,943426429	Bacteria	Firmicutes	Bacilli	Staphylococcales	Staphylococcaceae	Staphylococcus	NA
TPS	ASV_926_7zf	0,158606716	0,399015363	0,906654173	Bacteria	Firmicutes	Bacilli	Staphylococcales	Staphylococcaceae	Staphylococcus	NA
TPS	ASV_98z_21y	0,009183921	0,509358112	0,922283938	Bacteria	Proteobacteria	Gammaproteobacteria	Pseudomonadales	Pseudomonadaceae	Pseudomonas	taetrolens
TPS	ASV_99w_90h	0,110260681	0,442344429	0,907709191	Bacteria	Proteobacteria	Gammaproteobacteria	Enterobacterales	Enterobacteriaceae	NA	NA
TPS	ASV_9cd_tui	-0,018450339	0,507518183	0,922531814	Bacteria	Firmicutes	Bacilli	Lactobacillales	Enterococcaceae	Tetragenococcus	NA
TPS	ASV_9d0_o06	0,207354922	0,314157997	0,854887111	Bacteria	Firmicutes	Negativicutes	Veillonellales-Selenomonadales	Veillonellaceae	Negativicoccus	NA
TPS	ASV_9eq_kr6	-0,067451191	0,51459146	0,914077679	Bacteria	Firmicutes	Bacilli	Lactobacillales	Streptococcaceae	Streptococcus	mitis/parasanguinis
TPS	ASV_9ho_7z1	-0,063941996	0,488184329	0,926136092	Bacteria	Actinobacteriota	Actinobacteria	Actinomycetales	Actinomycetaceae	NA	NA
TPS	ASV_9jb_2ek	0,032048854	0,631791422	0,959469873	Bacteria	Actinobacteriota	Actinobacteria	Corynebacteriales	Corynebacteriaceae	Corynebacterium	NA
TPS	ASV_9kg_f3f	-0,015809421	0,561221657	0,941451367	Bacteria	Actinobacteriota	Actinobacteria	Corynebacteriales	NA	NA	NA
TPS	ASV_9l0_247	-0,068353496	0,530129141	0,927997125	Bacteria	Firmicutes	Bacilli	Staphylococcales	Gemellaceae	Gemella	morbillorum
TPS	ASV_9mx_kho	0,106850482	0,479321402	0,920880258	Bacteria	Actinobacteriota	Actinobacteria	Frankiales	Sporichthyaceae	NA	NA
TPS	ASV_9o6_1ja	0,148004886	0,427746255	0,921712012	Bacteria	Firmicutes	Bacilli	Staphylococcales	Staphylococcaceae	Staphylococcus	aureus
TPS	ASV_9t2_1wo	0,026045172	0,503876223	0,919594796	Bacteria	Proteobacteria	Gammaproteobacteria	Pseudomonadales	Moraxellaceae	Acinetobacter	NA
TPS	ASV_9vy_jj2	-0,002723415	0,566904884	0,949129821	Bacteria	Firmicutes	Bacilli	Staphylococcales	Staphylococcaceae	Staphylococcus	NA
TPS	ASV_9wz_9u7	0,007191631	0,559645018	0,945387677	Bacteria	Actinobacteriota	Actinobacteria	NA	NA	NA	NA

Variable	FeatureID	spearman.erho	spearman.ep	spearman.eBH	Domain	Phylum	Class	Order	Family	Genus	Species
TPS	ASV_a5h_c27	0,022134839	0,535065481	0,939640477	Bacteria	Firmicutes	Bacilli	Lactobacillales	Lactobacillaceae	Lacticaseibacillus	NA
TPS	ASV_aew_fyi	0,051125136	0,522780355	0,932186118	Bacteria	Actinobacteriota	Actinobacteria	Corynebacteriales	Corynebacteriaceae	Corynebacterium	mucifaciens
TPS	ASV_ajo_fen	-0,024336464	0,658887398	0,959566272	Bacteria	Actinobacteriota	Actinobacteria	Corynebacteriales	Corynebacteriaceae	Corynebacterium	NA
TPS	ASV_aqj_vcn	-0,287223896	0,165034264	0,77397825	Bacteria	Firmicutes	Bacilli	Lactobacillales	Streptococcaceae	Streptococcus	mitis/phage/pneumoniae/pseudopneumoniae
TPS	ASV_arl_xwg	-0,058018749	0,496154459	0,913114474	Bacteria	Firmicutes	Clostridia	Lachnospirales	Lachnospiraceae	Blautia	NA
TPS	ASV_aw8_q96	-0,004629084	0,705265822	0,968346844	Bacteria	Actinobacteriota	Actinobacteria	Corynebacteriales	Corynebacteriaceae	Corynebacterium	NA
TPS	ASV_b6a_uff	-0,005245745	0,517605054	0,932001463	Bacteria	Bacteroidota	Bacteroidia	Sphingobacteriales	Sphingobacteriaceae	Sphingobacterium	NA
TPS	ASV_bg2_zbh	-0,154218934	0,401651626	0,88443765	Bacteria	Firmicutes	Bacilli	Staphylococcales	Staphylococcaceae	Staphylococcus	NA
TPS	ASV_bh3_94k	-0,037579213	0,557492029	0,931993315	Bacteria	Firmicutes	Bacilli	Lactobacillales	Streptococcaceae	Lactococcus	NA
TPS	ASV_bnk_uhh	0,350777128	0,0686084	0,671858081	Bacteria	Firmicutes	Clostridia	Peptostreptococcales-Tissierellales	Family XI	Anaerococcus	provencensis
TPS	ASV_bsd_ayc	0,039639562	0,544046684	0,940667376	Bacteria	Planctomycetota	Planctomycetes	Pirellulales	Pirellulaceae	NA	NA
TPS	ASV_btl_vxl	0,087685517	0,497726981	0,928371713	Bacteria	Firmicutes	Clostridia	Peptostreptococcales-Tissierellales	Family XI	Anaerococcus	vaginalis
TPS	ASV_bwx_wef	0,176315618	0,373973882	0,86856194	Bacteria	Actinobacteriota	Actinobacteria	Corynebacteriales	Corynebacteriaceae	Corynebacterium	amycolatum/jeikeium/lactis/urealyticum/vitaeruminis
TPS	ASV_c46_jpb	-0,137488646	0,43653481	0,906842083	Bacteria	Actinobacteriota	Actinobacteria	Corynebacteriales	Corynebacteriaceae	Corynebacterium	NA
TPS	ASV_c4o_xms	0,041772097	0,592738406	0,953120215	Bacteria	Firmicutes	Bacilli	Staphylococcales	Staphylococcaceae	NA	NA
TPS	ASV_cep_rux	0,165413707	0,375366467	0,904548737	Bacteria	Firmicutes	Clostridia	Peptostreptococcales-Tissierellales	Family XI	Anaerococcus	NA
TPS	ASV_cje_lx4	0,170741949	0,379890183	0,873490999	Bacteria	Proteobacteria	Gammaproteobacteria	Burkholderiales	Neisseriaceae	NA	NA
TPS	ASV_co0_sbi	-0,144426601	0,396982416	0,89373564	Bacteria	Actinobacteriota	Actinobacteria	Corynebacteriales	Corynebacteriaceae	Corynebacterium	NA
TPS	ASV_cqp_otd	-0,025275893	0,495711651	0,916302026	Bacteria	Firmicutes	Negativicutes	Veillonellales-Selenomonadales	Veillonellaceae	Negativicoccus	NA
TPS	ASV_cy2_emp	0,092495062	0,458374897	0,905582962	Bacteria	Actinobacteriota	Actinobacteria	Corynebacteriales	Corynebacteriaceae	Corynebacterium	NA
TPS	ASV_czd_a30	-0,01757897	0,514100851	0,929484546	Bacteria	Firmicutes	Bacilli	Staphylococcales	Staphylococcaceae	Staphylococcus	NA
TPS	ASV_d0q_oe1	-0,068297811	0,48662476	0,918971129	Bacteria	Bacteroidota	Bacteroidia	Bacteroidales	Prevotellaceae	Prevotella_9	NA
TPS	ASV_da2_u30	0,148156473	0,400169575	0,885727934	Bacteria	Bacteroidota	Bacteroidia	Bacteroidales	Prevotellaceae	Prevotella	buccalis
TPS	ASV_dhe_20n	0,06213223	0,675455045	0,962633732	Bacteria	Firmicutes	Bacilli	Staphylococcales	Staphylococcaceae	Staphylococcus	aureus
TPS	ASV_dlu_bk7	0,016050723	0,665092109	0,960981129	Bacteria	Proteobacteria	Gammaproteobacteria	Burkholderiales	Neisseriaceae	NA	NA
TPS	ASV_dmt_o1x	-0,21663887	0,283169557	0,831923437	Bacteria	Proteobacteria	Gammaproteobacteria	Enterobacterales	Pasteurellaceae	Haemophilus	haemolyticus/influenzae
TPS	ASV_dui_4x1	-0,231837817	0,276531771	0,815785804	Bacteria	Firmicutes	Bacilli	Lactobacillales	Carnobacteriaceae	Dolosigranulum	NA
TPS	ASV_dz0_fy2	-0,054125946	0,589879074	0,943187185	Bacteria	Actinobacteriota	Actinobacteria	Corynebacteriales	Corynebacteriaceae	Corynebacterium	NA
TPS	ASV_e7g_uqv	-0,063663571	0,531033584	0,92013237	Bacteria	Actinobacteriota	Thermoleophilia	Gaiellales	NA	NA	NA
TPS	ASV_e8x_8cs	0,019336145	0,767834256	0,974143729	Bacteria	Actinobacteriota	Actinobacteria	Corynebacteriales	Corynebacteriaceae	Lawsonella	NA
TPS	ASV_egx_g4e	0,076273159	0,454073263	0,914654597	Bacteria	Proteobacteria	Gammaproteobacteria	Enterobacterales	NA	NA	NA
TPS	ASV_elz_ffm	-0,102296676	0,498459873	0,931886816	Bacteria	Actinobacteriota	Actinobacteria	Corynebacteriales	Corynebacteriaceae	Corynebacterium	NA

Variable	FeatureID	spearman.erho	spearman.ep	spearman.eBH	Domain	Phylum	Class	Order	Family	Genus	Species
TPS	ASV_f1j_s2e	0,182574008	0,356152955	0,884895698	Bacteria	Actinobacteriota	Actinobacteria	Corynebacteriales	Corynebacteriaceae	Corynebacterium	aurimucosum/simulans/striatum/xerosis
TPS	ASV_f6c_v0m	-0,202426788	0,28799964	0,84678076	Bacteria	Actinobacteriota	Actinobacteria	Corynebacteriales	Corynebacteriaceae	Corynebacterium	NA
TPS	ASV_f8c_ir4	-0,043635486	0,506645097	0,924335546	Bacteria	Bacteroidota	Bacteroidia	Bacteroidales	Bacteroidaceae	Bacteroides	plebeius
TPS	ASV_fb6_dje	0,026687614	0,525005909	0,93536127	Bacteria	Firmicutes	Bacilli	Staphylococcales	Staphylococcaceae	Staphylococcus	aureus/devriesei/epidermidis/haemolyticus/hominis
TPS	ASV_fc7_hpm	-0,049190593	0,486830791	0,918228118	Bacteria	Actinobacteriota	Actinobacteria	Corynebacteriales	Corynebacteriaceae	Corynebacterium	NA
TPS	ASV_ffj_wqa	0,019806375	0,484121674	0,927120433	Bacteria	Proteobacteria	Gammaproteobacteria	Pseudomonadales	Moraxellaceae	Acinetobacter	NA
TPS	ASV_fgq_ohv	0,069359953	0,557610978	0,934639582	Bacteria	Actinobacteriota	Actinobacteria	Corynebacteriales	Corynebacteriaceae	Corynebacterium	NA
TPS	ASV_fmb_men	-0,066061125	0,519245262	0,938304475	Bacteria	Actinobacteriota	Actinobacteria	Corynebacteriales	Corynebacteriaceae	Corynebacterium	NA
TPS	ASV_fqm_8pm	-0,157000097	0,405982107	0,914978382	Bacteria	Firmicutes	Bacilli	Lactobacillales	Streptococcaceae	Streptococcus	anginosus/cristatus/infantis/mitis/oralis/pneumoniae/pseudopneumoniae/sanguinis/timonensis
TPS	ASV_fug_ldj	0,05177892	0,565490179	0,930945425	Bacteria	Bacteroidota	Bacteroidia	Bacteroidales	Prevotellaceae	Prevotella_9	NA
TPS	ASV_g3d_cs4	0,02010955	0,524840795	0,926725957	Bacteria	Proteobacteria	Gammaproteobacteria	Enterobacterales	Pasteurellaceae	Haemophilus	influenzae
TPS	ASV_geq_907	0,027936404	0,817448319	0,976243817	Bacteria	Firmicutes	Clostridia	Peptostreptococcales-Tissierellales	Family XI	Anaerococcus	octavius
TPS	ASV_gfz_fyn	-0,039135303	0,533788648	0,93373401	Bacteria	Proteobacteria	Gammaproteobacteria	Enterobacterales	Enterobacteriaceae	NA	NA
TPS	ASV_gg8_mtq	-0,015115419	0,513421143	0,919476762	Bacteria	Bacteroidota	Bacteroidia	Bacteroidales	Muribaculaceae	NA	NA
TPS	ASV_gzb_mis	-0,056175983	0,438545114	0,91464977	Bacteria	Firmicutes	Clostridia	Lachnospirales	Lachnospiraceae	Coprococcus	catus
TPS	ASV_h1r_zyt	0,092813705	0,505225642	0,913562519	Bacteria	Fusobacteriota	Fusobacteriia	Fusobacteriales	Fusobacteriaceae	Fusobacterium	NA
TPS	ASV_h40_0md	0,033202774	0,620078553	0,951787795	Bacteria	Actinobacteriota	Actinobacteria	Corynebacteriales	Corynebacteriaceae	Corynebacterium	NA
TPS	ASV_h4w_6wi	0,040566617	0,507250676	0,926031949	Bacteria	Firmicutes	Bacilli	Staphylococcales	Staphylococcaceae	NA	NA
TPS	ASV_h8v_sx4	0,036766622	0,54453046	0,925610851	Bacteria	Actinobacteriota	Actinobacteria	Corynebacteriales	Nocardiaceae	Millisia	NA
TPS	ASV_hbi_ebs	0,12600101	0,422905463	0,89390264	Bacteria	Actinobacteriota	Actinobacteria	Corynebacteriales	Corynebacteriaceae	Lawsonella	clevelandensis
TPS	ASV_het_bu3	0,093264342	0,493650979	0,909134588	Bacteria	Campylobacterota	Campylobacteria	Campylobacterales	Campylobacteraceae	Campylobacter	NA
TPS	ASV_hjn_92c	0,075997827	0,660028204	0,964701423	Bacteria	Firmicutes	Clostridia	Peptostreptococcales-Tissierellales	Family XI	Finegoldia	magna
TPS	ASV_hkw_6s3	0,090650234	0,479103674	0,901922357	Bacteria	Actinobacteriota	Actinobacteria	Corynebacteriales	Corynebacteriaceae	Corynebacterium	NA
TPS	ASV_hpm_svr	0,047848994	0,581272077	0,944863225	Bacteria	Actinobacteriota	Actinobacteria	Corynebacteriales	Corynebacteriaceae	Corynebacterium	NA
TPS	ASV_hq8_93x	0,057975438	0,569909383	0,94257741	Bacteria	Actinobacteriota	Actinobacteria	Corynebacteriales	Corynebacteriaceae	Corynebacterium	NA
TPS	ASV_hqq_2ph	0,074982089	0,474978935	0,917472098	Bacteria	Proteobacteria	Gammaproteobacteria	Burkholderiales	Oxalobacteraceae	Massilia	timonae
TPS	ASV_i0i_mxo	-0,170069603	0,382324679	0,87973208	Bacteria	Actinobacteriota	Actinobacteria	Corynebacteriales	Corynebacteriaceae	Corynebacterium	pyruviciproducens
TPS	ASV_i35_0op	0,106308068	0,549003168	0,948500004	Bacteria	Actinobacteriota	Actinobacteria	Corynebacteriales	Corynebacteriaceae	Corynebacterium	aurimucosum/jeikeium/propinquum/pseudodiphtheriticum
TPS	ASV_i88_mm0	0,034425784	0,559190845	0,945417298	Bacteria	Actinobacteriota	Actinobacteria	Corynebacteriales	Corynebacteriaceae	Corynebacterium	NA
TPS	ASV_ic5_nai	-0,023130984	0,552988756	0,938175501	Bacteria	Firmicutes	Bacilli	Lactobacillales	Streptococcaceae	Streptococcus	sobrinus
TPS	ASV_igm_m49	0,069074309	0,518869636	0,931985209	Bacteria	NA	NA	NA	NA	NA	NA

Variable	FeatureID	spearman.erho	spearman.ep	spearman.eBH	Domain	Phylum	Class	Order	Family	Genus	Species
TPS	ASV_ii5_tjz	0,121385332	0,455893263	0,921995491	Bacteria	Firmicutes	Bacilli	Staphylococcales	Staphylococcaceae	Staphylococcus	NA
TPS	ASV_ikg_ra0	0,015747548	0,635110152	0,959487538	Bacteria	Actinobacteriota	Actinobacteria	Corynebacteriales	Corynebacteriaceae	Corynebacterium	NA
TPS	ASV_iov_l70	-0,242632482	0,246688171	0,816303212	Bacteria	Firmicutes	Bacilli	Lactobacillales	Carnobacteriaceae	Dolosigranulum	pigrum
TPS	ASV_iqo_ea7	-0,036240707	0,534675311	0,934091565	Bacteria	Firmicutes	Bacilli	Staphylococcales	Staphylococcaceae	Staphylococcus	NA
TPS	ASV_jjw_h6c	-0,351883612	0,037063891	0,710511133	Bacteria	Actinobacteriota	Actinobacteria	Corynebacteriales	Corynebacteriaceae	Corynebacterium	NA
TPS	ASV_jl5_6pn	0,078503658	0,575349918	0,947815576	Bacteria	Firmicutes	Bacilli	Staphylococcales	Staphylococcaceae	Staphylococcus	aureus
TPS	ASV_jp2_wik	-0,235343918	0,188152826	0,861147537	Bacteria	Firmicutes	Bacilli	Staphylococcales	Staphylococcaceae	Staphylococcus	epidermidis
TPS	ASV_jph_cup	0,050844648	0,633649123	0,958020804	Bacteria	Actinobacteriota	Actinobacteria	Corynebacteriales	Corynebacteriaceae	Corynebacterium	NA
TPS	ASV_jy3_f61	-0,006255296	0,586330551	0,945311742	Bacteria	Actinobacteriota	Actinobacteria	Corynebacteriales	Corynebacteriaceae	Corynebacterium	NA
TPS	ASV_jy9_jqd	-0,044065499	0,485835947	0,919959505	Bacteria	Firmicutes	Clostridia	Lachnospirales	Lachnospiraceae	[Eubacterium] ventriosum group	NA
TPS	ASV_k2s_cqm	-0,05478798	0,599994107	0,948232892	Bacteria	Actinobacteriota	Actinobacteria	Corynebacteriales	Corynebacteriaceae	Corynebacterium	NA
TPS	ASV_k3h_3z5	-0,042174267	0,530697984	0,929332914	Bacteria	Proteobacteria	Gammaproteobacteria	Xanthomonadales	Xanthomonadaceae	Lysobacter	NA
TPS	ASV_k3h_qcz	-0,11144863	0,451941283	0,91505149	Bacteria	Firmicutes	Bacilli	Staphylococcales	Staphylococcaceae	Staphylococcus	NA
TPS	ASV_kaj_b2n	0,007887695	0,533875939	0,927236921	Bacteria	Firmicutes	Bacilli	Lactobacillales	P5D1-392	NA	NA
TPS	ASV_kcs_cbi	-0,003281298	0,561329504	0,942058827	Bacteria	Actinobacteriota	Actinobacteria	Corynebacteriales	Corynebacteriaceae	Corynebacterium	NA
TPS	ASV_kdo_fhg	0,035794195	0,496843846	0,909320923	Bacteria	Proteobacteria	Gammaproteobacteria	Burkholderiales	Comamonadaceae	Mitsuaria	NA
TPS	ASV_ki3_dfk	-0,036388169	0,594933152	0,95419637	Bacteria	Actinobacteriota	Actinobacteria	Corynebacteriales	Corynebacteriaceae	Corynebacterium	NA
TPS	ASV_kkl_mxy	0,086716183	0,572371301	0,949725081	Bacteria	Actinobacteriota	Actinobacteria	Corynebacteriales	Corynebacteriaceae	Corynebacterium	kroppenstedtii
TPS	ASV_kru_fff	0,043567426	0,49883971	0,926865179	Bacteria	Proteobacteria	Gammaproteobacteria	Enterobacterales	Erwiniaceae	NA	NA
TPS	ASV_kta_7ms	0,038861002	0,508857428	0,923718906	Bacteria	Proteobacteria	Gammaproteobacteria	Burkholderiales	Burkholderiaceae	Burkholderia-Caballeronia-Paraburkholderia	NA
TPS	ASV_ktj_lyl	0,088229994	0,502627436	0,916844029	Bacteria	Proteobacteria	Gammaproteobacteria	Burkholderiales	Neisseriaceae	NA	NA
TPS	ASV_l3n_w4d	0,083702999	0,493196343	0,917146659	Bacteria	Synergistota	Synergistia	Synergistales	Synergistaceae	Jonquetella	anthropi
TPS	ASV_lky_mqj	0,093531424	0,46880498	0,92324901	Bacteria	Firmicutes	Bacilli	Lactobacillales	Streptococcaceae	Streptococcus	NA
TPS	ASV_lmq_jff	-0,265795434	0,210459229	0,77931583	Bacteria	Firmicutes	Bacilli	Lactobacillales	Carnobacteriaceae	Dolosigranulum	NA
TPS	ASV_lnh_9fs	-0,007805199	0,578961598	0,944556645	Bacteria	Actinobacteriota	Actinobacteria	Corynebacteriales	Corynebacteriaceae	Corynebacterium	afermentans/coyleae
TPS	ASV_lvd_sr9	-0,043786042	0,627570641	0,959264257	Bacteria	Actinobacteriota	Actinobacteria	Corynebacteriales	Corynebacteriaceae	Corynebacterium	NA
TPS	ASV_lvh_wn0	-0,06881857	0,569934213	0,941488449	Bacteria	Actinobacteriota	Actinobacteria	Micrococcales	Dermabacteraceae	Dermabacter	jinjuensis
TPS	ASV_m52_gpv	0,067856455	0,513377178	0,926893395	Bacteria	Actinobacteriota	Actinobacteria	Corynebacteriales	Corynebacteriaceae	Corynebacterium	NA
TPS	ASV_mbn_3b6	-0,038439238	0,516212623	0,921193865	Bacteria	Firmicutes	Clostridia	Lachnospirales	Lachnospiraceae	[Ruminococcus] torques group	NA
TPS	ASV_mc2_eno	-0,014556505	0,540289838	0,937555882	Bacteria	Proteobacteria	Gammaproteobacteria	Xanthomonadales	Xanthomonadaceae	Lysobacter	NA
TPS	ASV_mcc_ype	-0,050586846	0,60037965	0,951782262	Bacteria	Firmicutes	Bacilli	Staphylococcales	Staphylococcaceae	Staphylococcus	epidermidis
TPS	ASV_mdt_jke	0,067095425	0,485760586	0,922870503	Bacteria	Proteobacteria	Gammaproteobacteria	Enterobacterales	Pasteurellaceae	Haemophilus	haemolyticus/influenzae

Variable	FeatureID	spearman.erho	spearman.ep	spearman.eBH	Domain	Phylum	Class	Order	Family	Genus	Species
TPS	ASV_mgr_u9o	-0,139481968	0,411289224	0,909200869	Bacteria	Actinobacteriota	Actinobacteria	Corynebacteriales	Corynebacteriaceae	Corynebacterium	NA
TPS	ASV_miq_ryo	-0,035152785	0,46259913	0,907998528	Bacteria	Firmicutes	Bacilli	Staphylococcales	Staphylococcaceae	Staphylococcus	NA
TPS	ASV_mlb_2sd	-0,014852461	0,61273598	0,955152929	Bacteria	Firmicutes	Bacilli	Staphylococcales	Staphylococcaceae	Staphylococcus	NA
TPS	ASV_mm9_js5	0,101358278	0,538193454	0,92514297	Bacteria	Firmicutes	Bacilli	Staphylococcales	Staphylococcaceae	Staphylococcus	aureus
TPS	ASV_mvn_oem	-0,061322733	0,565671431	0,941239003	Bacteria	Actinobacteriota	Actinobacteria	Corynebacteriales	Corynebacteriaceae	Corynebacterium	NA
TPS	ASV_mx9_gyi	0,038584638	0,540866583	0,935505545	Bacteria	Bacteroidota	Bacteroidia	Bacteroidales	Bacteroidaceae	Bacteroides	uniformis
TPS	ASV_n1a_pw6	0,118084441	0,454373837	0,90666891	Bacteria	Proteobacteria	Alphaproteobacteria	Rhizobiales	Methyloligellaceae	NA	NA
TPS	ASV_n5f_h6y	0,016627167	0,546244101	0,938321135	Bacteria	Proteobacteria	Gammaproteobacteria	Burkholderiales	Oxalobacteraceae	Massilia	NA
TPS	ASV_n5j_9sq	-0,006015025	0,522834235	0,930177965	Bacteria	Firmicutes	Bacilli	Staphylococcales	Staphylococcaceae	Staphylococcus	NA
TPS	ASV_n79_65k	0,001533403	0,564269295	0,946784871	Bacteria	Proteobacteria	Gammaproteobacteria	Burkholderiales	Neisseriaceae	NA	NA
TPS	ASV_nbq_6pg	0,0098408	0,508051069	0,92223715	Bacteria	Firmicutes	Clostridia	Peptostreptococcales-Tissierellales	Family XI	Anaerococcus	prevotii/tetradius
TPS	ASV_nk5_hwz	0,06008941	0,508186977	0,932677073	Bacteria	Bacteroidota	Bacteroidia	Flavobacteriales	Weeksellaceae	Cloacibacterium	normanense
TPS	ASV_ntr_hh5	0,000814653	0,52982135	0,924509074	Bacteria	Proteobacteria	Gammaproteobacteria	Enterobacterales	Pasteurellaceae	Haemophilus	haemolyticus/influenzae/parainfluenzae
TPS	ASV_nvc_oao	0,127773654	0,478231413	0,935241331	Bacteria	Deinococcota	Deinococci	Thermales	Thermaceae	Thermus	parvatiensis/thermophilus
TPS	ASV_nvm_lyw	0,045088455	0,50787173	0,931090635	Bacteria	Firmicutes	Clostridia	Oscillospirales	Oscillospiraceae	Intestinimonas	NA
TPS	ASV_o0r_len	-0,256101065	0,16053502	0,838364301	Bacteria	Firmicutes	Bacilli	Staphylococcales	Staphylococcaceae	Staphylococcus	aureus/capitis/caprae/epidermidis/haemolyticus/saprophyticus/warneri
TPS	ASV_o10_2p1	0,008699255	0,52988695	0,932565513	Bacteria	Proteobacteria	Gammaproteobacteria	Pseudomonadales	Pseudomonadaceae	Pseudomonas	brassicacearum/chlororaphis/corrugata/fluorescens/jessenii/kilonensis/mohnii/putida/synxantha
TPS	ASV_o1f_10k	0,072462852	0,501539001	0,908156027	Bacteria	Proteobacteria	Gammaproteobacteria	Burkholderiales	Comamonadaceae	NA	NA
TPS	ASV_o5q_r99	0,007812417	0,517074035	0,924680262	Bacteria	Actinobacteriota	Actinobacteria	Corynebacteriales	Corynebacteriaceae	Corynebacterium	NA
TPS	ASV_ode_i48	0,071188282	0,490779579	0,910203459	Bacteria	Actinobacteriota	Actinobacteria	Corynebacteriales	Corynebacteriaceae	Corynebacterium	NA
TPS	ASV_on0_uxs	0,008432172	0,625391401	0,958532747	Bacteria	Actinobacteriota	Actinobacteria	Corynebacteriales	Corynebacteriaceae	Corynebacterium	afermentans/ihumii
TPS	ASV_onw_luq	0,102627693	0,48759902	0,895342989	Bacteria	Proteobacteria	Gammaproteobacteria	Burkholderiales	Neisseriaceae	Eikenella	NA
TPS	ASV_ouf_s4v	-0,105585192	0,457863795	0,908937634	Bacteria	Actinobacteriota	Actinobacteria	Corynebacteriales	Corynebacteriaceae	Corynebacterium	NA
TPS	ASV_oum_efh	-0,027913718	0,522518012	0,930971866	Bacteria	Firmicutes	Bacilli	Staphylococcales	Staphylococcaceae	Staphylococcus	NA
TPS	ASV_oxw_74i	0,042576437	0,496854225	0,923879237	Bacteria	Proteobacteria	Alphaproteobacteria	Acetobacterales	Acetobacteraceae	Roseomonas	NA
TPS	ASV_oyd_63r	0,022432857	0,573142306	0,947147786	Bacteria	Firmicutes	Bacilli	Staphylococcales	Staphylococcaceae	Staphylococcus	NA
TPS	ASV_p3d_k08	0,027407396	0,557888687	0,939397526	Bacteria	Firmicutes	Clostridia	Peptostreptococcales-Tissierellales	Family XI	W5053	NA
TPS	ASV_p46_336	0,081613775	0,502475416	0,917757574	Bacteria	Proteobacteria	Gammaproteobacteria	Enterobacterales	Enterobacteriaceae	Escherichia-Shigella	NA
TPS	ASV_p9u_72k	0,078028272	0,470020792	0,904830333	Bacteria	Firmicutes	Clostridia	Peptococcales	Peptococcaceae	Peptococcus	NA
TPS	ASV_paz_uyl	-0,044535729	0,733434609	0,967532877	Bacteria	Actinobacteriota	Actinobacteria	Propionibacteriales	Propionibacteriaceae	Cutibacterium	acnes/avidum
TPS	ASV_pgj_6t5	-0,001186918	0,499889183	0,916424457	Bacteria	Actinobacteriota	Actinobacteria	Corynebacteriales	Corynebacteriaceae	Corynebacterium	NA

Variable	FeatureID	spearman.erho	spearman.ep	spearman.eBH	Domain	Phylum	Class	Order	Family	Genus	Species
TPS	ASV_pix_o0d	0,08883428	0,508123553	0,921568853	Bacteria	Desulfobacterota	Desulfovibrionia	Desulfovibrionales	Desulfohalobiaceae	Desulfovermiculus	NA
TPS	ASV_pjp_yzq	-0,010359496	0,555734493	0,942375431	Bacteria	Firmicutes	Bacilli	Staphylococcales	Staphylococcaceae	Staphylococcus	NA
TPS	ASV_pni_ryk	0,075301763	0,477467662	0,917843328	Bacteria	Actinobacteriota	Actinobacteria	Corynebacteriales	Corynebacteriaceae	Corynebacterium	NA
TPS	ASV_pou_qqz	0,003888678	0,532030768	0,929653744	Bacteria	Actinobacteriota	Actinobacteria	Micrococcales	Dermacoccaceae	Barrientosiimonas	humi/marina
TPS	ASV_pwy_pjt	0,038652698	0,582099716	0,952792193	Bacteria	Actinobacteriota	Actinobacteria	Corynebacteriales	Corynebacteriaceae	Corynebacterium	NA
TPS	ASV_q1f_0em	-0,05377946	0,556692544	0,941878145	Bacteria	Actinobacteriota	Actinobacteria	Corynebacteriales	Corynebacteriaceae	Corynebacterium	NA
TPS	ASV_q1k_ad2	0,150771612	0,422179818	0,907863918	Bacteria	Firmicutes	Bacilli	Staphylococcales	Staphylococcaceae	Staphylococcus	NA
TPS	ASV_q1o_24k	-0,080008187	0,645723411	0,964696493	Bacteria	Actinobacteriota	Actinobacteria	Corynebacteriales	Corynebacteriaceae	Corynebacterium	accolens/fastidiosum
TPS	ASV_q1r_s4c	0,092711615	0,502391975	0,916343449	Bacteria	Actinobacteriota	Actinobacteria	Actinomycetales	Actinomycetaceae	Actinotignum	schaalii/timonense
TPS	ASV_qc1_pgs	0,003188489	0,534841989	0,93511521	Bacteria	Firmicutes	Bacilli	Staphylococcales	Staphylococcaceae	Staphylococcus	NA
TPS	ASV_qc8_76v	0,001501436	0,555364166	0,942831972	Bacteria	Proteobacteria	Gammaproteobacteria	Enterobacterales	Pasteurellaceae	Haemophilus	influenzae
TPS	ASV_qd8_nzn	-0,003862898	0,549786928	0,925661797	Bacteria	Actinobacteriota	Actinobacteria	Corynebacteriales	Corynebacteriaceae	Corynebacterium	jeikeium
TPS	ASV_qft_24l	0,117180073	0,449491395	0,899543058	Bacteria	Bacteroidota	Bacteroidia	Bacteroidales	Prevotellaceae	Alloprevotella	NA
TPS	ASV_qis_xxl	-0,039040432	0,617118367	0,955807651	Bacteria	Firmicutes	Clostridia	Peptostreptococcales-Tissierellales	Family XI	Anaerococcus	nagyae
TPS	ASV_qk1_qy8	0,057389713	0,53967946	0,936997533	Bacteria	Proteobacteria	Gammaproteobacteria	Enterobacterales	Pasteurellaceae	Lonepinella	NA
TPS	ASV_r2m_1o3	-0,011525791	0,525228775	0,930382928	Bacteria	Firmicutes	Bacilli	Staphylococcales	Staphylococcaceae	Staphylococcus	NA
TPS	ASV_r73_72u	-0,072090587	0,484146161	0,923054245	Bacteria	Actinobacteriota	Actinobacteria	Corynebacteriales	Corynebacteriaceae	Corynebacterium	NA
TPS	ASV_r8p_k1e	0,220233655	0,251691133	0,862667994	Bacteria	Firmicutes	Bacilli	Staphylococcales	Staphylococcaceae	Staphylococcus	aureus
TPS	ASV_r9n_48y	-0,02698357	0,494101336	0,920421679	Bacteria	Firmicutes	Bacilli	Staphylococcales	Staphylococcaceae	Staphylococcus	NA
TPS	ASV_rad_25g	0,043828321	0,5196777	0,934388375	Bacteria	Proteobacteria	Gammaproteobacteria	Enterobacterales	Pasteurellaceae	NA	NA
TPS	ASV_raj_rv1	-0,31350109	0,064682525	0,766442436	Bacteria	Firmicutes	Bacilli	Staphylococcales	Staphylococcaceae	Staphylococcus	aureus/capitis/caprae/epidermidis/haemolyticus/warneri
TPS	ASV_rat_2jx	0,05974705	0,529810611	0,912882993	Bacteria	Proteobacteria	Gammaproteobacteria	Enterobacterales	Enterobacteriaceae	Escherichia-Shigella	NA
TPS	ASV_rmx_t9x	0,061610439	0,617133432	0,954806112	Bacteria	Firmicutes	Bacilli	Staphylococcales	Staphylococcaceae	Staphylococcus	aureus
TPS	ASV_rs1_k0s	0,00935407	0,542070604	0,935151167	Bacteria	Firmicutes	Bacilli	Staphylococcales	Staphylococcaceae	Staphylococcus	NA
TPS	ASV_rtx_tpz	0,079301811	0,523752639	0,92783913	Bacteria	Proteobacteria	Gammaproteobacteria	Pseudomonadales	Moraxellaceae	Moraxella	catarrhalis/nonliquefaciens
TPS	ASV_s8r_baf	-0,261032293	0,178408871	0,822661108	Bacteria	Firmicutes	Clostridia	Peptostreptococcales-Tissierellales	Family XI	Anaerococcus	NA
TPS	ASV_sjv_qdw	-0,039263172	0,526424891	0,931323685	Bacteria	Actinobacteriota	Actinobacteria	Corynebacteriales	Corynebacteriaceae	Corynebacterium	NA
TPS	ASV_snw_j03	-0,040502682	0,493998647	0,925425479	Bacteria	Firmicutes	Bacilli	Lactobacillales	Streptococcaceae	Streptococcus	NA
TPS	ASV_ssr_vha	0,076971285	0,611757123	0,951172502	Bacteria	Firmicutes	Bacilli	Staphylococcales	Staphylococcaceae	Staphylococcus	aureus
TPS	ASV_sza_hre	-0,030956807	0,483749316	0,925840513	Bacteria	Actinobacteriota	Actinobacteria	Corynebacteriales	Corynebacteriaceae	NA	NA
TPS	ASV_szb_rz6	0,068662858	0,469490878	0,908607441	Bacteria	Firmicutes	Bacilli	Lactobacillales	Aerococcaceae	Facklamia	languida
TPS	ASV_t7p_n06	-0,002393429	0,578101473	0,950608463	Bacteria	Firmicutes	Clostridia	Peptostreptococcales-Tissierellales	Family XI	Anaerococcus	NA
TPS	ASV_t8l_ysz	0,1645609	0,380587758	0,913087245	Bacteria	Firmicutes	Bacilli	Staphylococcales	Staphylococcaceae	NA	NA

Variable	FeatureID	spearman.erho	spearman.ep	spearman.eBH	Domain	Phylum	Class	Order	Family	Genus	Species
TPS	ASV_ten_mjo	-0,032175693	0,528413717	0,931579155	Bacteria	Actinobacteriota	Actinobacteria	Corynebacteriales	Corynebacteriaceae	Corynebacterium	NA
TPS	ASV_tu2_xgy	0,008941588	0,622162627	0,957761636	Bacteria	Actinobacteriota	Actinobacteria	Corynebacteriales	Corynebacteriaceae	Corynebacterium	NA
TPS	ASV_tvh_ar7	0,094530663	0,475913324	0,922729668	Bacteria	Firmicutes	Bacilli	Lactobacillales	Streptococcaceae	Streptococcus	anginosus/constellatus/intermedius/lutetiensis
TPS	ASV_v7r_7sn	0,09771709	0,441112781	0,90671117	Bacteria	Proteobacteria	Gammaproteobacteria	Burkholderiales	Comamonadaceae	Rubrivivax	NA
TPS	ASV_xex_kso	0,005863438	0,506864601	0,920798237	Bacteria	Actinobacteriota	Actinobacteria	Micrococcales	Bogoriellaceae	Georgenia	NA
SNOT22	ASV_17p_uuy	-0,064701045	0,659821018	0,921093287	Bacteria	Actinobacteriota	Actinobacteria	Corynebacteriales	Corynebacteriaceae	Corynebacterium	NA
SNOT22	ASV_19p_rjx	-0,157171436	0,42989827	0,771931929	Bacteria	Bacteroidota	Bacteroidia	Bacteroidales	Prevotellaceae	Prevotella_9	NA
SNOT22	ASV_1af_96k	-0,034221969	0,503020953	0,847621547	Bacteria	Firmicutes	Bacilli	Lactobacillales	Carnobacteriaceae	Dolosigranulum	NA
SNOT22	ASV_1dc_ewy	0,174197876	0,398491268	0,757054377	Bacteria	Firmicutes	Bacilli	Lactobacillales	Carnobacteriaceae	Dolosigranulum	NA
SNOT22	ASV_1kn_zel	-0,000547391	0,713726245	0,939094852	Bacteria	Actinobacteriota	Actinobacteria	Corynebacteriales	Corynebacteriaceae	Lawsonella	NA
SNOT22	ASV_1ob_3tg	0,05478209	0,50485103	0,857783093	Bacteria	Firmicutes	Bacilli	Staphylococcales	Staphylococcaceae	Staphylococcus	NA
SNOT22	ASV_1wh_81x	0,069032885	0,509927526	0,850366499	Bacteria	Firmicutes	Negativicutes	Veillonellales-Selenomonadales	Veillonellaceae	Negativicoccus	succinicivorans
SNOT22	ASV_20k_5qe	-0,053255987	0,435339043	0,809773291	Bacteria	Actinobacteriota	Actinobacteria	Corynebacteriales	Nocardiaceae	Millisia	NA
SNOT22	ASV_242_lzz	-0,051371644	0,773244498	0,96036635	Bacteria	Actinobacteriota	Actinobacteria	Corynebacteriales	Corynebacteriaceae	Corynebacterium	aurimucosum/pseudogenitalium/tuberculostearicum
SNOT22	ASV_2d3_euj	0,389854172	0,052221765	0,410927886	Bacteria	Actinobacteriota	Actinobacteria	Corynebacteriales	Corynebacteriaceae	Corynebacterium	NA
SNOT22	ASV_2db_0um	-0,171684462	0,39689812	0,764808469	Bacteria	Firmicutes	Negativicutes	Veillonellales-Selenomonadales	Veillonellaceae	Dialister	NA
SNOT22	ASV_2ee_ogi	-0,090558822	0,53531872	0,865801211	Bacteria	Firmicutes	Bacilli	Staphylococcales	Staphylococcaceae	Staphylococcus	lugdunensis
SNOT22	ASV_2iq_1yo	-0,160230807	0,402302021	0,784228876	Bacteria	Firmicutes	Clostridia	Peptococcales	Peptococcaceae	Peptococcus	NA
SNOT22	ASV_2o1_p31	-0,009593672	0,555699705	0,875388384	Bacteria	Firmicutes	Clostridia	Lachnospirales	Lachnospiraceae	Lachnospiraceae AC2044 group	bacterium
SNOT22	ASV_2x6_j18	-0,049028753	0,57539193	0,874654624	Bacteria	Firmicutes	Clostridia	Peptostreptococcales-Tissierellales	Peptostreptococcaceae	Criibacterium	NA
SNOT22	ASV_2xa_4rw	-0,014597571	0,582126833	0,875854944	Bacteria	Bacteroidota	Bacteroidia	Bacteroidales	Prevotellaceae	Prevotella_9	NA
SNOT22	ASV_2xm_w96	-0,14664204	0,410665522	0,785997864	Bacteria	Firmicutes	Clostridia	Lachnospirales	Lachnospiraceae	Dorea	formicigenerans
SNOT22	ASV_30c_6sw	-0,141060658	0,390163412	0,770152348	Bacteria	Proteobacteria	Gammaproteobacteria	Burkholderiales	Oxalobacteraceae	Massilia	timonae
SNOT22	ASV_3em_n0d	-0,053734596	0,502681073	0,834409982	Bacteria	Firmicutes	Bacilli	Lactobacillales	Lactobacillaceae	Liquorilactobacillus	NA
SNOT22	ASV_3en_7ow	0,266658236	0,225251807	0,635150488	Bacteria	Firmicutes	Bacilli	Staphylococcales	Gemellaceae	Gemella	morbillorum
SNOT22	ASV_3in_1yl	-0,091498844	0,475088296	0,822787286	Bacteria	Firmicutes	Bacilli	Staphylococcales	Staphylococcaceae	NA	NA
SNOT22	ASV_3kg_4bk	0,161123542	0,434354881	0,798438742	Bacteria	Firmicutes	Bacilli	Lactobacillales	Carnobacteriaceae	Dolosigranulum	pigrum
SNOT22	ASV_3kh_nw9	0,345244671	0,11706142	0,496825101	Bacteria	Actinobacteriota	Actinobacteria	Corynebacteriales	Corynebacteriaceae	Corynebacterium	NA
SNOT22	ASV_3pn_tj1	-0,090113171	0,483372213	0,827615064	Bacteria	Actinobacteriota	Actinobacteria	Corynebacteriales	Corynebacteriaceae	Corynebacterium	NA
SNOT22	ASV_3po_bb2	-0,096274902	0,473472099	0,811382252	Bacteria	Actinobacteriota	Actinobacteria	Corynebacteriales	Corynebacteriaceae	Corynebacterium	NA
SNOT22	ASV_3tk_6ez	-0,046301828	0,529484737	0,864890307	Bacteria	Actinobacteriota	Actinobacteria	Corynebacteriales	Corynebacteriaceae	Corynebacterium	NA

Variable	FeatureID	spearman.erho	spearman.ep	spearman.eBH	Domain	Phylum	Class	Order	Family	Genus	Species
SNOT22	ASV_3tp_m3o	-0,082684702	0,472715455	0,817319256	Bacteria	Firmicutes	Bacilli	Staphylococcales	Staphylococcaceae	Staphylococcus	NA
SNOT22	ASV_3w7_jlb	-0,085355741	0,477476567	0,829757947	Bacteria	Firmicutes	Bacilli	Staphylococcales	Staphylococcaceae	NA	NA
SNOT22	ASV_440_o98	-0,0279499	0,556228822	0,875935576	Bacteria	Firmicutes	Clostridia	Peptostreptococcales-Tissierellales	Family XI	Anaerococcus	NA
SNOT22	ASV_46z_g65	-0,146654937	0,430739544	0,782469646	Bacteria	Firmicutes	Negativicutes	Veillonellales-Selenomonadales	Veillonellaceae	Negativicoccus	NA
SNOT22	ASV_47r_9ra	0,066669933	0,470512443	0,826928341	Bacteria	Actinobacteriota	Actinobacteria	Corynebacteriales	Corynebacteriaceae	Corynebacterium	NA
SNOT22	ASV_49k_gjb	-0,15813152	0,407921475	0,773852217	Bacteria	Firmicutes	Clostridia	Oscillospirales	Oscillospiraceae	UCG-002	NA
SNOT22	ASV_4b4_zwy	0,156414833	0,427895212	0,781989457	Bacteria	Campylobacterota	Campylobacteria	Campylobacterales	Campylobacteraceae	Campylobacter	ureolyticus
SNOT22	ASV_4cz_ka1	-0,055012797	0,505880432	0,847035717	Bacteria	Firmicutes	Bacilli	Staphylococcales	Staphylococcaceae	Staphylococcus	NA
SNOT22	ASV_4kw_hck	-0,137105686	0,409429024	0,790201266	Bacteria	Firmicutes	Clostridia	Peptostreptococcales-Tissierellales	Family XI	Anaerococcus	NA
SNOT22	ASV_4m8_kdu	-0,167729491	0,397027682	0,758120405	Bacteria	Bacteroidota	Bacteroidia	Bacteroidales	Prevotellaceae	Prevotella_9	NA
SNOT22	ASV_4n2_dht	0,004028053	0,557548889	0,870538496	Bacteria	Bacteroidota	Bacteroidia	Chitinophagales	Chitinophagaceae	Chitinophaga	humicola
SNOT22	ASV_4ni_ro3	-0,314487601	0,164170804	0,54463111	Bacteria	Actinobacteriota	Actinobacteria	Corynebacteriales	Corynebacteriaceae	Corynebacterium	NA
SNOT22	ASV_4nj_u2c	-0,067534008	0,475395946	0,835326106	Bacteria	Actinobacteriota	Actinobacteria	Corynebacteriales	Corynebacteriaceae	NA	NA
SNOT22	ASV_4pz_tc7	0,017774445	0,847139073	0,970213745	Bacteria	Firmicutes	Bacilli	Staphylococcales	Staphylococcaceae	Staphylococcus	argenteus/aureus/equorum/phage/schweitzeri/simiae
SNOT22	ASV_4qr_yd2	-0,039161385	0,522449661	0,848041962	Bacteria	Firmicutes	Bacilli	Lactobacillales	Streptococcaceae	Streptococcus	NA
SNOT22	ASV_4rd_dxc	0,07058908	0,631830202	0,909434855	Bacteria	Actinobacteriota	Actinobacteria	Corynebacteriales	Corynebacteriaceae	Corynebacterium	NA
SNOT22	ASV_513_wtm	-0,066596852	0,477965466	0,827655457	Bacteria	Proteobacteria	Gammaproteobacteria	Enterobacterales	Enterobacteriaceae	Raoultella	NA
SNOT22	ASV_53d_zfm	0,001708089	0,850612208	0,969510742	Bacteria	Actinobacteriota	Actinobacteria	Corynebacteriales	Corynebacteriaceae	Lawsonella	NA
SNOT22	ASV_53v_62f	-0,019029719	0,759395783	0,953699967	Bacteria	Firmicutes	Bacilli	Staphylococcales	Staphylococcaceae	Staphylococcus	NA
SNOT22	ASV_566_wbp	0,272460294	0,215606821	0,619097787	Bacteria	Actinobacteriota	Actinobacteria	Corynebacteriales	Corynebacteriaceae	Corynebacterium	NA
SNOT22	ASV_5do_e0k	-0,21315578	0,324240115	0,688666246	Bacteria	Actinobacteriota	Actinobacteria	Corynebacteriales	Corynebacteriaceae	Corynebacterium	NA
SNOT22	ASV_5j9_202	-0,115427856	0,521849108	0,871046551	Bacteria	Actinobacteriota	Actinobacteria	Corynebacteriales	Corynebacteriaceae	Corynebacterium	NA
SNOT22	ASV_5mh_1v6	0,029837109	0,62505595	0,910785708	Bacteria	Firmicutes	Bacilli	Staphylococcales	Staphylococcaceae	Staphylococcus	NA
SNOT22	ASV_5oo_dg6	0,00612734	0,546415317	0,870711553	Bacteria	Actinobacteriota	Actinobacteria	Micrococcales	Micrococcaceae	Rothia	mucilaginosa
SNOT22	ASV_5r4_ie2	-0,047187398	0,54646896	0,867173228	Bacteria	Actinobacteriota	Actinobacteria	Corynebacteriales	Corynebacteriaceae	Corynebacterium	NA
SNOT22	ASV_5rr_zcf	-0,06760279	0,500849683	0,84336227	Bacteria	Actinobacteriota	Actinobacteria	Corynebacteriales	Corynebacteriaceae	Corynebacterium	NA
SNOT22	ASV_5ry_ol3	-0,085239671	0,526215544	0,84301497	Bacteria	Actinobacteriota	Actinobacteria	Corynebacteriales	Corynebacteriaceae	Corynebacterium	NA
SNOT22	ASV_62i_805	-0,064402989	0,526971561	0,845621979	Bacteria	Proteobacteria	Gammaproteobacteria	Enterobacterales	Enterobacteriaceae	Pseudocitrobacter	NA
SNOT22	ASV_66n_hd0	0,119391425	0,528902539	0,875231269	Bacteria	Firmicutes	Bacilli	Staphylococcales	Staphylococcaceae	Staphylococcus	aureus
SNOT22	ASV_6dr_o2s	-0,035971614	0,545017492	0,863884972	Bacteria	Firmicutes	Bacilli	Staphylococcales	Staphylococcaceae	Staphylococcus	NA
SNOT22	ASV_6ut_tpw	-0,057609322	0,505934547	0,838842207	Bacteria	Proteobacteria	Gammaproteobacteria	Burkholderiales	Comamonadaceae	Tepidimonas	NA
SNOT22	ASV_6zj_cil	-0,106837256	0,463813018	0,819916719	Bacteria	Proteobacteria	Gammaproteobacteria	Pseudomonadales	Moraxellaceae	Moraxella	nonliquefaciens

Variable	FeatureID	spearman.erho	spearman.ep	spearman.eBH	Domain	Phylum	Class	Order	Family	Genus	Species
SNOT22	ASV_727_4zo	0,203774186	0,355516062	0,725678927	Bacteria	Firmicutes	Bacilli	Lactobacillales	Carnobacteriaceae	Dolosigranulum	NA
SNOT22	ASV_79g_5uy	-0,153269484	0,427549242	0,776338879	Bacteria	Firmicutes	Clostridia	Lachnospirales	Lachnospiraceae	Roseburia	NA
SNOT22	ASV_7cr_wom	0,067756117	0,592440135	0,880617043	Bacteria	Firmicutes	Bacilli	Staphylococcales	Staphylococcaceae	Staphylococcus	NA
SNOT22	ASV_7jt_wq3	-0,242726358	0,279122412	0,672027967	Bacteria	Actinobacteriota	Actinobacteria	Corynebacteriales	Corynebacteriaceae	Corynebacterium	NA
SNOT22	ASV_7ka_s2c	-0,007862656	0,546411555	0,869298553	Bacteria	Actinobacteriota	Actinobacteria	Corynebacteriales	Corynebacteriaceae	Corynebacterium	NA
SNOT22	ASV_7sw_rnc	-0,020755004	0,536725006	0,856899681	Bacteria	Proteobacteria	Gammaproteobacteria	Enterobacterales	Pasteurellaceae	Haemophilus	NA
SNOT22	ASV_7x9_tvb	0,213599998	0,245880047	0,767393773	Bacteria	Firmicutes	Clostridia	Peptostreptococcales-Tissierellales	Family XI	Peptoniphilus	gorbachii/lacydonensis/rhinitidis
SNOT22	ASV_873_9b5	0,087069562	0,527580781	0,859625355	Bacteria	Firmicutes	Bacilli	Staphylococcales	Staphylococcaceae	NA	NA
SNOT22	ASV_8fh_5b9	-0,091073255	0,518993797	0,823518255	Bacteria	Proteobacteria	Gammaproteobacteria	Enterobacterales	Pasteurellaceae	Haemophilus	NA
SNOT22	ASV_8gj_ney	-0,060707383	0,535327119	0,8585282	Bacteria	Firmicutes	Bacilli	Staphylococcales	Staphylococcaceae	Staphylococcus	NA
SNOT22	ASV_8lf_xek	-0,173336666	0,386521498	0,752180039	Bacteria	Bacteroidota	Bacteroidia	Bacteroidales	Prevotellaceae	Prevotella_9	copri
SNOT22	ASV_8lv_u9v	0,013785083	0,488855319	0,844423759	Bacteria	Firmicutes	Bacilli	Lactobacillales	Streptococcaceae	Streptococcus	NA
SNOT22	ASV_8pb_fin	-0,154483201	0,419099589	0,762812053	Bacteria	Bacteroidota	Bacteroidia	Cytophagales	Hymenobacteraceae	Hymenobacter	NA
SNOT22	ASV_8re_um4	0,12672102	0,493051696	0,851650171	Bacteria	Firmicutes	Bacilli	Staphylococcales	Staphylococcaceae	Staphylococcus	NA
SNOT22	ASV_8v2_sgp	-0,197853192	0,338812006	0,749217124	Bacteria	Actinobacteriota	Actinobacteria	Corynebacteriales	Corynebacteriaceae	Corynebacterium	NA
SNOT22	ASV_8zu_e97	0,15714421	0,430839715	0,795085196	Bacteria	Firmicutes	Bacilli	Staphylococcales	Staphylococcaceae	Staphylococcus	NA
SNOT22	ASV_926_7zf	0,045926393	0,673460568	0,928459459	Bacteria	Firmicutes	Bacilli	Staphylococcales	Staphylococcaceae	Staphylococcus	NA
SNOT22	ASV_98z_21y	-0,148520652	0,42377987	0,784539588	Bacteria	Proteobacteria	Gammaproteobacteria	Pseudomonadales	Pseudomonadaceae	Pseudomonas	taetrolens
SNOT22	ASV_99w_90h	-0,076555929	0,516883256	0,846377145	Bacteria	Proteobacteria	Gammaproteobacteria	Enterobacterales	Enterobacteriaceae	NA	NA
SNOT22	ASV_9cd_tui	-0,159295084	0,420427269	0,777634782	Bacteria	Firmicutes	Bacilli	Lactobacillales	Enterococcaceae	Tetragenococcus	NA
SNOT22	ASV_9d0_o06	0,067250282	0,503403245	0,844334325	Bacteria	Firmicutes	Negativicutes	Veillonellales-Selenomonadales	Veillonellaceae	Negativicoccus	NA
SNOT22	ASV_9eq_kr6	0,03146352	0,51397629	0,844820239	Bacteria	Firmicutes	Bacilli	Lactobacillales	Streptococcaceae	Streptococcus	mitis/parasanguinis
SNOT22	ASV_9ho_7z1	0,02287292	0,55260267	0,859056112	Bacteria	Actinobacteriota	Actinobacteria	Actinomycetales	Actinomycetaceae	NA	NA
SNOT22	ASV_9jb_2ek	-0,172177401	0,394377792	0,796548555	Bacteria	Actinobacteriota	Actinobacteria	Corynebacteriales	Corynebacteriaceae	Corynebacterium	NA
SNOT22	ASV_9kg_f3f	-0,122604123	0,464902221	0,801241158	Bacteria	Actinobacteriota	Actinobacteria	Corynebacteriales	NA	NA	NA
SNOT22	ASV_9l0_247	0,25982158	0,250993668	0,643300931	Bacteria	Firmicutes	Bacilli	Staphylococcales	Gemellaceae	Gemella	morbillorum
SNOT22	ASV_9mx_kho	-0,044029153	0,525217513	0,856316739	Bacteria	Actinobacteriota	Actinobacteria	Frankiales	Sporichthyaceae	NA	NA
SNOT22	ASV_9o6_1ja	0,155354442	0,425507631	0,84269766	Bacteria	Firmicutes	Bacilli	Staphylococcales	Staphylococcaceae	Staphylococcus	aureus
SNOT22	ASV_9t2_1wo	-0,109691714	0,448081459	0,809662369	Bacteria	Proteobacteria	Gammaproteobacteria	Pseudomonadales	Moraxellaceae	Acinetobacter	NA
SNOT22	ASV_9vy_jj2	0,066390505	0,520722288	0,870495469	Bacteria	Firmicutes	Bacilli	Staphylococcales	Staphylococcaceae	Staphylococcus	NA
SNOT22	ASV_9wz_9u7	0,144041216	0,434674649	0,78788112	Bacteria	Actinobacteriota	Actinobacteria	NA	NA	NA	NA
SNOT22	ASV_a5h_c27	-0,1115388	0,482784513	0,819867542	Bacteria	Firmicutes	Bacilli	Lactobacillales	Lactobacillaceae	Lacticaseibacillus	NA

Variable	FeatureID	spearman.erho	spearman.ep	spearman.eBH	Domain	Phylum	Class	Order	Family	Genus	Species
SNOT22	ASV_aew_fyi	-0,288040877	0,199815211	0,602936534	Bacteria	Actinobacteriota	Actinobacteria	Corynebacteriales	Corynebacteriaceae	Corynebacterium	mucifaciens
SNOT22	ASV_ajo_fen	0,393095529	0,053566225	0,403019819	Bacteria	Actinobacteriota	Actinobacteria	Corynebacteriales	Corynebacteriaceae	Corynebacterium	NA
SNOT22	ASV_aqj_vcn	0,09057745	0,564007573	0,883736382	Bacteria	Firmicutes	Bacilli	Lactobacillales	Streptococcaceae	Streptococcus	mitis/phage/pneumoniae/pseudopneumoniae
SNOT22	ASV_arl_xwg	-0,150204381	0,42020634	0,774694451	Bacteria	Firmicutes	Clostridia	Lachnospirales	Lachnospiraceae	Blautia	NA
SNOT22	ASV_aw8_q9 6	0,016728384	0,662117417	0,927339542	Bacteria	Actinobacteriota	Actinobacteria	Corynebacteriales	Corynebacteriaceae	Corynebacterium	NA
SNOT22	ASV_b6a_uff	-0,105453016	0,443432351	0,806660544	Bacteria	Bacteroidota	Bacteroidia	Sphingobacteriales	Sphingobacteriaceae	Sphingobacterium	NA
SNOT22	ASV_bg2_zbh	-0,139223602	0,439915887	0,784316773	Bacteria	Firmicutes	Bacilli	Staphylococcales	Staphylococcaceae	Staphylococcus	NA
SNOT22	ASV_bh3_94k	-0,148645319	0,408903387	0,756762814	Bacteria	Firmicutes	Bacilli	Lactobacillales	Streptococcaceae	Lactococcus	NA
SNOT22	ASV_bnk_uhh	-0,16904065	0,409580872	0,803930025	Bacteria	Firmicutes	Clostridia	Peptostreptococcales-Tissierellales	Family XI	Anaerococcus	provencensis
SNOT22	ASV_btl_vxl	-0,234896661	0,264731961	0,69032054	Bacteria	Firmicutes	Clostridia	Peptostreptococcales-Tissierellales	Family XI	Anaerococcus	vaginalis
SNOT22	ASV_bwx_we f	-0,119709542	0,486006929	0,83670242	Bacteria	Actinobacteriota	Actinobacteria	Corynebacteriales	Corynebacteriaceae	Corynebacterium	amycolatum/jeikeium/lactis/urealyticum/vitaeruminis
SNOT22	ASV_c46_jpb	-0,156111045	0,423434809	0,791780446	Bacteria	Actinobacteriota	Actinobacteria	Corynebacteriales	Corynebacteriaceae	Corynebacterium	NA
SNOT22	ASV_c4o_xm s	-0,015044655	0,680660347	0,932594508	Bacteria	Firmicutes	Bacilli	Staphylococcales	Staphylococcaceae	NA	NA
SNOT22	ASV_cep_rux	-0,205371937	0,332594116	0,737845441	Bacteria	Firmicutes	Clostridia	Peptostreptococcales-Tissierellales	Family XI	Anaerococcus	NA
SNOT22	ASV_cje_lx4	0,055954252	0,532950046	0,858547387	Bacteria	Proteobacteria	Gammaproteobacteria	Burkholderiales	Neisseriaceae	NA	NA
SNOT22	ASV_co0_sbi	0,058906151	0,533346693	0,860365391	Bacteria	Actinobacteriota	Actinobacteria	Corynebacteriales	Corynebacteriaceae	Corynebacterium	NA
SNOT22	ASV_cqp_otd	-0,139388393	0,437674533	0,801935966	Bacteria	Firmicutes	Negativicutes	Veillonellales-Selenomonadales	Veillonellaceae	Negativicoccus	NA
SNOT22	ASV_cy2_em p	-0,017027873	0,499689342	0,844581547	Bacteria	Actinobacteriota	Actinobacteria	Corynebacteriales	Corynebacteriaceae	Corynebacterium	NA
SNOT22	ASV_czd_a30	-0,073462167	0,529532085	0,847958087	Bacteria	Firmicutes	Bacilli	Staphylococcales	Staphylococcaceae	Staphylococcus	NA
SNOT22	ASV_d0q_oe1	-0,172949767	0,374768144	0,741725622	Bacteria	Bacteroidota	Bacteroidia	Bacteroidales	Prevotellaceae	Prevotella_9	NA
SNOT22	ASV_da2_u30	-0,036584921	0,543263203	0,87661723	Bacteria	Bacteroidota	Bacteroidia	Bacteroidales	Prevotellaceae	Prevotella	buccalis
SNOT22	ASV_dhe_20n	0,048254954	0,722080783	0,942085507	Bacteria	Firmicutes	Bacilli	Staphylococcales	Staphylococcaceae	Staphylococcus	aureus
SNOT22	ASV_dlu_bk7	-0,066391938	0,620532122	0,903748166	Bacteria	Proteobacteria	Gammaproteobacteria	Burkholderiales	Neisseriaceae	NA	NA
SNOT22	ASV_dmt_o1x	0,067731757	0,584444391	0,884191441	Bacteria	Proteobacteria	Gammaproteobacteria	Enterobacterales	Pasteurellaceae	Haemophilus	haemolyticus/influenzae
SNOT22	ASV_dui_4x1	0,214188945	0,323401126	0,711043541	Bacteria	Firmicutes	Bacilli	Lactobacillales	Carnobacteriaceae	Dolosigranulum	NA
SNOT22	ASV_dz0_fy2	0,315960684	0,148546065	0,554848483	Bacteria	Actinobacteriota	Actinobacteria	Corynebacteriales	Corynebacteriaceae	Corynebacterium	NA
SNOT22	ASV_e7g_uqv	-0,011169929	0,576625714	0,875141279	Bacteria	Actinobacteriota	Thermoleophilia	Gaiellales	NA	NA	NA
SNOT22	ASV_e8x_8cs	0,273792948	0,158214568	0,642227603	Bacteria	Actinobacteriota	Actinobacteria	Corynebacteriales	Corynebacteriaceae	Lawsonella	NA
SNOT22	ASV_egx_g4e	-0,09918238	0,496718414	0,823702609	Bacteria	Proteobacteria	Gammaproteobacteria	Enterobacterales	NA	NA	NA
SNOT22	ASV_elz_ffm	0,009233999	0,545176698	0,871149952	Bacteria	Actinobacteriota	Actinobacteria	Corynebacteriales	Corynebacteriaceae	Corynebacterium	NA
SNOT22	ASV_f1j_s2e	-0,122134112	0,517470253	0,859761939	Bacteria	Actinobacteriota	Actinobacteria	Corynebacteriales	Corynebacteriaceae	Corynebacterium	aurimucosum/simulans/striatum/xerosis
SNOT22	ASV_f6c_v0m	-0,117247716	0,482859453	0,824326514	Bacteria	Actinobacteriota	Actinobacteria	Corynebacteriales	Corynebacteriaceae	Corynebacterium	NA

Variable	FeatureID	spearman.erho	spearman.ep	spearman.eBH	Domain	Phylum	Class	Order	Family	Genus	Species
SNOT22	ASV_f8c_ir4	-0,17364332	0,356109571	0,745408937	Bacteria	Bacteroidota	Bacteroidia	Bacteroidales	Bacteroidaceae	Bacteroides	plebeius
SNOT22	ASV_fb6_dje	-0,021379775	0,534641729	0,858665317	Bacteria	Firmicutes	Bacilli	Staphylococcales	Staphylococcaceae	Staphylococcus	aureus/devriesei/epidermidis/haemolyticus/hominis
SNOT22	ASV_fe7_hpm	-0,044304281	0,56224609	0,872169881	Bacteria	Actinobacteriota	Actinobacteria	Corynebacteriales	Corynebacteriaceae	Corynebacterium	NA
SNOT22	ASV_ffj_wqa	-0,086056459	0,484715763	0,830883791	Bacteria	Proteobacteria	Gammaproteobacteria	Pseudomonadales	Moraxellaceae	Acinetobacter	NA
SNOT22	ASV_fgq_ohv	0,238060638	0,296945698	0,673120894	Bacteria	Actinobacteriota	Actinobacteria	Corynebacteriales	Corynebacteriaceae	Corynebacterium	NA
SNOT22	ASV_fmb_me n	-0,053074001	0,537595463	0,868170511	Bacteria	Actinobacteriota	Actinobacteria	Corynebacteriales	Corynebacteriaceae	Corynebacterium	NA
SNOT22	ASV_fqm_8p m	-0,043540513	0,643862451	0,919984496	Bacteria	Firmicutes	Bacilli	Lactobacillales	Streptococcaceae	Streptococcus	anginosus/cristatus/infantis/mitis/oralis/pneumoniae/pseudopneumoniae/sanguinis/timon ensis
SNOT22	ASV_fug_ldj	-0,012624384	0,562226812	0,880203888	Bacteria	Bacteroidota	Bacteroidia	Bacteroidales	Prevotellaceae	Prevotella_9	NA
SNOT22	ASV_g3d_cs4	-0,107886183	0,4668354	0,824590676	Bacteria	Proteobacteria	Gammaproteobacteria	Enterobacterales	Pasteurellaceae	Haemophilus	influenzae
SNOT22	ASV_geq_907	-0,069928485	0,701127588	0,943775692	Bacteria	Firmicutes	Clostridia	Peptostreptococcales-Tissierellales	Family XI	Anaerococcus	octavius
SNOT22	ASV_gfz_fyn	-0,077521745	0,457839846	0,828287351	Bacteria	Proteobacteria	Gammaproteobacteria	Enterobacterales	Enterobacteriaceae	NA	NA
SNOT22	ASV_gg8_mtq	-0,047312066	0,530034651	0,86904821	Bacteria	Bacteroidota	Bacteroidia	Bacteroidales	Muribaculaceae	NA	NA
SNOT22	ASV_gzb_mis	-0,177467892	0,376711075	0,749525757	Bacteria	Firmicutes	Clostridia	Lachnospirales	Lachnospiraceae	Coprococcus	catus
SNOT22	ASV_h1r_zyt	0,022134945	0,554770257	0,870015115	Bacteria	Fusobacteriota	Fusobacteriia	Fusobacteriales	Fusobacteriaceae	Fusobacterium	NA
SNOT22	ASV_h40_0m d	0,286632276	0,205428063	0,599294158	Bacteria	Actinobacteriota	Actinobacteria	Corynebacteriales	Corynebacteriaceae	Corynebacterium	NA
SNOT22	ASV_h4w_6w i	-0,107198362	0,452698998	0,803451991	Bacteria	Firmicutes	Bacilli	Staphylococcales	Staphylococcaceae	NA	NA
SNOT22	ASV_h8v_sx4	-0,093095162	0,465465853	0,809746561	Bacteria	Actinobacteriota	Actinobacteria	Corynebacteriales	Nocardiaceae	Millisia	NA
SNOT22	ASV_hbi_ebs	-0,056014436	0,538704965	0,85917788	Bacteria	Actinobacteriota	Actinobacteria	Corynebacteriales	Corynebacteriaceae	Lawsonella	clevelandensis
SNOT22	ASV_het_bu3	0,019469638	0,583471069	0,887942795	Bacteria	Campylobacterota	Campylobacteria	Campylobacterales	Campylobacteraceae	Campylobacter	NA
SNOT22	ASV_hjn_92c	0,212321797	0,261053663	0,763135195	Bacteria	Firmicutes	Clostridia	Peptostreptococcales-Tissierellales	Family XI	Finegoldia	magna
SNOT22	ASV_hkw_6s 3	-0,011380574	0,544125311	0,867085624	Bacteria	Actinobacteriota	Actinobacteria	Corynebacteriales	Corynebacteriaceae	Corynebacterium	NA
SNOT22	ASV_hpm_svr	0,332154574	0,118840057	0,522200383	Bacteria	Actinobacteriota	Actinobacteria	Corynebacteriales	Corynebacteriaceae	Corynebacterium	NA
SNOT22	ASV_hq8_93x	0,30900366	0,174831827	0,556170971	Bacteria	Actinobacteriota	Actinobacteria	Corynebacteriales	Corynebacteriaceae	Corynebacterium	NA
SNOT22	ASV_i0i_mxo	-0,021601884	0,582772084	0,893207297	Bacteria	Actinobacteriota	Actinobacteria	Corynebacteriales	Corynebacteriaceae	Corynebacterium	pyruviciproducens
SNOT22	ASV_i35_0op	0,434459375	0,029532011	0,331256492	Bacteria	Actinobacteriota	Actinobacteria	Corynebacteriales	Corynebacteriaceae	Corynebacterium	aurimucosum/jeikeium/propinquum/pseudodiphtheriticum
SNOT22	ASV_i88_mm 0	-0,019405155	0,533992374	0,85877621	Bacteria	Actinobacteriota	Actinobacteria	Corynebacteriales	Corynebacteriaceae	Corynebacterium	NA
SNOT22	ASV_ic5_nai	-0,072814468	0,51133922	0,841229905	Bacteria	Firmicutes	Bacilli	Lactobacillales	Streptococcaceae	Streptococcus	sobrinus
SNOT22	ASV_igm_m4 9	-0,004245863	0,5203683	0,858835517	Bacteria	NA	NA	NA	NA	NA	NA
SNOT22	ASV_ii5_tjz	0,159892628	0,422169247	0,794689444	Bacteria	Firmicutes	Bacilli	Staphylococcales	Staphylococcaceae	Staphylococcus	NA
SNOT22	ASV_ikg_ra0	0,422421072	0,040475242	0,344969907	Bacteria	Actinobacteriota	Actinobacteria	Corynebacteriales	Corynebacteriaceae	Corynebacterium	NA
SNOT22	ASV_iov_l70	0,228780784	0,294260999	0,703740299	Bacteria	Firmicutes	Bacilli	Lactobacillales	Carnobacteriaceae	Dolosigranulum	pigrum

Variable	FeatureID	spearman.erho	spearman.ep	spearman.eBH	Domain	Phylum	Class	Order	Family	Genus	Species
SNOT22	ASV_iqo_ea7	-0,103584435	0,443101667	0,813741175	Bacteria	Firmicutes	Bacilli	Staphylococcales	Staphylococcaceae	Staphylococcus	NA
SNOT22	ASV_jjw_h6c	-0,17505192	0,343520655	0,828800494	Bacteria	Actinobacteriota	Actinobacteria	Corynebacteriales	Corynebacteriaceae	Corynebacterium	NA
SNOT22	ASV_jl5_6pn	0,069051513	0,627477645	0,908337883	Bacteria	Firmicutes	Bacilli	Staphylococcales	Staphylococcaceae	Staphylococcus	aureus
SNOT22	ASV_jp2_wik	-0,094422084	0,618747297	0,918010866	Bacteria	Firmicutes	Bacilli	Staphylococcales	Staphylococcaceae	Staphylococcus	epidermidis
SNOT22	ASV_jph_cup	0,405212646	0,039931565	0,379042309	Bacteria	Actinobacteriota	Actinobacteria	Corynebacteriales	Corynebacteriaceae	Corynebacterium	NA
SNOT22	ASV_jy3_f61	-0,046836323	0,52216166	0,86098489	Bacteria	Actinobacteriota	Actinobacteria	Corynebacteriales	Corynebacteriaceae	Corynebacterium	NA
SNOT22	ASV_jy9_jqd	-0,138329435	0,40605804	0,791302423	Bacteria	Firmicutes	Clostridia	Lachnospirales	Lachnospiraceae	[Eubacterium] ventriosum group	NA
SNOT22	ASV_k2s_cqm	0,276557129	0,207546268	0,616992429	Bacteria	Actinobacteriota	Actinobacteria	Corynebacteriales	Corynebacteriaceae	Corynebacterium	NA
SNOT22	ASV_k3h_3z5	-0,153154847	0,413901794	0,780768216	Bacteria	Proteobacteria	Gammaproteobacteria	Xanthomonadales	Xanthomonadaceae	Lysobacter	NA
SNOT22	ASV_k3h_qcz	0,081225948	0,525299234	0,849872235	Bacteria	Firmicutes	Bacilli	Staphylococcales	Staphylococcaceae	Staphylococcus	NA
SNOT22	ASV_kaj_b2n	-0,08748942	0,446402616	0,8046225	Bacteria	Firmicutes	Bacilli	Lactobacillales	P5D1-392	NA	NA
SNOT22	ASV_kcs_cbi	0,223656517	0,303696084	0,693918249	Bacteria	Actinobacteriota	Actinobacteria	Corynebacteriales	Corynebacteriaceae	Corynebacterium	NA
SNOT22	ASV_kdo_fhg	-0,128060838	0,446935033	0,812644201	Bacteria	Proteobacteria	Gammaproteobacteria	Burkholderiales	Comamonadaceae	Mitsuaria	NA
SNOT22	ASV_ki3_dfk	0,325253435	0,148052297	0,530837295	Bacteria	Actinobacteriota	Actinobacteria	Corynebacteriales	Corynebacteriaceae	Corynebacterium	NA
SNOT22	ASV_kkl_mxy	0,024922054	0,655583416	0,924403915	Bacteria	Actinobacteriota	Actinobacteria	Corynebacteriales	Corynebacteriaceae	Corynebacterium	kroppenstedtii
SNOT22	ASV_kru_fff	0,008043209	0,545731026	0,869444691	Bacteria	Proteobacteria	Gammaproteobacteria	Enterobacterales	Erwiniaceae	NA	NA
SNOT22	ASV_ktj_lyl	-0,024417651	0,553068173	0,865528218	Bacteria	Proteobacteria	Gammaproteobacteria	Burkholderiales	Neisseriaceae	NA	NA
SNOT22	ASV_l3n_w4d	-0,001748212	0,563842929	0,875441449	Bacteria	Synergistota	Synergistia	Synergistales	Synergistaceae	Jonquetella	anthropi
SNOT22	ASV_lky_mqj	0,013183239	0,572847952	0,8754203	Bacteria	Firmicutes	Bacilli	Lactobacillales	Streptococcaceae	Streptococcus	NA
SNOT22	ASV_lmq_jff	0,213025381	0,324404369	0,722474114	Bacteria	Firmicutes	Bacilli	Lactobacillales	Carnobacteriaceae	Dolosigranulum	NA
SNOT22	ASV_lnh_9fs	-0,296046829	0,17824854	0,58674834	Bacteria	Actinobacteriota	Actinobacteria	Corynebacteriales	Corynebacteriaceae	Corynebacterium	afermentans/coyleae
SNOT22	ASV_lvd_sr9	0,072536474	0,623369352	0,903009998	Bacteria	Actinobacteriota	Actinobacteria	Corynebacteriales	Corynebacteriaceae	Corynebacterium	NA
SNOT22	ASV_lvh_wn0	-0,062567366	0,585538915	0,894841655	Bacteria	Actinobacteriota	Actinobacteria	Micrococcales	Dermabacteraceae	Dermabacter	jinjuensis
SNOT22	ASV_m52_gpv	-0,025705883	0,521980135	0,858164724	Bacteria	Actinobacteriota	Actinobacteria	Corynebacteriales	Corynebacteriaceae	Corynebacterium	NA
SNOT22	ASV_mbn_3b6	-0,165346477	0,385362734	0,77676345	Bacteria	Firmicutes	Clostridia	Lachnospirales	Lachnospiraceae	[Ruminococcus] torques group	NA
SNOT22	ASV_mc2_eno	-0,121032165	0,454372464	0,814978547	Bacteria	Proteobacteria	Gammaproteobacteria	Xanthomonadales	Xanthomonadaceae	Lysobacter	NA
SNOT22	ASV_mcc_ype	0,106421697	0,52545448	0,860506934	Bacteria	Firmicutes	Bacilli	Staphylococcales	Staphylococcaceae	Staphylococcus	epidermidis
SNOT22	ASV_mgr_u9o	0,304582976	0,179098263	0,566914321	Bacteria	Actinobacteriota	Actinobacteria	Corynebacteriales	Corynebacteriaceae	Corynebacterium	NA
SNOT22	ASV_miq_ryo	-0,077465859	0,490644631	0,827028916	Bacteria	Firmicutes	Bacilli	Staphylococcales	Staphylococcaceae	Staphylococcus	NA
SNOT22	ASV_mlb_2sd	0,09626917	0,544016509	0,858242034	Bacteria	Firmicutes	Bacilli	Staphylococcales	Staphylococcaceae	Staphylococcus	NA
SNOT22	ASV_mm9_js5	0,044987803	0,643800803	0,911710159	Bacteria	Firmicutes	Bacilli	Staphylococcales	Staphylococcaceae	Staphylococcus	aureus
SNOT22	ASV_mvn_oem	0,019132892	0,573250491	0,888415339	Bacteria	Actinobacteriota	Actinobacteria	Corynebacteriales	Corynebacteriaceae	Corynebacterium	NA

Variable	FeatureID	spearman.erho	spearman.ep	spearman.eBH	Domain	Phylum	Class	Order	Family	Genus	Species
SNOT22	ASV_mx9_gyi	-0,089836609	0,495980845	0,835642454	Bacteria	Bacteroidota	Bacteroidia	Bacteroidales	Bacteroidaceae	Bacteroides	uniformis
SNOT22	ASV_n1a_pw6	-0,02592226	0,543043978	0,870240298	Bacteria	Proteobacteria	Alphaproteobacteria	Rhizobiales	Methyloligellaceae	NA	NA
SNOT22	ASV_n5f_h6y	-0,084430048	0,4812866	0,824060861	Bacteria	Proteobacteria	Gammaproteobacteria	Burkholderiales	Oxalobacteraceae	Massilia	NA
SNOT22	ASV_n5j_9sq	0,073771686	0,501017062	0,84553182	Bacteria	Firmicutes	Bacilli	Staphylococcales	Staphylococcaceae	Staphylococcus	NA
SNOT22	ASV_n79_65k	0,026900972	0,548809747	0,880878263	Bacteria	Proteobacteria	Gammaproteobacteria	Burkholderiales	Neisseriaceae	NA	NA
SNOT22	ASV_nbq_6pg	-0,071272603	0,50942331	0,841899226	Bacteria	Firmicutes	Clostridia	Peptostreptococcales-Tissierellales	Family XI	Anaerococcus	prevotii/tetradius
SNOT22	ASV_nk5_hwz	-0,093397517	0,498994239	0,837665996	Bacteria	Bacteroidota	Bacteroidia	Flavobacteriales	Weeksellaceae	Cloacibacterium	normanense
SNOT22	ASV_ntr_hh5	-0,180381101	0,340458749	0,723092471	Bacteria	Proteobacteria	Gammaproteobacteria	Enterobacterales	Pasteurellaceae	Haemophilus	haemolyticus/influenzae/parainfluenzae
SNOT22	ASV_nvc_oao	0,049683616	0,655665176	0,923810612	Bacteria	Deinococcota	Deinococci	Thermales	Thermaceae	Thermus	parvatiensis/thermophilus
SNOT22	ASV_nvm_lyw	-0,130822153	0,447798167	0,79239994	Bacteria	Firmicutes	Clostridia	Oscillospirales	Oscillospiraceae	Intestinimonas	NA
SNOT22	ASV_o0r_len	0,00698855	0,702762232	0,937471029	Bacteria	Firmicutes	Bacilli	Staphylococcales	Staphylococcaceae	Staphylococcus	aureus/capitis/caprae/epidermidis/haemolyticus/saprophyticus/warneri
SNOT22	ASV_o10_2p1	-0,195776832	0,349654712	0,727078426	Bacteria	Proteobacteria	Gammaproteobacteria	Pseudomonadales	Pseudomonadaceae	Pseudomonas	brassicacearum/chlororaphis/corrugata/fluorescens/jessenii/kilonensis/mohnii/putida/synxantha
SNOT22	ASV_o5q_r99	-0,10815558	0,449865227	0,796124838	Bacteria	Actinobacteriota	Actinobacteria	Corynebacteriales	Corynebacteriaceae	Corynebacterium	NA
SNOT22	ASV_ode_i48	-0,120632369	0,422471768	0,778781775	Bacteria	Actinobacteriota	Actinobacteria	Corynebacteriales	Corynebacteriaceae	Corynebacterium	NA
SNOT22	ASV_on0_uxs	-0,48938906	0,014078256	0,22656209	Bacteria	Actinobacteriota	Actinobacteria	Corynebacteriales	Corynebacteriaceae	Corynebacterium	afermentans/ihumii
SNOT22	ASV_onw_luq	0,037923307	0,54702421	0,870130538	Bacteria	Proteobacteria	Gammaproteobacteria	Burkholderiales	Neisseriaceae	Eikenella	NA
SNOT22	ASV_ouf_s4v	-0,115240138	0,475022529	0,822745009	Bacteria	Actinobacteriota	Actinobacteria	Corynebacteriales	Corynebacteriaceae	Corynebacterium	NA
SNOT22	ASV_oum_efh	-0,085235372	0,448497978	0,813764706	Bacteria	Firmicutes	Bacilli	Staphylococcales	Staphylococcaceae	Staphylococcus	NA
SNOT22	ASV_oxw_74i	-0,11080799	0,470583519	0,817614895	Bacteria	Proteobacteria	Alphaproteobacteria	Acetobacterales	Acetobacteraceae	Roseomonas	NA
SNOT22	ASV_oyd_63r	0,101284533	0,554174222	0,861806543	Bacteria	Firmicutes	Bacilli	Staphylococcales	Staphylococcaceae	Staphylococcus	NA
SNOT22	ASV_p3d_k08	-0,219730204	0,311587316	0,71027604	Bacteria	Firmicutes	Clostridia	Peptostreptococcales-Tissierellales	Family XI	W5053	NA
SNOT22	ASV_p46_336	-0,066506575	0,518697234	0,85924368	Bacteria	Proteobacteria	Gammaproteobacteria	Enterobacterales	Enterobacteriaceae	Escherichia-Shigella	NA
SNOT22	ASV_p9u_72k	-0,145153194	0,409904348	0,765380637	Bacteria	Firmicutes	Clostridia	Peptococcales	Peptococcaceae	Peptococcus	NA
SNOT22	ASV_paz_uyl	-0,134175281	0,478875649	0,866181634	Bacteria	Actinobacteriota	Actinobacteria	Propionibacteriales	Propionibacteriaceae	Cutibacterium	acnes/avidum
SNOT22	ASV_pgj_6t5	-0,128496458	0,443507816	0,792325216	Bacteria	Actinobacteriota	Actinobacteria	Corynebacteriales	Corynebacteriaceae	Corynebacterium	NA
SNOT22	ASV_pjp_yzq	-0,016814362	0,545529372	0,869723917	Bacteria	Firmicutes	Bacilli	Staphylococcales	Staphylococcaceae	Staphylococcus	NA
SNOT22	ASV_pni_ryk	-0,061737682	0,528512066	0,857016082	Bacteria	Actinobacteriota	Actinobacteria	Corynebacteriales	Corynebacteriaceae	Corynebacterium	NA
SNOT22	ASV_pou_qqz	-0,106837256	0,458021678	0,818418658	Bacteria	Actinobacteriota	Actinobacteria	Micrococcales	Dermacoccaceae	Barrientosiimonas	humi/marina
SNOT22	ASV_pwy_pjt	0,261409301	0,234486872	0,639510629	Bacteria	Actinobacteriota	Actinobacteria	Corynebacteriales	Corynebacteriaceae	Corynebacterium	NA
SNOT22	ASV_q1f_0em	0,291528703	0,192516158	0,585892444	Bacteria	Actinobacteriota	Actinobacteria	Corynebacteriales	Corynebacteriaceae	Corynebacterium	NA
SNOT22	ASV_q1k_ad2	0,166769407	0,398722213	0,797956016	Bacteria	Firmicutes	Bacilli	Staphylococcales	Staphylococcaceae	Staphylococcus	NA
SNOT22	ASV_q1o_24k	-0,304979906	0,092488488	0,586095177	Bacteria	Actinobacteriota	Actinobacteria	Corynebacteriales	Corynebacteriaceae	Corynebacterium	accolens/fastidiosum

Variable	FeatureID	spearman.erho	spearman.ep	spearman.eBH	Domain	Phylum	Class	Order	Family	Genus	Species
SNOT22	ASV_q1r_s4c	-0,029289718	0,529227187	0,860438874	Bacteria	Actinobacteriota	Actinobacteria	Actinomycetales	Actinomycetaceae	Actinotignum	schaalii/timonense
SNOT22	ASV_qc1_pgs	-0,11283563	0,442370831	0,804518337	Bacteria	Firmicutes	Bacilli	Staphylococcales	Staphylococcaceae	Staphylococcus	NA
SNOT22	ASV_qe8_76v	-0,129134125	0,407980182	0,784449775	Bacteria	Proteobacteria	Gammaproteobacteria	Enterobacterales	Pasteurellaceae	Haemophilus	influenzae
SNOT22	ASV_qd8_nzn	-0,133195136	0,42430632	0,783960254	Bacteria	Actinobacteriota	Actinobacteria	Corynebacteriales	Corynebacteriaceae	Corynebacterium	jeikeium
SNOT22	ASV_qft_24l	0,006471251	0,581510881	0,880712542	Bacteria	Bacteroidota	Bacteroidia	Bacteroidales	Prevotellaceae	Alloprevotella	NA
SNOT22	ASV_qis_xxl	-0,082309266	0,529354354	0,8678276	Bacteria	Firmicutes	Clostridia	Peptostreptococcales-Tissierellales	Family XI	Anaerococcus	nagyae
SNOT22	ASV_qk1_qy8	-0,04193703	0,482802941	0,81797096	Bacteria	Proteobacteria	Gammaproteobacteria	Enterobacterales	Pasteurellaceae	Lonepinella	NA
SNOT22	ASV_r2m_1o3	-0,102505416	0,434898645	0,795065286	Bacteria	Firmicutes	Bacilli	Staphylococcales	Staphylococcaceae	Staphylococcus	NA
SNOT22	ASV_r73_72u	-0,020338012	0,554808998	0,868380835	Bacteria	Actinobacteriota	Actinobacteria	Corynebacteriales	Corynebacteriaceae	Corynebacterium	NA
SNOT22	ASV_r8p_k1e	0,080116836	0,598408379	0,904072424	Bacteria	Firmicutes	Bacilli	Staphylococcales	Staphylococcaceae	Staphylococcus	aureus
SNOT22	ASV_r9n_48y	-0,144125761	0,413655728	0,799322487	Bacteria	Firmicutes	Bacilli	Staphylococcales	Staphylococcaceae	Staphylococcus	NA
SNOT22	ASV_raj_rv1	0,041209086	0,816999157	0,968410231	Bacteria	Firmicutes	Bacilli	Staphylococcales	Staphylococcaceae	Staphylococcus	aureus/capitis/caprae/epidermidis/haemolyticus/warneri
SNOT22	ASV_rat_2jx	-0,129579776	0,436812198	0,784248863	Bacteria	Proteobacteria	Gammaproteobacteria	Enterobacterales	Enterobacteriaceae	Escherichia-Shigella	NA
SNOT22	ASV_rmx_t9x	-0,039608469	0,645417028	0,914353331	Bacteria	Firmicutes	Bacilli	Staphylococcales	Staphylococcaceae	Staphylococcus	aureus
SNOT22	ASV_rs1_k0s	0,162649645	0,402874017	0,771299552	Bacteria	Firmicutes	Bacilli	Staphylococcales	Staphylococcaceae	Staphylococcus	NA
SNOT22	ASV_rtx_tpz	-0,113476163	0,446995153	0,805725315	Bacteria	Proteobacteria	Gammaproteobacteria	Pseudomonadales	Moraxellaceae	Moraxella	catarrhalis/nonliquefaciens
SNOT22	ASV_s8r_baf	-0,01780597	0,688256979	0,931793422	Bacteria	Firmicutes	Clostridia	Peptostreptococcales-Tissierellales	Family XI	Anaerococcus	NA
SNOT22	ASV_sjv_qdw	-0,232684169	0,29783851	0,682350172	Bacteria	Actinobacteriota	Actinobacteria	Corynebacteriales	Corynebacteriaceae	Corynebacterium	NA
SNOT22	ASV_snw_j03	-0,164640027	0,386096501	0,766169075	Bacteria	Firmicutes	Bacilli	Lactobacillales	Streptococcaceae	Streptococcus	NA
SNOT22	ASV_ssr_vha	-0,01803811	0,690375009	0,933153325	Bacteria	Firmicutes	Bacilli	Staphylococcales	Staphylococcaceae	Staphylococcus	aureus
SNOT22	ASV_sza_hre	-0,0570519	0,520722061	0,834587447	Bacteria	Actinobacteriota	Actinobacteria	Corynebacteriales	Corynebacteriaceae	NA	NA
SNOT22	ASV_szb_rz6	-0,150926593	0,387169765	0,761775286	Bacteria	Firmicutes	Bacilli	Lactobacillales	Aerococcaceae	Facklamia	languida
SNOT22	ASV_t7p_n06	-0,172107186	0,398897901	0,783098761	Bacteria	Firmicutes	Clostridia	Peptostreptococcales-Tissierellales	Family XI	Anaerococcus	NA
SNOT22	ASV_t8l_ysz	0,133289712	0,479594342	0,849757313	Bacteria	Firmicutes	Bacilli	Staphylococcales	Staphylococcaceae	NA	NA
SNOT22	ASV_ten_mjo	-0,049503063	0,523878323	0,849458353	Bacteria	Actinobacteriota	Actinobacteria	Corynebacteriales	Corynebacteriaceae	Corynebacterium	NA
SNOT22	ASV_tu2_xgy	0,417600592	0,049567065	0,364967599	Bacteria	Actinobacteriota	Actinobacteria	Corynebacteriales	Corynebacteriaceae	Corynebacterium	NA
SNOT22	ASV_tvh_ar7	-0,023258386	0,557993238	0,856482248	Bacteria	Firmicutes	Bacilli	Lactobacillales	Streptococcaceae	Streptococcus	anginosus/constellatus/intermedius/lutetiensis
SNOT22	ASV_xex_kso	-0,142887683	0,462180946	0,790334618	Bacteria	Actinobacteriota	Actinobacteria	Micrococcales	Bogoriellaceae	Georgenia	NA
UPSIT.score	ASV_17p_uuy	0,011868826	0,746171359	0,960282774	Bacteria	Actinobacteriota	Actinobacteria	Corynebacteriales	Corynebacteriaceae	Corynebacterium	NA
UPSIT.score	ASV_19p_rjx	0,190293302	0,432296922	0,858800768	Bacteria	Bacteroidota	Bacteroidia	Bacteroidales	Prevotellaceae	Prevotella_9	NA
UPSIT.score	ASV_1af_96k	0,037575085	0,523502641	0,900126354	Bacteria	Firmicutes	Bacilli	Lactobacillales	Carnobacteriaceae	Dolosigranulum	NA
UPSIT.score	ASV_1dc_ewy	0,017219191	0,543252457	0,912158714	Bacteria	Firmicutes	Bacilli	Lactobacillales	Carnobacteriaceae	Dolosigranulum	NA

Variable	FeatureID	spearman.erho	spearman.ep	spearman.eBH	Domain	Phylum	Class	Order	Family	Genus	Species
UPSIT.score	ASV_1kn_zel	-0,000351245	0,643194168	0,944034754	Bacteria	Actinobacteriota	Actinobacteria	Corynebacteriales	Corynebacteriaceae	Lawsonella	NA
UPSIT.score	ASV_1ob_3tg	0,027241937	0,493566387	0,8939262	Bacteria	Firmicutes	Bacilli	Staphylococcales	Staphylococcaceae	Staphylococcus	NA
UPSIT.score	ASV_1wh_81x	-0,012399778	0,548101115	0,907452761	Bacteria	Firmicutes	Negativicutes	Veillonellales-Selenomonadales	Veillonellaceae	Negativicoccus	succinicivorans
UPSIT.score	ASV_242_lzz	0,269625743	0,295054931	0,863578791	Bacteria	Actinobacteriota	Actinobacteria	Corynebacteriales	Corynebacteriaceae	Corynebacterium	aurimucosum/pseudogenitalium/tuberculostearicum
UPSIT.score	ASV_2d3_euj	-0,359732432	0,209888119	0,774725455	Bacteria	Actinobacteriota	Actinobacteria	Corynebacteriales	Corynebacteriaceae	Corynebacterium	NA
UPSIT.score	ASV_2db_0um	0,163990742	0,445264301	0,876622567	Bacteria	Firmicutes	Negativicutes	Veillonellales-Selenomonadales	Veillonellaceae	Dialister	NA
UPSIT.score	ASV_2ee_ogi	-0,012677507	0,668826194	0,941451785	Bacteria	Firmicutes	Bacilli	Staphylococcales	Staphylococcaceae	Staphylococcus	lugdunensis
UPSIT.score	ASV_2o1_p31	0,005587252	0,492218003	0,896107386	Bacteria	Firmicutes	Clostridia	Lachnospirales	Lachnospiraceae	Lachnospiraceae AC2044 group	bacterium
UPSIT.score	ASV_2x6_j18	0,091478996	0,486067818	0,878465662	Bacteria	Firmicutes	Clostridia	Peptostreptococcales-Tissierellales	Peptostreptococcaceae	Criibacterium	NA
UPSIT.score	ASV_2xa_4rw	0,194042642	0,419386296	0,87739993	Bacteria	Bacteroidota	Bacteroidia	Bacteroidales	Prevotellaceae	Prevotella_9	NA
UPSIT.score	ASV_2xm_w96	0,191722789	0,43656473	0,873614617	Bacteria	Firmicutes	Clostridia	Lachnospirales	Lachnospiraceae	Dorea	formicigenerans
UPSIT.score	ASV_30c_6sw	0,01809322	0,537248935	0,914564621	Bacteria	Proteobacteria	Gammaproteobacteria	Burkholderiales	Oxalobacteraceae	Massilia	timonae
UPSIT.score	ASV_339_mvy	0,012710181	0,495680797	0,898255109	Bacteria	Proteobacteria	Gammaproteobacteria	Enterobacterales	Pasteurellaceae	Haemophilus	NA
UPSIT.score	ASV_3em_n0d	0,191371544	0,450679948	0,857867506	Bacteria	Firmicutes	Bacilli	Lactobacillales	Lactobacillaceae	Liquorilactobacillus	NA
UPSIT.score	ASV_3en_7ow	0,150782283	0,450423756	0,880639254	Bacteria	Firmicutes	Bacilli	Staphylococcales	Gemellaceae	Gemella	morbillorum
UPSIT.score	ASV_3i5_y1w	-0,002728278	0,580012302	0,927689366	Bacteria	Proteobacteria	Gammaproteobacteria	Enterobacterales	Pasteurellaceae	Haemophilus	NA
UPSIT.score	ASV_3kg_4bk	0,025011937	0,630901125	0,936965743	Bacteria	Firmicutes	Bacilli	Lactobacillales	Carnobacteriaceae	Dolosigranulum	pigrum
UPSIT.score	ASV_3kh_nw9	-0,290030648	0,301863808	0,835571475	Bacteria	Actinobacteriota	Actinobacteria	Corynebacteriales	Corynebacteriaceae	Corynebacterium	NA
UPSIT.score	ASV_3pn_tj1	0,069914164	0,549180281	0,904190107	Bacteria	Actinobacteriota	Actinobacteria	Corynebacteriales	Corynebacteriaceae	Corynebacterium	NA
UPSIT.score	ASV_3tk_6ez	0,159857483	0,483008454	0,889341363	Bacteria	Actinobacteriota	Actinobacteria	Corynebacteriales	Corynebacteriaceae	Corynebacterium	NA
UPSIT.score	ASV_440_o98	0,00755586	0,522524429	0,903609953	Bacteria	Firmicutes	Clostridia	Peptostreptococcales-Tissierellales	Family XI	Anaerococcus	NA
UPSIT.score	ASV_46z_g65	0,146967595	0,476625821	0,880691335	Bacteria	Firmicutes	Negativicutes	Veillonellales-Selenomonadales	Veillonellaceae	Negativicoccus	NA
UPSIT.score	ASV_47r_9ra	-0,193315646	0,397905747	0,86931959	Bacteria	Actinobacteriota	Actinobacteria	Corynebacteriales	Corynebacteriaceae	Corynebacterium	NA
UPSIT.score	ASV_49k_gjb	0,144745764	0,448536617	0,879515999	Bacteria	Firmicutes	Clostridia	Oscillospirales	Oscillospiraceae	UCG-002	NA
UPSIT.score	ASV_4b4_zwy	-0,046715633	0,499723897	0,899102318	Bacteria	Campylobacterota	Campylobacteria	Campylobacterales	Campylobacteraceae	Campylobacter	ureolyticus

Variable	FeatureID	spearman.erho	spearman.ep	spearman.eBH	Domain	Phylum	Class	Order	Family	Genus	Species
UPSIT.score	ASV_4cz_ka1	0,149646862	0,434728559	0,873826373	Bacteria	Firmicutes	Bacilli	Staphylococcales	Staphylococcaceae	Staphylococcus	NA
UPSIT.score	ASV_4kw_hck	0,159024296	0,462975788	0,883880591	Bacteria	Firmicutes	Clostridia	Peptostreptococcales-Tissierellales	Family XI	Anaerococcus	NA
UPSIT.score	ASV_4m8_kdu	0,164652391	0,459795959	0,876152206	Bacteria	Bacteroidota	Bacteroidia	Bacteroidales	Prevotellaceae	Prevotella_9	NA
UPSIT.score	ASV_4ni_ro3	0,048055267	0,553796922	0,907728247	Bacteria	Actinobacteriota	Actinobacteria	Corynebacteriales	Corynebacteriaceae	Corynebacterium	NA
UPSIT.score	ASV_4pz_tc7	-0,176692755	0,500337146	0,918130255	Bacteria	Firmicutes	Bacilli	Staphylococcales	Staphylococcaceae	Staphylococcus	argenteus/aureus/equorum/phage/schweitzeri/simiae
UPSIT.score	ASV_4qr_yd2	0,156099974	0,449875667	0,882708805	Bacteria	Firmicutes	Bacilli	Lactobacillales	Streptococcaceae	Streptococcus	NA
UPSIT.score	ASV_4rd_dxc	-0,018281096	0,663939442	0,947674265	Bacteria	Actinobacteriota	Actinobacteria	Corynebacteriales	Corynebacteriaceae	Corynebacterium	NA
UPSIT.score	ASV_513_wtm	0,083008265	0,512708886	0,904328693	Bacteria	Proteobacteria	Gammaproteobacteria	Enterobacterales	Enterobacteriaceae	Raoultella	NA
UPSIT.score	ASV_53d_zfm	-0,091160425	0,717794238	0,957462675	Bacteria	Actinobacteriota	Actinobacteria	Corynebacteriales	Corynebacteriaceae	Lawsonella	NA
UPSIT.score	ASV_53v_62f	-0,207994435	0,433998826	0,899920862	Bacteria	Firmicutes	Bacilli	Staphylococcales	Staphylococcaceae	Staphylococcus	NA
UPSIT.score	ASV_566_wbp	-0,202742091	0,45085689	0,873067424	Bacteria	Actinobacteriota	Actinobacteria	Corynebacteriales	Corynebacteriaceae	Corynebacterium	NA
UPSIT.score	ASV_5do_e0k	0,066303689	0,516914009	0,894243281	Bacteria	Actinobacteriota	Actinobacteria	Corynebacteriales	Corynebacteriaceae	Corynebacterium	NA
UPSIT.score	ASV_5j9_202	0,270605963	0,324968188	0,849348942	Bacteria	Actinobacteriota	Actinobacteria	Corynebacteriales	Corynebacteriaceae	Corynebacterium	NA
UPSIT.score	ASV_5mh_1v6	-0,014286701	0,536502126	0,913114171	Bacteria	Firmicutes	Bacilli	Staphylococcales	Staphylococcaceae	Staphylococcus	NA
UPSIT.score	ASV_5oo_dg6	0,192474291	0,447207445	0,870893317	Bacteria	Actinobacteriota	Actinobacteria	Micrococcales	Micrococcaceae	Rothia	mucilaginosa
UPSIT.score	ASV_5r4_ie2	-0,115143131	0,514665741	0,899887942	Bacteria	Actinobacteriota	Actinobacteria	Corynebacteriales	Corynebacteriaceae	Corynebacterium	NA
UPSIT.score	ASV_5xz_bqw	-0,030092742	0,561971126	0,925380699	Bacteria	Firmicutes	Bacilli	Lactobacillales	Lactobacillaceae	Liquorilactobacillus	NA
UPSIT.score	ASV_66n_hd0	-0,220957839	0,428514777	0,882948521	Bacteria	Firmicutes	Bacilli	Staphylococcales	Staphylococcaceae	Staphylococcus	aureus
UPSIT.score	ASV_6dr_o2s	0,027299116	0,5358387	0,898407159	Bacteria	Firmicutes	Bacilli	Staphylococcales	Staphylococcaceae	Staphylococcus	NA
UPSIT.score	ASV_6ut_tpw	0,022741095	0,531260036	0,9155417	Bacteria	Proteobacteria	Gammaproteobacteria	Burkholderiales	Comamonadaceae	Tepidimonas	NA
UPSIT.score	ASV_727_4zo	0,038277576	0,499017481	0,904172654	Bacteria	Firmicutes	Bacilli	Lactobacillales	Carnobacteriaceae	Dolosigranulum	NA
UPSIT.score	ASV_79g_5uy	0,160094369	0,428231703	0,872603362	Bacteria	Firmicutes	Clostridia	Lachnospirales	Lachnospiraceae	Roseburia	NA
UPSIT.score	ASV_7cr_wom	-0,142074665	0,535270865	0,910707711	Bacteria	Firmicutes	Bacilli	Staphylococcales	Staphylococcaceae	Staphylococcus	NA
UPSIT.score	ASV_7jt_wq3	0,225826263	0,399430732	0,863850176	Bacteria	Actinobacteriota	Actinobacteria	Corynebacteriales	Corynebacteriaceae	Corynebacterium	NA
UPSIT.score	ASV_7ka_s2c	-0,126056243	0,510065987	0,895923614	Bacteria	Actinobacteriota	Actinobacteria	Corynebacteriales	Corynebacteriaceae	Corynebacterium	NA
UPSIT.score	ASV_7x9_tvb	0,04265589	0,849655427	0,972263069	Bacteria	Firmicutes	Clostridia	Peptostreptococcales-Tissierellales	Family XI	Peptoniphilus	gorbachii/lacydonensis/rhinitidis

Variable	FeatureID	spearman.erho	spearman.ep	spearman.eBH	Domain	Phylum	Class	Order	Family	Genus	Species
UPSIT.score	ASV_873_9b5	-0,192049529	0,439568654	0,877924826	Bacteria	Firmicutes	Bacilli	Staphylococcales	Staphylococcaceae	NA	NA
UPSIT.score	ASV_8lf_xek	0,149883748	0,489944707	0,898321736	Bacteria	Bacteroidota	Bacteroidia	Bacteroidales	Prevotellaceae	Prevotella_9	copri
UPSIT.score	ASV_8lv_u9v	0,034005452	0,4909927	0,89944648	Bacteria	Firmicutes	Bacilli	Lactobacillales	Streptococcaceae	Streptococcus	NA
UPSIT.score	ASV_8pb_fin	0,16954532	0,432095474	0,873353514	Bacteria	Bacteroidota	Bacteroidia	Cytophagales	Hymenobacteraceae	Hymenobacter	NA
UPSIT.score	ASV_8re_um4	0,534366725	0,058412207	0,581117083	Bacteria	Firmicutes	Bacilli	Staphylococcales	Staphylococcaceae	Staphylococcus	NA
UPSIT.score	ASV_8v2_sgp	-0,015258752	0,596729337	0,933121532	Bacteria	Actinobacteriota	Actinobacteria	Corynebacteriales	Corynebacteriaceae	Corynebacterium	NA
UPSIT.score	ASV_8zu_e97	-0,083645408	0,500398105	0,906090221	Bacteria	Firmicutes	Bacilli	Staphylococcales	Staphylococcaceae	Staphylococcus	NA
UPSIT.score	ASV_926_7zf	-0,244295235	0,379729693	0,87356148	Bacteria	Firmicutes	Bacilli	Staphylococcales	Staphylococcaceae	Staphylococcus	NA
UPSIT.score	ASV_9cd_tui	0,126660712	0,460485418	0,884231916	Bacteria	Firmicutes	Bacilli	Lactobacillales	Enterococcaceae	Tetragenococcus	NA
UPSIT.score	ASV_9d0_o06	-0,071359988	0,567980287	0,913284852	Bacteria	Firmicutes	Negativicutes	Veillonellales-Selenomonadales	Veillonellaceae	Negativicoccus	NA
UPSIT.score	ASV_9eq_kr6	0,144173969	0,473030129	0,886836272	Bacteria	Firmicutes	Bacilli	Lactobacillales	Streptococcaceae	Streptococcus	mitis/parasanguinis
UPSIT.score	ASV_9jb_2ek	-0,220541246	0,412044519	0,888037129	Bacteria	Actinobacteriota	Actinobacteria	Corynebacteriales	Corynebacteriaceae	Corynebacterium	NA
UPSIT.score	ASV_9kg_f3f	0,124741115	0,460419524	0,891928896	Bacteria	Actinobacteriota	Actinobacteria	Corynebacteriales	NA	NA	NA
UPSIT.score	ASV_9l0_247	0,045277978	0,530725185	0,909486928	Bacteria	Firmicutes	Bacilli	Staphylococcales	Gemellaceae	Gemella	morbillorum
UPSIT.score	ASV_9mx_kho	-0,090572293	0,526626926	0,899991212	Bacteria	Actinobacteriota	Actinobacteria	Frankiales	Sporichthyaceae	NA	NA
UPSIT.score	ASV_9o6_1ja	-0,113819835	0,620016816	0,93301206	Bacteria	Firmicutes	Bacilli	Staphylococcales	Staphylococcaceae	Staphylococcus	aureus
UPSIT.score	ASV_9vy_jj2	-0,015846884	0,554013014	0,916859943	Bacteria	Firmicutes	Bacilli	Staphylococcales	Staphylococcaceae	Staphylococcus	NA
UPSIT.score	ASV_9wz_9u7	-0,031473218	0,56315075	0,912148343	Bacteria	Actinobacteriota	Actinobacteria	NA	NA	NA	NA
UPSIT.score	ASV_aew_fyi	0,053111566	0,523088228	0,907441634	Bacteria	Actinobacteriota	Actinobacteria	Corynebacteriales	Corynebacteriaceae	Corynebacterium	mucifaciens
UPSIT.score	ASV_ajo_fen	-0,245283623	0,38713205	0,868423572	Bacteria	Actinobacteriota	Actinobacteria	Corynebacteriales	Corynebacteriaceae	Corynebacterium	NA
UPSIT.score	ASV_aqj_vcn	0,199131616	0,437642549	0,870755765	Bacteria	Firmicutes	Bacilli	Lactobacillales	Streptococcaceae	Streptococcus	mitis/phage/pneumoniae/pseudopneumoniae
UPSIT.score	ASV_arl_xwg	0,17220825	0,438064195	0,877511052	Bacteria	Firmicutes	Clostridia	Lachnospirales	Lachnospiraceae	Blautia	NA
UPSIT.score	ASV_aw8_q96	0,045931458	0,646040289	0,939606818	Bacteria	Actinobacteriota	Actinobacteria	Corynebacteriales	Corynebacteriaceae	Corynebacterium	NA
UPSIT.score	ASV_bg2_zbh	0,196574876	0,423180514	0,854851903	Bacteria	Firmicutes	Bacilli	Staphylococcales	Staphylococcaceae	Staphylococcus	NA
UPSIT.score	ASV_bh3_94k	0,159154992	0,454567128	0,868421597	Bacteria	Firmicutes	Bacilli	Lactobacillales	Streptococcaceae	Lactococcus	NA

Variable	FeatureID	spearman.erho	spearman.ep	spearman.eBH	Domain	Phylum	Class	Order	Family	Genus	Species
UPSIT.score	ASV_bnk_uhh	-0,155871256	0,555307638	0,912892328	Bacteria	Firmicutes	Clostridia	Peptostreptococcales-Tissierellales	Family XI	Anaerococcus	provencensis
UPSIT.score	ASV_bsd_ayc	-0,01358421	0,522578593	0,911394244	Bacteria	Planctomycetota	Planctomycetes	Pirellulales	Pirellulaceae	NA	NA
UPSIT.score	ASV_btl_vxl	-0,080410683	0,559990567	0,918959979	Bacteria	Firmicutes	Clostridia	Peptostreptococcales-Tissierellales	Family XI	Anaerococcus	vaginalis
UPSIT.score	ASV_bwx_wef	-0,114791886	0,515792207	0,903739646	Bacteria	Actinobacteriota	Actinobacteria	Corynebacteriales	Corynebacteriaceae	Corynebacterium	amycolatum/jeikeium/lactis/urealyticum/vitaeruminis
UPSIT.score	ASV_c46_jpb	0,095522402	0,510390647	0,902566877	Bacteria	Actinobacteriota	Actinobacteria	Corynebacteriales	Corynebacteriaceae	Corynebacterium	NA
UPSIT.score	ASV_c4o_xms	-0,160167886	0,461058037	0,892721551	Bacteria	Firmicutes	Bacilli	Staphylococcales	Staphylococcaceae	NA	NA
UPSIT.score	ASV_cep_rux	-0,058682481	0,568677447	0,927293501	Bacteria	Firmicutes	Clostridia	Peptostreptococcales-Tissierellales	Family XI	Anaerococcus	NA
UPSIT.score	ASV_cje_lx4	-0,012399778	0,508775709	0,890925746	Bacteria	Proteobacteria	Gammaproteobacteria	Burkholderiales	Neisseriaceae	NA	NA
UPSIT.score	ASV_co0_sbi	0,020029154	0,546447513	0,916387379	Bacteria	Actinobacteriota	Actinobacteria	Corynebacteriales	Corynebacteriaceae	Corynebacterium	NA
UPSIT.score	ASV_cqp_otd	0,188218504	0,44328877	0,86338241	Bacteria	Firmicutes	Negativicutes	Veillonellales-Selenomonadales	Veillonellaceae	Negativicoccus	NA
UPSIT.score	ASV_cy2_emp	0,011068313	0,56055494	0,913442422	Bacteria	Actinobacteriota	Actinobacteria	Corynebacteriales	Corynebacteriaceae	Corynebacterium	NA
UPSIT.score	ASV_d0q_oe1	0,188724951	0,404667475	0,862947261	Bacteria	Bacteroidota	Bacteroidia	Bacteroidales	Prevotellaceae	Prevotella_9	NA
UPSIT.score	ASV_da2_u30	-0,017807323	0,555973962	0,917727424	Bacteria	Bacteroidota	Bacteroidia	Bacteroidales	Prevotellaceae	Prevotella	buccalis
UPSIT.score	ASV_dhe_20n	-0,09575112	0,673342584	0,947233455	Bacteria	Firmicutes	Bacilli	Staphylococcales	Staphylococcaceae	Staphylococcus	aureus
UPSIT.score	ASV_dlu_bk7	-0,210608354	0,446320995	0,884870471	Bacteria	Proteobacteria	Gammaproteobacteria	Burkholderiales	Neisseriaceae	NA	NA
UPSIT.score	ASV_dmt_o1x	0,244058348	0,378807365	0,860979323	Bacteria	Proteobacteria	Gammaproteobacteria	Enterobacterales	Pasteurellaceae	Haemophilus	haemolyticus/influenzae
UPSIT.score	ASV_dui_4x1	0,027666699	0,53603837	0,913250488	Bacteria	Firmicutes	Bacilli	Lactobacillales	Carnobacteriaceae	Dolosigranulum	NA
UPSIT.score	ASV_dz0_fy2	-0,14704928	0,514606522	0,908558881	Bacteria	Actinobacteriota	Actinobacteria	Corynebacteriales	Corynebacteriaceae	Corynebacterium	NA
UPSIT.score	ASV_e7g_uqv	0,028646918	0,527569873	0,90501613	Bacteria	Actinobacteriota	Thermoleophilia	Gaiellales	NA	NA	NA
UPSIT.score	ASV_e8x_8cs	-0,200454912	0,447058245	0,905462247	Bacteria	Actinobacteriota	Actinobacteria	Corynebacteriales	Corynebacteriaceae	Lawsonella	NA
UPSIT.score	ASV_elz_ffm	-0,055439588	0,563278169	0,914299877	Bacteria	Actinobacteriota	Actinobacteria	Corynebacteriales	Corynebacteriaceae	Corynebacterium	NA
UPSIT.score	ASV_f1j_s2e	-0,229028314	0,434297564	0,857711339	Bacteria	Actinobacteriota	Actinobacteria	Corynebacteriales	Corynebacteriaceae	Corynebacterium	aurimucosum/simulans/striatum/xerosis
UPSIT.score	ASV_f6c_v0m	0,236110401	0,402161346	0,855380159	Bacteria	Actinobacteriota	Actinobacteria	Corynebacteriales	Corynebacteriaceae	Corynebacterium	NA
UPSIT.score	ASV_f8c_ir4	0,156247007	0,516359561	0,8942649	Bacteria	Bacteroidota	Bacteroidia	Bacteroidales	Bacteroidaceae	Bacteroides	plebeius
UPSIT.score	ASV_fb6_dje	0,096731339	0,498802011	0,901945439	Bacteria	Firmicutes	Bacilli	Staphylococcales	Staphylococcaceae	Staphylococcus	aureus/devriesei/epidermidis/haemolyticus/hominis
UPSIT.score	ASV_fgq_ohv	-0,104156503	0,566802227	0,91362218	Bacteria	Actinobacteriota	Actinobacteria	Corynebacteriales	Corynebacteriaceae	Corynebacterium	NA

Variable	FeatureID	spearman.erho	spearman.ep	spearman.eBH	Domain	Phylum	Class	Order	Family	Genus	Species
UPSIT.score	ASV_fmb_men	0,014932012	0,549115441	0,916995223	Bacteria	Actinobacteriota	Actinobacteria	Corynebacteriales	Corynebacteriaceae	Corynebacterium	NA
UPSIT.score	ASV_fqm_8pm	0,112627234	0,59134139	0,92713132	Bacteria	Firmicutes	Bacilli	Lactobacillales	Streptococcaceae	Streptococcus	anginosus/cristatus/infantis/mitis/oralis/pneumoniae/pseudopneumoniae/sanguinis/timonensis
UPSIT.score	ASV_fug_ldj	0,17161195	0,456895511	0,871975114	Bacteria	Bacteroidota	Bacteroidia	Bacteroidales	Prevotellaceae	Prevotella_9	NA
UPSIT.score	ASV_g3d_cs4	0,014057983	0,529954517	0,904181213	Bacteria	Proteobacteria	Gammaproteobacteria	Enterobacterales	Pasteurellaceae	Haemophilus	influenzae
UPSIT.score	ASV_geq_907	-0,011811646	0,786051159	0,965280372	Bacteria	Firmicutes	Clostridia	Peptostreptococcales-Tissierellales	Family XI	Anaerococcus	octavius
UPSIT.score	ASV_gg8_mtq	0,021858898	0,499301216	0,890836042	Bacteria	Bacteroidota	Bacteroidia	Bacteroidales	Muribaculaceae	NA	NA
UPSIT.score	ASV_gzb_mis	0,132925949	0,476979958	0,879950225	Bacteria	Firmicutes	Clostridia	Lachnospirales	Lachnospiraceae	Coprococcus	catus
UPSIT.score	ASV_h1r_zyt	-0,015013697	0,562986009	0,9193145	Bacteria	Fusobacteriota	Fusobacteriia	Fusobacteriales	Fusobacteriaceae	Fusobacterium	NA
UPSIT.score	ASV_h40_0md	-0,288658341	0,31317297	0,845742222	Bacteria	Actinobacteriota	Actinobacteria	Corynebacteriales	Corynebacteriaceae	Corynebacterium	NA
UPSIT.score	ASV_h4w_6wi	0,063942993	0,513571181	0,905176454	Bacteria	Firmicutes	Bacilli	Staphylococcales	Staphylococcaceae	NA	NA
UPSIT.score	ASV_hbi_ebs	0,010063588	0,604585319	0,93288684	Bacteria	Actinobacteriota	Actinobacteria	Corynebacteriales	Corynebacteriaceae	Lawsonella	clevelandensis
UPSIT.score	ASV_het_bu3	-0,001347802	0,529849642	0,902788279	Bacteria	Campylobacterota	Campylobacteria	Campylobacterales	Campylobacteraceae	Campylobacter	NA
UPSIT.score	ASV_hjn_92c	0,074921453	0,743664845	0,958912303	Bacteria	Firmicutes	Clostridia	Peptostreptococcales-Tissierellales	Family XI	Finegoldia	magna
UPSIT.score	ASV_hkw_6s3	0,007204614	0,540095932	0,908206616	Bacteria	Actinobacteriota	Actinobacteria	Corynebacteriales	Corynebacteriaceae	Corynebacterium	NA
UPSIT.score	ASV_hpm_svr	-0,259129225	0,364100511	0,852905088	Bacteria	Actinobacteriota	Actinobacteria	Corynebacteriales	Corynebacteriaceae	Corynebacterium	NA
UPSIT.score	ASV_hq8_93x	-0,26013395	0,35557782	0,862344973	Bacteria	Actinobacteriota	Actinobacteria	Corynebacteriales	Corynebacteriaceae	Corynebacterium	NA
UPSIT.score	ASV_hqq_2ph	0,018297433	0,536356215	0,914381059	Bacteria	Proteobacteria	Gammaproteobacteria	Burkholderiales	Oxalobacteraceae	Massilia	timonae
UPSIT.score	ASV_i0i_mxo	0,25671135	0,349866842	0,847299439	Bacteria	Actinobacteriota	Actinobacteria	Corynebacteriales	Corynebacteriaceae	Corynebacterium	pyruviciproducens
UPSIT.score	ASV_i35_0op	-0,414665573	0,115497453	0,760294355	Bacteria	Actinobacteriota	Actinobacteria	Corynebacteriales	Corynebacteriaceae	Corynebacterium	aurimucosum/jeikeium/propinquum/pseudodiphtheriticum
UPSIT.score	ASV_i88_mm0	-0,06102684	0,590791012	0,921483542	Bacteria	Actinobacteriota	Actinobacteria	Corynebacteriales	Corynebacteriaceae	Corynebacterium	NA
UPSIT.score	ASV_ic5_nai	0,197620444	0,421639108	0,862931774	Bacteria	Firmicutes	Bacilli	Lactobacillales	Streptococcaceae	Streptococcus	sobrinus
UPSIT.score	ASV_igm_m49	0,012751024	0,527114122	0,911069331	Bacteria	NA	NA	NA	NA	NA	NA
UPSIT.score	ASV_ii5_tjz	-0,209685314	0,44228072	0,875204216	Bacteria	Firmicutes	Bacilli	Staphylococcales	Staphylococcaceae	Staphylococcus	NA
UPSIT.score	ASV_ikg_ra0	-0,326658188	0,252422798	0,813689817	Bacteria	Actinobacteriota	Actinobacteria	Corynebacteriales	Corynebacteriaceae	Corynebacterium	NA
UPSIT.score	ASV_iov_l70	0,039511019	0,54745835	0,906770879	Bacteria	Firmicutes	Bacilli	Lactobacillales	Carnobacteriaceae	Dolosigranulum	pigrum

Variable	FeatureID	spearman.erho	spearman.ep	spearman.eBH	Domain	Phylum	Class	Order	Family	Genus	Species
UPSIT.score	ASV_iqo_ea7	0,12232324	0,474355397	0,887949457	Bacteria	Firmicutes	Bacilli	Staphylococcales	Staphylococcaceae	Staphylococcus	NA
UPSIT.score	ASV_jjw_h6c	0,432227841	0,076147776	0,759724054	Bacteria	Actinobacteriota	Actinobacteria	Corynebacteriales	Corynebacteriaceae	Corynebacterium	NA
UPSIT.score	ASV_jl5_6pn	-0,066556912	0,648285003	0,94125112	Bacteria	Firmicutes	Bacilli	Staphylococcales	Staphylococcaceae	Staphylococcus	aureus
UPSIT.score	ASV_jp2_wik	0,261506257	0,3228985	0,8685305	Bacteria	Firmicutes	Bacilli	Staphylococcales	Staphylococcaceae	Staphylococcus	epidermidis
UPSIT.score	ASV_jph_cup	-0,347422507	0,203878688	0,803008496	Bacteria	Actinobacteriota	Actinobacteria	Corynebacteriales	Corynebacteriaceae	Corynebacterium	NA
UPSIT.score	ASV_jy3_f61	0,141862284	0,47848946	0,888375135	Bacteria	Actinobacteriota	Actinobacteria	Corynebacteriales	Corynebacteriaceae	Corynebacterium	NA
UPSIT.score	ASV_jy9_jqd	0,159449058	0,438626577	0,882757828	Bacteria	Firmicutes	Clostridia	Lachnospirales	Lachnospiraceae	[Eubacterium] ventriosum group	NA
UPSIT.score	ASV_k2s_cqm	-0,191853485	0,474810326	0,895146293	Bacteria	Actinobacteriota	Actinobacteria	Corynebacteriales	Corynebacteriaceae	Corynebacterium	NA
UPSIT.score	ASV_k3h_3z5	0,142311552	0,475911175	0,880315051	Bacteria	Proteobacteria	Gammaproteobacteria	Xanthomonadales	Xanthomonadaceae	Lysobacter	NA
UPSIT.score	ASV_k3h_qcz	0,113697307	0,472777634	0,877070662	Bacteria	Firmicutes	Bacilli	Staphylococcales	Staphylococcaceae	Staphylococcus	NA
UPSIT.score	ASV_kaj_b2n	0,035042851	0,515625288	0,897891526	Bacteria	Firmicutes	Bacilli	Lactobacillales	P5D1-392	NA	NA
UPSIT.score	ASV_kcs_cbi	-0,122298734	0,486761325	0,892006594	Bacteria	Actinobacteriota	Actinobacteria	Corynebacteriales	Corynebacteriaceae	Corynebacterium	NA
UPSIT.score	ASV_ki3_dfk	-0,110446246	0,55763083	0,921087754	Bacteria	Actinobacteriota	Actinobacteria	Corynebacteriales	Corynebacteriaceae	Corynebacterium	NA
UPSIT.score	ASV_kkl_mxy	-0,036366148	0,677416308	0,949873219	Bacteria	Actinobacteriota	Actinobacteria	Corynebacteriales	Corynebacteriaceae	Corynebacterium	kroppenstedtii
UPSIT.score	ASV_kru_fff	0,23192813	0,395542869	0,843014008	Bacteria	Proteobacteria	Gammaproteobacteria	Enterobacterales	Erwiniaceae	NA	NA
UPSIT.score	ASV_ktj_lyl	0,023435417	0,539143899	0,905479311	Bacteria	Proteobacteria	Gammaproteobacteria	Burkholderiales	Neisseriaceae	NA	NA
UPSIT.score	ASV_l3n_w4d	-0,005603589	0,540662656	0,911829946	Bacteria	Synergistota	Synergistia	Synergistales	Synergistaceae	Jonquetella	anthropi
UPSIT.score	ASV_lky_mqj	-0,006804358	0,565061034	0,916403052	Bacteria	Firmicutes	Bacilli	Lactobacillales	Streptococcaceae	Streptococcus	NA
UPSIT.score	ASV_lmq_jff	0,026474098	0,565547113	0,918274693	Bacteria	Firmicutes	Bacilli	Lactobacillales	Carnobacteriaceae	Dolosigranulum	NA
UPSIT.score	ASV_lnh_9fs	0,171987701	0,475562672	0,892943701	Bacteria	Actinobacteriota	Actinobacteria	Corynebacteriales	Corynebacteriaceae	Corynebacterium	afermentans/coyleae
UPSIT.score	ASV_lvd_sr9	-0,199711579	0,463659577	0,888043793	Bacteria	Actinobacteriota	Actinobacteria	Corynebacteriales	Corynebacteriaceae	Corynebacterium	NA
UPSIT.score	ASV_lvh_wn0	0,165918508	0,493646972	0,899119483	Bacteria	Actinobacteriota	Actinobacteria	Micrococcales	Dermabacteraceae	Dermabacter	jinjuensis
UPSIT.score	ASV_m52_gpv	0,044771531	0,517829267	0,903563585	Bacteria	Actinobacteriota	Actinobacteria	Corynebacteriales	Corynebacteriaceae	Corynebacterium	NA
UPSIT.score	ASV_mbn_3b6	0,161058252	0,440214696	0,872496535	Bacteria	Firmicutes	Clostridia	Lachnospirales	Lachnospiraceae	[Ruminococcus] torques group	NA
UPSIT.score	ASV_mcc_ype	0,214504727	0,441746922	0,88046103	Bacteria	Firmicutes	Bacilli	Staphylococcales	Staphylococcaceae	Staphylococcus	epidermidis
UPSIT.score	ASV_mdt_jke	-0,002099304	0,546520585	0,902886534	Bacteria	Proteobacteria	Gammaproteobacteria	Enterobacterales	Pasteurellaceae	Haemophilus	haemolyticus/influenzae

Variable	FeatureID	spearman.erho	spearman.ep	spearman.eBH	Domain	Phylum	Class	Order	Family	Genus	Species
UPSIT.score	ASV_mgr_u9o	-0,13558071	0,506892429	0,908519392	Bacteria	Actinobacteriota	Actinobacteria	Corynebacteriales	Corynebacteriaceae	Corynebacterium	NA
UPSIT.score	ASV_mlb_2sd	-0,04619285	0,548266492	0,918898037	Bacteria	Firmicutes	Bacilli	Staphylococcales	Staphylococcaceae	Staphylococcus	NA
UPSIT.score	ASV_mm9_js5	-0,215975056	0,426198676	0,874389914	Bacteria	Firmicutes	Bacilli	Staphylococcales	Staphylococcaceae	Staphylococcus	aureus
UPSIT.score	ASV_mvn_oem	-0,098503903	0,529985042	0,906389785	Bacteria	Actinobacteriota	Actinobacteria	Corynebacteriales	Corynebacteriaceae	Corynebacterium	NA
UPSIT.score	ASV_n1a_pw6	-0,096282072	0,534801268	0,898387011	Bacteria	Proteobacteria	Alphaproteobacteria	Rhizobiales	Methyloligellaceae	NA	NA
UPSIT.score	ASV_n5j_9sq	-0,049443911	0,538384346	0,91066003	Bacteria	Firmicutes	Bacilli	Staphylococcales	Staphylococcaceae	Staphylococcus	NA
UPSIT.score	ASV_n79_65k	0,079291599	0,502427252	0,896824529	Bacteria	Proteobacteria	Gammaproteobacteria	Burkholderiales	Neisseriaceae	NA	NA
UPSIT.score	ASV_nbq_6pg	0,131324923	0,43807619	0,867303068	Bacteria	Firmicutes	Clostridia	Peptostreptococcales-Tissierellales	Family XI	Anaerococcus	prevotii/tetradius
UPSIT.score	ASV_nk5_hwz	-0,006036519	0,542768082	0,903765316	Bacteria	Bacteroidota	Bacteroidia	Flavobacteriales	Weeksellaceae	Cloacibacterium	normanense
UPSIT.score	ASV_ntr_hh5	0,062260283	0,515464217	0,908608619	Bacteria	Proteobacteria	Gammaproteobacteria	Enterobacterales	Pasteurellaceae	Haemophilus	haemolyticus/influenzae/parainfluenzae
UPSIT.score	ASV_nvc_oao	0,017652122	0,688991004	0,949792577	Bacteria	Deinococcota	Deinococci	Thermales	Thermaceae	Thermus	parvatiensis/thermophilus
UPSIT.score	ASV_o0r_len	0,243315015	0,357745101	0,881549285	Bacteria	Firmicutes	Bacilli	Staphylococcales	Staphylococcaceae	Staphylococcus	aureus/capitis/caprae/epidermidis/haemolyticus/saprophyticus/warneri
UPSIT.score	ASV_o10_2p1	0,074406838	0,541008258	0,910160196	Bacteria	Proteobacteria	Gammaproteobacteria	Pseudomonadales	Pseudomonadaceae	Pseudomonas	brassicacearum/chlororaphis/corrugata/fluorescens/jessenii/kilonensis/mohnii/putida/synxantha
UPSIT.score	ASV_o1f_10k	0,0272256	0,585271813	0,926063062	Bacteria	Proteobacteria	Gammaproteobacteria	Burkholderiales	Comamonadaceae	NA	NA
UPSIT.score	ASV_ode_i48	-0,01538128	0,558046526	0,912774647	Bacteria	Actinobacteriota	Actinobacteria	Corynebacteriales	Corynebacteriaceae	Corynebacterium	NA
UPSIT.score	ASV_on0_uxs	0,079863393	0,627263393	0,933528033	Bacteria	Actinobacteriota	Actinobacteria	Corynebacteriales	Corynebacteriaceae	Corynebacterium	afermentans/ihumii
UPSIT.score	ASV_onw_luq	-0,032232888	0,548032346	0,903099337	Bacteria	Proteobacteria	Gammaproteobacteria	Burkholderiales	Neisseriaceae	Eikenella	NA
UPSIT.score	ASV_ouf_s4v	0,203011652	0,404541196	0,856122331	Bacteria	Actinobacteriota	Actinobacteria	Corynebacteriales	Corynebacteriaceae	Corynebacterium	NA
UPSIT.score	ASV_oyd_63r	-0,059736217	0,554261378	0,919107151	Bacteria	Firmicutes	Bacilli	Staphylococcales	Staphylococcaceae	Staphylococcus	NA
UPSIT.score	ASV_p9u_72k	-0,001707216	0,534736193	0,917427055	Bacteria	Firmicutes	Clostridia	Peptococcales	Peptococcaceae	Peptococcus	NA
UPSIT.score	ASV_paz_uyl	0,318628556	0,222624248	0,831138887	Bacteria	Actinobacteriota	Actinobacteria	Propionibacteriales	Propionibacteriaceae	Cutibacterium	acnes/avidum
UPSIT.score	ASV_pix_o0d	0,011876994	0,564890598	0,924299644	Bacteria	Desulfobacterota	Desulfovibrionia	Desulfovibrionales	Desulfohalobiaceae	Desulfovermiculus	NA
UPSIT.score	ASV_pjp_yzq	0,246966333	0,37781504	0,83939752	Bacteria	Firmicutes	Bacilli	Staphylococcales	Staphylococcaceae	Staphylococcus	NA
UPSIT.score	ASV_pni_ryk	0,049002812	0,519706164	0,895974049	Bacteria	Actinobacteriota	Actinobacteria	Corynebacteriales	Corynebacteriaceae	Corynebacterium	NA
UPSIT.score	ASV_pwy_pjt	-0,200797989	0,459531732	0,882929664	Bacteria	Actinobacteriota	Actinobacteria	Corynebacteriales	Corynebacteriaceae	Corynebacterium	NA

Variable	FeatureID	spearman.erho	spearman.ep	spearman.eBH	Domain	Phylum	Class	Order	Family	Genus	Species
UPSIT.score	ASV_q1f_0em	-0,16954532	0,516867039	0,902069627	Bacteria	Actinobacteriota	Actinobacteria	Corynebacteriales	Corynebacteriaceae	Corynebacterium	NA
UPSIT.score	ASV_q1k_ad2	-0,200691799	0,444329607	0,891270685	Bacteria	Firmicutes	Bacilli	Staphylococcales	Staphylococcaceae	Staphylococcus	NA
UPSIT.score	ASV_q1o_24k	-0,401775685	0,102662856	0,774440411	Bacteria	Actinobacteriota	Actinobacteria	Corynebacteriales	Corynebacteriaceae	Corynebacterium	accolens/fastidiosum
UPSIT.score	ASV_q1r_s4c	0,010145273	0,484676975	0,89907347	Bacteria	Actinobacteriota	Actinobacteria	Actinomycetales	Actinomycetaceae	Actinotignum	schaalii/timonense
UPSIT.score	ASV_qc8_76v	0,109139286	0,522814927	0,908662801	Bacteria	Proteobacteria	Gammaproteobacteria	Enterobacterales	Pasteurellaceae	Haemophilus	influenzae
UPSIT.score	ASV_qft_24l	-0,041381605	0,545903002	0,910613466	Bacteria	Bacteroidota	Bacteroidia	Bacteroidales	Prevotellaceae	Alloprevotella	NA
UPSIT.score	ASV_qis_xxl	0,156189828	0,463117714	0,875981338	Bacteria	Firmicutes	Clostridia	Peptostreptococcales-Tissierellales	Family XI	Anaerococcus	nagyae
UPSIT.score	ASV_qk1_qy8	0,039535525	0,511706936	0,897942189	Bacteria	Proteobacteria	Gammaproteobacteria	Enterobacterales	Pasteurellaceae	Lonepinella	NA
UPSIT.score	ASV_r73_72u	0,05178827	0,465863844	0,884205555	Bacteria	Actinobacteriota	Actinobacteria	Corynebacteriales	Corynebacteriaceae	Corynebacterium	NA
UPSIT.score	ASV_r8p_k1e	-0,218352089	0,419190782	0,884738241	Bacteria	Firmicutes	Bacilli	Staphylococcales	Staphylococcaceae	Staphylococcus	aureus
UPSIT.score	ASV_r9n_48y	0,131643495	0,493027991	0,889915625	Bacteria	Firmicutes	Bacilli	Staphylococcales	Staphylococcaceae	Staphylococcus	NA
UPSIT.score	ASV_rad_25g	0,04113655	0,520484534	0,908438272	Bacteria	Proteobacteria	Gammaproteobacteria	Enterobacterales	Pasteurellaceae	NA	NA
UPSIT.score	ASV_raj_rv1	0,430185717	0,080046481	0,758618075	Bacteria	Firmicutes	Bacilli	Staphylococcales	Staphylococcaceae	Staphylococcus	aureus/capitis/caprae/epidermidis/haemolyticus/warneri
UPSIT.score	ASV_rmx_t9x	-0,261555268	0,359431315	0,851797425	Bacteria	Firmicutes	Bacilli	Staphylococcales	Staphylococcaceae	Staphylococcus	aureus
UPSIT.score	ASV_rs1_k0s	-0,008282856	0,555742307	0,914960044	Bacteria	Firmicutes	Bacilli	Staphylococcales	Staphylococcaceae	Staphylococcus	NA
UPSIT.score	ASV_rtx_tpz	0,046977025	0,558327734	0,916164076	Bacteria	Proteobacteria	Gammaproteobacteria	Pseudomonadales	Moraxellaceae	Moraxella	catarrhalis/nonliquefaciens
UPSIT.score	ASV_s8r_baf	0,442708023	0,093964231	0,733106076	Bacteria	Firmicutes	Clostridia	Peptostreptococcales-Tissierellales	Family XI	Anaerococcus	NA
UPSIT.score	ASV_sjv_qdw	0,264079334	0,35447355	0,831853428	Bacteria	Actinobacteriota	Actinobacteria	Corynebacteriales	Corynebacteriaceae	Corynebacterium	NA
UPSIT.score	ASV_snw_j03	0,145015324	0,463367075	0,88432786	Bacteria	Firmicutes	Bacilli	Lactobacillales	Streptococcaceae	Streptococcus	NA
UPSIT.score	ASV_ssr_vha	-0,182974329	0,497574228	0,904240488	Bacteria	Firmicutes	Bacilli	Staphylococcales	Staphylococcaceae	Staphylococcus	aureus
UPSIT.score	ASV_t7p_n06	0,131945729	0,544128775	0,912459486	Bacteria	Firmicutes	Clostridia	Peptostreptococcales-Tissierellales	Family XI	Anaerococcus	NA
UPSIT.score	ASV_t8l_ysz	-0,358940088	0,193910461	0,792176866	Bacteria	Firmicutes	Bacilli	Staphylococcales	Staphylococcaceae	NA	NA
UPSIT.score	ASV_ten_mjo	-0,124398038	0,494886573	0,895000861	Bacteria	Actinobacteriota	Actinobacteria	Corynebacteriales	Corynebacteriaceae	Corynebacterium	NA
UPSIT.score	ASV_tu2_xgy	-0,319273867	0,263982702	0,808509239	Bacteria	Actinobacteriota	Actinobacteria	Corynebacteriales	Corynebacteriaceae	Corynebacterium	NA
UPSIT.score	ASV_tvh_ar7	-0,042427173	0,572945851	0,915127291	Bacteria	Firmicutes	Bacilli	Lactobacillales	Streptococcaceae	Streptococcus	anginosus/constellatus/intermedius/lutetiensis
UPSIT.score	ASV_v7r_7sn	-0,054165302	0,537824039	0,903372303	Bacteria	Proteobacteria	Gammaproteobacteria	Burkholderiales	Comamonadaceae	Rubrivivax	NA

Variable	FeatureID	spearman.erho	spearman.ep	spearman.eBH	Domain	Phylum	Class	Order	Family	Genus	Species
UPSIT.score	ASV_xex_kso	0,192441617	0,454443593	0,885077024	Bacteria	Actinobacteriota	Actinobacteria	Micrococcales	Bogoriellaceae	Georgenia	NA
IL.5	ASV_17p_uuy	-0,351747936	0,17635022	0,910876185	Bacteria	Actinobacteriota	Actinobacteria	Corynebacteriales	Corynebacteriaceae	Corynebacterium	NA
IL.5	ASV_19p_rjx	-0,039763932	0,542880868	0,946242703	Bacteria	Bacteroidota	Bacteroidia	Bacteroidales	Prevotellaceae	Prevotella_9	NA
IL.5	ASV_1af_96k	-0,122629644	0,506022411	0,93631922	Bacteria	Firmicutes	Bacilli	Lactobacillales	Carnobacteriaceae	Dolosigranulum	NA
IL.5	ASV_1dc_ewy	-0,253466847	0,348499843	0,900615035	Bacteria	Firmicutes	Bacilli	Lactobacillales	Carnobacteriaceae	Dolosigranulum	NA
IL.5	ASV_1kn_zel	0,207414216	0,433461612	0,937774165	Bacteria	Actinobacteriota	Actinobacteria	Corynebacteriales	Corynebacteriaceae	Lawsonella	NA
IL.5	ASV_1ob_3tg	-0,146446078	0,459583405	0,920223563	Bacteria	Firmicutes	Bacilli	Staphylococcales	Staphylococcaceae	Staphylococcus	NA
IL.5	ASV_1wh_81x	0,205366357	0,443865485	0,924697455	Bacteria	Firmicutes	Negativicutes	Veillonellales-Selenomonadales	Veillonellaceae	Negativicoccus	succinicivorans
IL.5	ASV_242_lzz	0,407572239	0,106827482	0,892959569	Bacteria	Actinobacteriota	Actinobacteria	Corynebacteriales	Corynebacteriaceae	Corynebacterium	aurimucosum/pseudogenitalium/tuberculostearicum
IL.5	ASV_2d3_euj	0,198561662	0,459481525	0,940775194	Bacteria	Actinobacteriota	Actinobacteria	Corynebacteriales	Corynebacteriaceae	Corynebacterium	NA
IL.5	ASV_2db_0um	-0,072819917	0,528897194	0,939591986	Bacteria	Firmicutes	Negativicutes	Veillonellales-Selenomonadales	Veillonellaceae	Dialister	NA
IL.5	ASV_2ee_ogi	-0,059258901	0,652479045	0,959721708	Bacteria	Firmicutes	Bacilli	Staphylococcales	Staphylococcaceae	Staphylococcus	lugdunensis
IL.5	ASV_2o1_p31	-0,171117131	0,411619206	0,915855593	Bacteria	Firmicutes	Clostridia	Lachnospirales	Lachnospiraceae	Lachnospiraceae AC2044 group	bacterium
IL.5	ASV_2x6_j18	0,039747807	0,543930146	0,946311377	Bacteria	Firmicutes	Clostridia	Peptostreptococcales-Tissierellales	Peptostreptococcaceae	Criibacterium	NA
IL.5	ASV_2xa_4rw	-0,117292312	0,515907258	0,940787605	Bacteria	Bacteroidota	Bacteroidia	Bacteroidales	Prevotellaceae	Prevotella_9	NA
IL.5	ASV_2xm_w96	-0,051825335	0,514551931	0,94495836	Bacteria	Firmicutes	Clostridia	Lachnospirales	Lachnospiraceae	Dorea	formicigenerans
IL.5	ASV_30c_6sw	-0,02559017	0,497685584	0,944827456	Bacteria	Proteobacteria	Gammaproteobacteria	Burkholderiales	Oxalobacteraceae	Massilia	timonae
IL.5	ASV_339_mvy	-0,005466331	0,542093003	0,947537274	Bacteria	Proteobacteria	Gammaproteobacteria	Enterobacterales	Pasteurellaceae	Haemophilus	NA
IL.5	ASV_3em_n0d	-0,070159314	0,516218337	0,948682017	Bacteria	Firmicutes	Bacilli	Lactobacillales	Lactobacillaceae	Liquorilactobacillus	NA
IL.5	ASV_3en_7ow	0,170730134	0,506926984	0,939156068	Bacteria	Firmicutes	Bacilli	Staphylococcales	Gemellaceae	Gemella	morbillorum
IL.5	ASV_3i5_y1w	-0,036474458	0,532512975	0,945844219	Bacteria	Proteobacteria	Gammaproteobacteria	Enterobacterales	Pasteurellaceae	Haemophilus	NA
IL.5	ASV_3kg_4bk	-0,414296311	0,155909184	0,834721842	Bacteria	Firmicutes	Bacilli	Lactobacillales	Carnobacteriaceae	Dolosigranulum	pigrum
IL.5	ASV_3kh_nw9	-0,01122291	0,625378968	0,962593789	Bacteria	Actinobacteriota	Actinobacteria	Corynebacteriales	Corynebacteriaceae	Corynebacterium	NA
IL.5	ASV_3pn_tj1	-0,119195046	0,493847222	0,933693392	Bacteria	Actinobacteriota	Actinobacteria	Corynebacteriales	Corynebacteriaceae	Corynebacterium	NA
IL.5	ASV_3tk_6ez	-0,195449561	0,419204488	0,92954867	Bacteria	Actinobacteriota	Actinobacteria	Corynebacteriales	Corynebacteriaceae	Corynebacterium	NA
IL.5	ASV_440_o98	0,140544376	0,490965132	0,938771928	Bacteria	Firmicutes	Clostridia	Peptostreptococcales-Tissierellales	Family XI	Anaerococcus	NA
IL.5	ASV_46z_g65	-0,044891641	0,489888979	0,944995937	Bacteria	Firmicutes	Negativicutes	Veillonellales-Selenomonadales	Veillonellaceae	Negativicoccus	NA
IL.5	ASV_47r_9ra	-0,147220072	0,496473639	0,937581267	Bacteria	Actinobacteriota	Actinobacteria	Corynebacteriales	Corynebacteriaceae	Corynebacterium	NA
IL.5	ASV_49k_gjb	-0,046407379	0,485758243	0,944040474	Bacteria	Firmicutes	Clostridia	Oscillospirales	Oscillospiraceae	UCG-002	NA

Variable	FeatureID	spearman.erho	spearman.ep	spearman.eBH	Domain	Phylum	Class	Order	Family	Genus	Species
IL.5	ASV_4b4_zwy	0,195304438	0,464757495	0,927814602	Bacteria	Campylobacterota	Campylobacteria	Campylobacterales	Campylobacteraceae	Campylobacter	ureolyticus
IL.5	ASV_4cz_ka1	-0,040941047	0,579845572	0,952210456	Bacteria	Firmicutes	Bacilli	Staphylococcales	Staphylococcaceae	Staphylococcus	NA
IL.5	ASV_4kw_hck	-0,069014448	0,492637859	0,933276012	Bacteria	Firmicutes	Clostridia	Peptostreptococcales-Tissierellales	Family XI	Anaerococcus	NA
IL.5	ASV_4m8_kdu	-0,055856553	0,518875627	0,938341318	Bacteria	Bacteroidota	Bacteroidia	Bacteroidales	Prevotellaceae	Prevotella_9	NA
IL.5	ASV_4ni_ro3	0,04743937	0,560102974	0,951273361	Bacteria	Actinobacteriota	Actinobacteria	Corynebacteriales	Corynebacteriaceae	Corynebacterium	NA
IL.5	ASV_4pz_tc7	0,118985423	0,642732004	0,961099231	Bacteria	Firmicutes	Bacilli	Staphylococcales	Staphylococcaceae	Staphylococcus	argenteus/aureus/equorum/phage/schweitzeri/simiae
IL.5	ASV_4qr_yd2	-0,02433243	0,588428851	0,952713866	Bacteria	Firmicutes	Bacilli	Lactobacillales	Streptococcaceae	Streptococcus	NA
IL.5	ASV_4rd_dxc	-0,116566692	0,594901814	0,953128342	Bacteria	Actinobacteriota	Actinobacteria	Corynebacteriales	Corynebacteriaceae	Corynebacterium	NA
IL.5	ASV_513_wtm	-0,013399768	0,519624128	0,946893138	Bacteria	Proteobacteria	Gammaproteobacteria	Enterobacterales	Enterobacteriaceae	Raoultella	NA
IL.5	ASV_53d_zfm	-0,028444272	0,806162802	0,97780138	Bacteria	Actinobacteriota	Actinobacteria	Corynebacteriales	Corynebacteriaceae	Lawsonella	NA
IL.5	ASV_53v_62f	0,076480263	0,732883541	0,971661038	Bacteria	Firmicutes	Bacilli	Staphylococcales	Staphylococcaceae	Staphylococcus	NA
IL.5	ASV_566_wbp	-0,014899381	0,564041224	0,950595189	Bacteria	Actinobacteriota	Actinobacteria	Corynebacteriales	Corynebacteriaceae	Corynebacterium	NA
IL.5	ASV_5do_e0k	0,097120098	0,545367175	0,951374632	Bacteria	Actinobacteriota	Actinobacteria	Corynebacteriales	Corynebacteriaceae	Corynebacterium	NA
IL.5	ASV_5j9_202	-0,323642286	0,254432089	0,900518516	Bacteria	Actinobacteriota	Actinobacteria	Corynebacteriales	Corynebacteriaceae	Corynebacterium	NA
IL.5	ASV_5mh_1v6	-0,220104489	0,408816065	0,928233235	Bacteria	Firmicutes	Bacilli	Staphylococcales	Staphylococcaceae	Staphylococcus	NA
IL.5	ASV_5oo_dg6	0,084284701	0,481332738	0,944745229	Bacteria	Actinobacteriota	Actinobacteria	Micrococcales	Micrococcaceae	Rothia	mucilaginosa
IL.5	ASV_5r4_ie2	-0,125515996	0,528151292	0,943044334	Bacteria	Actinobacteriota	Actinobacteria	Corynebacteriales	Corynebacteriaceae	Corynebacterium	NA
IL.5	ASV_5xz_bqw	0,000677245	0,510127193	0,944197445	Bacteria	Firmicutes	Bacilli	Lactobacillales	Lactobacillaceae	Liquorilactobacillus	NA
IL.5	ASV_66n_hd0	0,167698658	0,529064993	0,950277977	Bacteria	Firmicutes	Bacilli	Staphylococcales	Staphylococcaceae	Staphylococcus	aureus
IL.5	ASV_6dr_o2s	-0,022074948	0,520734635	0,937293589	Bacteria	Firmicutes	Bacilli	Staphylococcales	Staphylococcaceae	Staphylococcus	NA
IL.5	ASV_6ut_tpw	0,009658798	0,568520345	0,951352243	Bacteria	Proteobacteria	Gammaproteobacteria	Burkholderiales	Comamonadaceae	Tepidimonas	NA
IL.5	ASV_727_4zo	-0,26814048	0,356524861	0,903800877	Bacteria	Firmicutes	Bacilli	Lactobacillales	Carnobacteriaceae	Dolosigranulum	NA
IL.5	ASV_79g_5uy	-0,034378225	0,510945145	0,940529761	Bacteria	Firmicutes	Clostridia	Lachnospirales	Lachnospiraceae	Roseburia	NA
IL.5	ASV_7cr_wom	-0,096571852	0,540808983	0,937087674	Bacteria	Firmicutes	Bacilli	Staphylococcales	Staphylococcaceae	Staphylococcus	NA
IL.5	ASV_7jt_wq3	-0,016802116	0,594250819	0,957949654	Bacteria	Actinobacteriota	Actinobacteria	Corynebacteriales	Corynebacteriaceae	Corynebacterium	NA
IL.5	ASV_7ka_s2c	-0,187306502	0,455598316	0,932693544	Bacteria	Actinobacteriota	Actinobacteria	Corynebacteriales	Corynebacteriaceae	Corynebacterium	NA
IL.5	ASV_7x9_tvb	0,194869066	0,442465667	0,945277017	Bacteria	Firmicutes	Clostridia	Peptostreptococcales-Tissierellales	Family XI	Peptoniphilus	gorbachii/lacydonensis/rhinitidis
IL.5	ASV_873_9b5	0,18753225	0,458294212	0,929922317	Bacteria	Firmicutes	Bacilli	Staphylococcales	Staphylococcaceae	NA	NA
IL.5	ASV_8lf_xek	-0,054776187	0,51358289	0,943607608	Bacteria	Bacteroidota	Bacteroidia	Bacteroidales	Prevotellaceae	Prevotella_9	copri
IL.5	ASV_8lv_u9v	0,06190338	0,520328838	0,940771155	Bacteria	Firmicutes	Bacilli	Lactobacillales	Streptococcaceae	Streptococcus	NA
IL.5	ASV_8pb_fin	-0,040247678	0,564512304	0,949543093	Bacteria	Bacteroidota	Bacteroidia	Cytophagales	Hymenobacteraceae	Hymenobacter	NA
IL.5	ASV_8re_um4	-0,367356811	0,196270705	0,872246773	Bacteria	Firmicutes	Bacilli	Staphylococcales	Staphylococcaceae	Staphylococcus	NA

Variable	FeatureID	spearman.erho	spearman.ep	spearman.eBH	Domain	Phylum	Class	Order	Family	Genus	Species
IL.5	ASV_8v2_sgp	-0,284861971	0,313584424	0,903187362	Bacteria	Actinobacteriota	Actinobacteria	Corynebacteriales	Corynebacteriaceae	Corynebacterium	NA
IL.5	ASV_8zu_e97	0,101618937	0,510834054	0,939846775	Bacteria	Firmicutes	Bacilli	Staphylococcales	Staphylococcaceae	Staphylococcus	NA
IL.5	ASV_926_7zf	0,037022704	0,680773713	0,962792733	Bacteria	Firmicutes	Bacilli	Staphylococcales	Staphylococcaceae	Staphylococcus	NA
IL.5	ASV_9cd_tui	-0,101973684	0,491567852	0,946920539	Bacteria	Firmicutes	Bacilli	Lactobacillales	Enterococcaceae	Tetragenococcus	NA
IL.5	ASV_9d0_o06	0,25744969	0,357713908	0,915668159	Bacteria	Firmicutes	Negativicutes	Veillonellales-Selenomonadales	Veillonellaceae	Negativicoccus	NA
IL.5	ASV_9eq_kr6	-0,130708204	0,474643713	0,930532942	Bacteria	Firmicutes	Bacilli	Lactobacillales	Streptococcaceae	Streptococcus	mitis/parasanguinis
IL.5	ASV_9jb_2ek	-0,308597781	0,277204244	0,904609739	Bacteria	Actinobacteriota	Actinobacteria	Corynebacteriales	Corynebacteriaceae	Corynebacterium	NA
IL.5	ASV_9kg_f3f	-0,049100232	0,53536204	0,942731556	Bacteria	Actinobacteriota	Actinobacteria	Corynebacteriales	NA	NA	NA
IL.5	ASV_9l0_247	0,08431695	0,538232617	0,948049549	Bacteria	Firmicutes	Bacilli	Staphylococcales	Gemellaceae	Gemella	morbillorum
IL.5	ASV_9mx_kho	0,082252967	0,530358621	0,943448764	Bacteria	Actinobacteriota	Actinobacteria	Frankiales	Sporichthyaceae	NA	NA
IL.5	ASV_9o6_1ja	0,195094814	0,461267012	0,941718552	Bacteria	Firmicutes	Bacilli	Staphylococcales	Staphylococcaceae	Staphylococcus	aureus
IL.5	ASV_9vy_jj2	-0,05442144	0,579735584	0,951973674	Bacteria	Firmicutes	Bacilli	Staphylococcales	Staphylococcaceae	Staphylococcus	NA
IL.5	ASV_9wz_9u7	0,017801858	0,555462425	0,949450483	Bacteria	Actinobacteriota	Actinobacteria	NA	NA	NA	NA
IL.5	ASV_aew_fyi	0,082365841	0,529457508	0,953068734	Bacteria	Actinobacteriota	Actinobacteria	Corynebacteriales	Corynebacteriaceae	Corynebacterium	mucifaciens
IL.5	ASV_ajo_fen	0,15747549	0,521531755	0,949130447	Bacteria	Actinobacteriota	Actinobacteria	Corynebacteriales	Corynebacteriaceae	Corynebacterium	NA
IL.5	ASV_aqj_vcn	-0,124161507	0,542650844	0,947730071	Bacteria	Firmicutes	Bacilli	Lactobacillales	Streptococcaceae	Streptococcus	mitis/phage/pneumoniae/pseudopneumoniae
IL.5	ASV_arl_xwg	-0,03002451	0,553224555	0,949003214	Bacteria	Firmicutes	Clostridia	Lachnospirales	Lachnospiraceae	Blautia	NA
IL.5	ASV_aw8_q96	0,127854102	0,577070054	0,959194631	Bacteria	Actinobacteriota	Actinobacteria	Corynebacteriales	Corynebacteriaceae	Corynebacterium	NA
IL.5	ASV_bg2_zbh	-0,102796053	0,517570971	0,933517837	Bacteria	Firmicutes	Bacilli	Staphylococcales	Staphylococcaceae	Staphylococcus	NA
IL.5	ASV_bh3_94k	-0,004740712	0,565831567	0,950348121	Bacteria	Firmicutes	Bacilli	Lactobacillales	Streptococcaceae	Lactococcus	NA
IL.5	ASV_bnk_uhh	0,23308501	0,391288452	0,93924262	Bacteria	Firmicutes	Clostridia	Peptostreptococcales-Tissierellales	Family XI	Anaerococcus	provencensis
IL.5	ASV_bsd_ayc	0,00248323	0,548906315	0,943325359	Bacteria	Planctomycetota	Planctomycetes	Pirellulales	Pirellulaceae	NA	NA
IL.5	ASV_btl_vxl	0,218991873	0,430470291	0,920053561	Bacteria	Firmicutes	Clostridia	Peptostreptococcales-Tissierellales	Family XI	Anaerococcus	vaginalis
IL.5	ASV_bwx_wef	0,246130031	0,381144355	0,911271941	Bacteria	Actinobacteriota	Actinobacteria	Corynebacteriales	Corynebacteriaceae	Corynebacterium	amycolatum/jeikeium/lactis/urealyticum/vitaeruminis
IL.5	ASV_c46_jpb	-0,273913184	0,338292316	0,908863169	Bacteria	Actinobacteriota	Actinobacteria	Corynebacteriales	Corynebacteriaceae	Corynebacterium	NA
IL.5	ASV_c4o_xms	0,290908798	0,308953799	0,9105934	Bacteria	Firmicutes	Bacilli	Staphylococcales	Staphylococcaceae	NA	NA
IL.5	ASV_cep_rux	0,194159572	0,440212723	0,938405527	Bacteria	Firmicutes	Clostridia	Peptostreptococcales-Tissierellales	Family XI	Anaerococcus	NA
IL.5	ASV_cje_lx4	0,129595588	0,489044319	0,933953849	Bacteria	Proteobacteria	Gammaproteobacteria	Burkholderiales	Neisseriaceae	NA	NA
IL.5	ASV_co0_sbi	-0,143995098	0,476162585	0,93703189	Bacteria	Actinobacteriota	Actinobacteria	Corynebacteriales	Corynebacteriaceae	Corynebacterium	NA
IL.5	ASV_cqp_otd	-0,023203689	0,52622135	0,934794205	Bacteria	Firmicutes	Negativicutes	Veillonellales-Selenomonadales	Veillonellaceae	Negativicoccus	NA

Variable	FeatureID	spearman.erho	spearman.ep	spearman.eBH	Domain	Phylum	Class	Order	Family	Genus	Species
IL.5	ASV_cy2_emp	0,108939628	0,522939281	0,938536751	Bacteria	Actinobacteriota	Actinobacteria	Corynebacteriales	Corynebacteriaceae	Corynebacterium	NA
IL.5	ASV_d0q_oe1	-0,038602941	0,519447765	0,94713332	Bacteria	Bacteroidota	Bacteroidia	Bacteroidales	Prevotellaceae	Prevotella_9	NA
IL.5	ASV_da2_u30	0,205737229	0,427418012	0,928638992	Bacteria	Bacteroidota	Bacteroidia	Bacteroidales	Prevotellaceae	Prevotella	buccalis
IL.5	ASV_dhe_20n	0,183807405	0,480102286	0,946152491	Bacteria	Firmicutes	Bacilli	Staphylococcales	Staphylococcaceae	Staphylococcus	aureus
IL.5	ASV_dlu_bk7	-0,007240067	0,627536698	0,956869952	Bacteria	Proteobacteria	Gammaproteobacteria	Burkholderiales	Neisseriaceae	NA	NA
IL.5	ASV_dmt_o1x	0,071336429	0,58250499	0,951997634	Bacteria	Proteobacteria	Gammaproteobacteria	Enterobacterales	Pasteurellaceae	Haemophilus	haemolyticus/influenzae
IL.5	ASV_dui_4x1	-0,278831269	0,322809804	0,89304925	Bacteria	Firmicutes	Bacilli	Lactobacillales	Carnobacteriaceae	Dolosigranulum	NA
IL.5	ASV_dz0_fy2	-0,037748323	0,56073128	0,952330917	Bacteria	Actinobacteriota	Actinobacteria	Corynebacteriales	Corynebacteriaceae	Corynebacterium	NA
IL.5	ASV_e7g_uqv	-0,143285604	0,466845732	0,924210438	Bacteria	Actinobacteriota	Thermoleophilia	Gaiellales	NA	NA	NA
IL.5	ASV_e8x_8cs	0,013464267	0,765361222	0,973475679	Bacteria	Actinobacteriota	Actinobacteria	Corynebacteriales	Corynebacteriaceae	Lawsonella	NA
IL.5	ASV_elz_ffm	-0,136706656	0,490111461	0,940348711	Bacteria	Actinobacteriota	Actinobacteria	Corynebacteriales	Corynebacteriaceae	Corynebacterium	NA
IL.5	ASV_f1j_s2e	0,180534056	0,480166382	0,938622552	Bacteria	Actinobacteriota	Actinobacteria	Corynebacteriales	Corynebacteriaceae	Corynebacterium	aurimucosum/simulans/striatum/xerosis
IL.5	ASV_f6c_v0m	-0,241905315	0,396795085	0,918409764	Bacteria	Actinobacteriota	Actinobacteria	Corynebacteriales	Corynebacteriaceae	Corynebacterium	NA
IL.5	ASV_f8c_ir4	-0,077286507	0,496444923	0,936312543	Bacteria	Bacteroidota	Bacteroidia	Bacteroidales	Bacteroidaceae	Bacteroides	plebeius
IL.5	ASV_fb6_dje	-0,089041538	0,559915446	0,952624973	Bacteria	Firmicutes	Bacilli	Staphylococcales	Staphylococcaceae	Staphylococcus	aureus/devriesei/epidermidis/haemolyticus/hominis
IL.5	ASV_fgq_ohv	0,156362874	0,51174425	0,942642972	Bacteria	Actinobacteriota	Actinobacteria	Corynebacteriales	Corynebacteriaceae	Corynebacterium	NA
IL.5	ASV_fmb_men	-0,06378999	0,546625686	0,947561303	Bacteria	Actinobacteriota	Actinobacteria	Corynebacteriales	Corynebacteriaceae	Corynebacterium	NA
IL.5	ASV_fqm_8pm	0,276509288	0,323108352	0,919513795	Bacteria	Firmicutes	Bacilli	Lactobacillales	Streptococcaceae	Streptococcus	anginosus/cristatus/infantis/mitis/oralis/pneumoniae/pseudopneumoniae/sanguinis/timonensis
IL.5	ASV_fug_ldj	-0,099135707	0,483870721	0,93400561	Bacteria	Bacteroidota	Bacteroidia	Bacteroidales	Prevotellaceae	Prevotella_9	NA
IL.5	ASV_g3d_cs4	-0,027847652	0,58555257	0,951359806	Bacteria	Proteobacteria	Gammaproteobacteria	Enterobacterales	Pasteurellaceae	Haemophilus	influenzae
IL.5	ASV_geq_907	0,250064499	0,326608767	0,933549711	Bacteria	Firmicutes	Clostridia	Peptostreptococcales-Tissierellales	Family XI	Anaerococcus	octavius
IL.5	ASV_gg8_mtq	-0,109536249	0,474700636	0,937593866	Bacteria	Bacteroidota	Bacteroidia	Bacteroidales	Muribaculaceae	NA	NA
IL.5	ASV_gzb_mis	-0,027073658	0,551249129	0,948385608	Bacteria	Firmicutes	Clostridia	Lachnospirales	Lachnospiraceae	Coprococcus	catus
IL.5	ASV_h1r_zyt	0,178276574	0,468534329	0,930417989	Bacteria	Fusobacteriota	Fusobacteriia	Fusobacteriales	Fusobacteriaceae	Fusobacterium	NA
IL.5	ASV_h40_0md	-0,082349716	0,581835411	0,953238021	Bacteria	Actinobacteriota	Actinobacteria	Corynebacteriales	Corynebacteriaceae	Corynebacterium	NA
IL.5	ASV_h4w_6wi	0,063193369	0,55656279	0,946761065	Bacteria	Firmicutes	Bacilli	Staphylococcales	Staphylococcaceae	NA	NA
IL.5	ASV_hbi_ebs	0,131433824	0,489717008	0,934461601	Bacteria	Actinobacteriota	Actinobacteria	Corynebacteriales	Corynebacteriaceae	Lawsonella	clevelandensis
IL.5	ASV_het_bu3	0,141560243	0,489781231	0,942048758	Bacteria	Campylobacterota	Campylobacteria	Campylobacterales	Campylobacteraceae	Campylobacter	NA
IL.5	ASV_hjn_92c	0,193046956	0,458451548	0,944793171	Bacteria	Firmicutes	Clostridia	Peptostreptococcales-Tissierellales	Family XI	Finegoldia	magna
IL.5	ASV_hkw_6s3	0,146333204	0,523657125	0,935146366	Bacteria	Actinobacteriota	Actinobacteria	Corynebacteriales	Corynebacteriaceae	Corynebacterium	NA
IL.5	ASV_hpm_svr	0,030524381	0,610907103	0,956064427	Bacteria	Actinobacteriota	Actinobacteria	Corynebacteriales	Corynebacteriaceae	Corynebacterium	NA
IL.5	ASV_hq8_93x	0,083333333	0,555497566	0,955729665	Bacteria	Actinobacteriota	Actinobacteria	Corynebacteriales	Corynebacteriaceae	Corynebacterium	NA

Variable	FeatureID	spearman.erho	spearman.ep	spearman.eBH	Domain	Phylum	Class	Order	Family	Genus	Species
IL.5	ASV_hqq_2ph	0,003918344	0,521336739	0,949030828	Bacteria	Proteobacteria	Gammaproteobacteria	Burkholderiales	Oxalobacteraceae	Massilia	timonae
IL.5	ASV_i0i_mxo	0,003337848	0,577897713	0,949285156	Bacteria	Actinobacteriota	Actinobacteria	Corynebacteriales	Corynebacteriaceae	Corynebacterium	pyruviciproducens
IL.5	ASV_i35_0op	0,221426729	0,405543499	0,939165627	Bacteria	Actinobacteriota	Actinobacteria	Corynebacteriales	Corynebacteriaceae	Corynebacterium	aurimucosum/jeikeium/propinquum/pseudodiphtheriticum
IL.5	ASV_i88_mm0	-0,002015609	0,538002627	0,938028252	Bacteria	Actinobacteriota	Actinobacteria	Corynebacteriales	Corynebacteriaceae	Corynebacterium	NA
IL.5	ASV_ic5_nai	0,088444917	0,533863274	0,945632779	Bacteria	Firmicutes	Bacilli	Lactobacillales	Streptococcaceae	Streptococcus	sobrinus
IL.5	ASV_igm_m49	-0,129321465	0,505595653	0,934470605	Bacteria	NA	NA	NA	NA	NA	NA
IL.5	ASV_ii5_tjz	0,048874484	0,584186996	0,95425508	Bacteria	Firmicutes	Bacilli	Staphylococcales	Staphylococcaceae	Staphylococcus	NA
IL.5	ASV_ikg_ra0	0,030395382	0,700587267	0,966400527	Bacteria	Actinobacteriota	Actinobacteria	Corynebacteriales	Corynebacteriaceae	Corynebacterium	NA
IL.5	ASV_iov_l70	-0,252144608	0,380738007	0,910935266	Bacteria	Firmicutes	Bacilli	Lactobacillales	Carnobacteriaceae	Dolosigranulum	pigrum
IL.5	ASV_iqo_ea7	-0,065337977	0,525560888	0,944748655	Bacteria	Firmicutes	Bacilli	Staphylococcales	Staphylococcaceae	Staphylococcus	NA
IL.5	ASV_jjw_h6c	-0,20994582	0,407474949	0,941129724	Bacteria	Actinobacteriota	Actinobacteria	Corynebacteriales	Corynebacteriaceae	Corynebacterium	NA
IL.5	ASV_jl5_6pn	0,011400284	0,696214381	0,965699985	Bacteria	Firmicutes	Bacilli	Staphylococcales	Staphylococcaceae	Staphylococcus	aureus
IL.5	ASV_jp2_wik	0,018946723	0,782050961	0,973113461	Bacteria	Firmicutes	Bacilli	Staphylococcales	Staphylococcaceae	Staphylococcus	epidermidis
IL.5	ASV_jph_cup	0,129982585	0,615500216	0,957625059	Bacteria	Actinobacteriota	Actinobacteria	Corynebacteriales	Corynebacteriaceae	Corynebacterium	NA
IL.5	ASV_jy3_f61	-0,103344298	0,510233368	0,937276161	Bacteria	Actinobacteriota	Actinobacteria	Corynebacteriales	Corynebacteriaceae	Corynebacterium	NA
IL.5	ASV_jy9_jqd	-0,063257869	0,509493229	0,946090602	Bacteria	Firmicutes	Clostridia	Lachnospirales	Lachnospiraceae	[Eubacterium] ventriosum group	NA
IL.5	ASV_k2s_cqm	-0,177679954	0,495552169	0,937533342	Bacteria	Actinobacteriota	Actinobacteria	Corynebacteriales	Corynebacteriaceae	Corynebacterium	NA
IL.5	ASV_k3h_3z5	-0,057211042	0,508502991	0,946395308	Bacteria	Proteobacteria	Gammaproteobacteria	Xanthomonadales	Xanthomonadaceae	Lysobacter	NA
IL.5	ASV_k3h_qcz	-0,142253612	0,456364932	0,93492885	Bacteria	Firmicutes	Bacilli	Staphylococcales	Staphylococcaceae	Staphylococcus	NA
IL.5	ASV_kaj_b2n	0,015431502	0,545056358	0,944060369	Bacteria	Firmicutes	Bacilli	Lactobacillales	P5D1-392	NA	NA
IL.5	ASV_kcs_cbi	-0,067240712	0,526284167	0,946630254	Bacteria	Actinobacteriota	Actinobacteria	Corynebacteriales	Corynebacteriaceae	Corynebacterium	NA
IL.5	ASV_ki3_dfk	-0,07997936	0,558267446	0,951900066	Bacteria	Actinobacteriota	Actinobacteria	Corynebacteriales	Corynebacteriaceae	Corynebacterium	NA
IL.5	ASV_kkl_mxy	0,308307534	0,271251477	0,908026648	Bacteria	Actinobacteriota	Actinobacteria	Corynebacteriales	Corynebacteriaceae	Corynebacterium	kroppenstedtii
IL.5	ASV_kru_fff	-0,163151445	0,479118036	0,940263168	Bacteria	Proteobacteria	Gammaproteobacteria	Enterobacterales	Erwiniaceae	NA	NA
IL.5	ASV_ktj_lyl	0,111438983	0,491851118	0,942041497	Bacteria	Proteobacteria	Gammaproteobacteria	Burkholderiales	Neisseriaceae	NA	NA
IL.5	ASV_l3n_w4d	0,155185759	0,45471738	0,933363427	Bacteria	Synergistota	Synergistia	Synergistales	Synergistaceae	Jonquetella	anthropi
IL.5	ASV_lky_mqj	0,105343782	0,503030125	0,939724635	Bacteria	Firmicutes	Bacilli	Lactobacillales	Streptococcaceae	Streptococcus	NA
IL.5	ASV_lmq_jff	-0,265753999	0,384042181	0,890657872	Bacteria	Firmicutes	Bacilli	Lactobacillales	Carnobacteriaceae	Dolosigranulum	NA
IL.5	ASV_lnh_9fs	-0,055163184	0,535753507	0,946897715	Bacteria	Actinobacteriota	Actinobacteria	Corynebacteriales	Corynebacteriaceae	Corynebacterium	afermentans/coyleae
IL.5	ASV_lvd_sr9	-0,355988777	0,203693059	0,886901879	Bacteria	Actinobacteriota	Actinobacteria	Corynebacteriales	Corynebacteriaceae	Corynebacterium	NA
IL.5	ASV_lvh_wn0	-0,047600619	0,639306572	0,963495393	Bacteria	Actinobacteriota	Actinobacteria	Micrococcales	Dermabacteraceae	Dermabacter	jinjuensis
IL.5	ASV_m52_gpv	0,069578818	0,514776338	0,932936955	Bacteria	Actinobacteriota	Actinobacteria	Corynebacteriales	Corynebacteriaceae	Corynebacterium	NA

Variable	FeatureID	spearman.erho	spearman.ep	spearman.eBH	Domain	Phylum	Class	Order	Family	Genus	Species
IL.5	ASV_mbn_3b6	-0,014673633	0,551913434	0,948102432	Bacteria	Firmicutes	Clostridia	Lachnospirales	Lachnospiraceae	[Ruminococcus] torques group	NA
IL.5	ASV_mcc_ype	0,028976393	0,586949975	0,95190729	Bacteria	Firmicutes	Bacilli	Staphylococcales	Staphylococcaceae	Staphylococcus	epidermidis
IL.5	ASV_mdt_jke	-0,025557921	0,488808318	0,936878539	Bacteria	Proteobacteria	Gammaproteobacteria	Enterobacterales	Pasteurellaceae	Haemophilus	haemolyticus/influenzae
IL.5	ASV_mgr_u9o	-0,175390222	0,451551693	0,928485385	Bacteria	Actinobacteriota	Actinobacteria	Corynebacteriales	Corynebacteriaceae	Corynebacterium	NA
IL.5	ASV_mlb_2sd	-0,049003483	0,561377304	0,953018154	Bacteria	Firmicutes	Bacilli	Staphylococcales	Staphylococcaceae	Staphylococcus	NA
IL.5	ASV_mm9_js5	-0,098168215	0,614209521	0,958692207	Bacteria	Firmicutes	Bacilli	Staphylococcales	Staphylococcaceae	Staphylococcus	aureus
IL.5	ASV_mvn_oem	-0,154073142	0,46201124	0,922913513	Bacteria	Actinobacteriota	Actinobacteria	Corynebacteriales	Corynebacteriaceae	Corynebacterium	NA
IL.5	ASV_n1a_pw6	0,080995227	0,547313305	0,945928385	Bacteria	Proteobacteria	Alphaproteobacteria	Rhizobiales	Methyloligellaceae	NA	NA
IL.5	ASV_n5j_9sq	0,05253483	0,551605585	0,950094886	Bacteria	Firmicutes	Bacilli	Staphylococcales	Staphylococcaceae	Staphylococcus	NA
IL.5	ASV_n79_65k	0,077770253	0,56796746	0,944948846	Bacteria	Proteobacteria	Gammaproteobacteria	Burkholderiales	Neisseriaceae	NA	NA
IL.5	ASV_nbq_6pg	-0,080205108	0,507996683	0,939762471	Bacteria	Firmicutes	Clostridia	Peptostreptococcales-Tissierellales	Family XI	Anaerococcus	prevotii/tetradius
IL.5	ASV_nk5_hwz	0,07256192	0,531987354	0,947742206	Bacteria	Bacteroidota	Bacteroidia	Flavobacteriales	Weeksellaceae	Cloacibacterium	normanense
IL.5	ASV_ntr_hh5	-0,034781347	0,530513775	0,949572754	Bacteria	Proteobacteria	Gammaproteobacteria	Enterobacterales	Pasteurellaceae	Haemophilus	haemolyticus/influenzae/parainfluenzae
IL.5	ASV_nvc_oao	0,035490841	0,6587118	0,963349488	Bacteria	Deinococcota	Deinococci	Thermales	Thermaceae	Thermus	parvatiensis/thermophilus
IL.5	ASV_o0r_len	0,138157895	0,593118018	0,957346224	Bacteria	Firmicutes	Bacilli	Staphylococcales	Staphylococcaceae	Staphylococcus	aureus/capitis/caprae/epidermidis/haemolyticus/saprophyticus/warneri
IL.5	ASV_o10_2p1	-0,10381192	0,546402935	0,938854855	Bacteria	Proteobacteria	Gammaproteobacteria	Pseudomonadales	Pseudomonadaceae	Pseudomonas	brassicacearum/chlororaphis/corrugata/fluorescens/jessenii/kilonensis/mohnii/putida/synxantha
IL.5	ASV_o1f_10k	-0,053550697	0,511254613	0,941590783	Bacteria	Proteobacteria	Gammaproteobacteria	Burkholderiales	Comamonadaceae	NA	NA
IL.5	ASV_ode_i48	0,097136223	0,503477754	0,917151953	Bacteria	Actinobacteriota	Actinobacteria	Corynebacteriales	Corynebacteriaceae	Corynebacterium	NA
IL.5	ASV_on0_uxs	0,200158024	0,44718296	0,935793751	Bacteria	Actinobacteriota	Actinobacteria	Corynebacteriales	Corynebacteriaceae	Corynebacterium	afermentans/ihumii
IL.5	ASV_onw_luq	0,194611068	0,420469543	0,936203166	Bacteria	Proteobacteria	Gammaproteobacteria	Burkholderiales	Neisseriaceae	Eikenella	NA
IL.5	ASV_ouf_s4v	-0,151380289	0,506661512	0,935469609	Bacteria	Actinobacteriota	Actinobacteria	Corynebacteriales	Corynebacteriaceae	Corynebacterium	NA
IL.5	ASV_oyd_63r	-0,211703431	0,453165509	0,924057337	Bacteria	Firmicutes	Bacilli	Staphylococcales	Staphylococcaceae	Staphylococcus	NA
IL.5	ASV_p9u_72k	0,086284185	0,489648955	0,939985435	Bacteria	Firmicutes	Clostridia	Peptococcales	Peptococcaceae	Peptococcus	NA
IL.5	ASV_paz_uyl	0,022139448	0,764394057	0,971080613	Bacteria	Actinobacteriota	Actinobacteria	Propionibacteriales	Propionibacteriaceae	Cutibacterium	acnes/avidum
IL.5	ASV_pix_o0d	-0,014899381	0,530987499	0,948572834	Bacteria	Desulfobacterota	Desulfovibrionia	Desulfovibrionales	Desulfohalobiaceae	Desulfovermiculus	NA
IL.5	ASV_pjp_yzq	-0,130530831	0,478559239	0,930729056	Bacteria	Firmicutes	Bacilli	Staphylococcales	Staphylococcaceae	Staphylococcus	NA
IL.5	ASV_pni_ryk	0,022736068	0,506599513	0,940313094	Bacteria	Actinobacteriota	Actinobacteria	Corynebacteriales	Corynebacteriaceae	Corynebacterium	NA
IL.5	ASV_pwy_pjt	0,022590944	0,633776816	0,961387671	Bacteria	Actinobacteriota	Actinobacteria	Corynebacteriales	Corynebacteriaceae	Corynebacterium	NA
IL.5	ASV_q1f_0em	-0,043633901	0,633627948	0,959017396	Bacteria	Actinobacteriota	Actinobacteria	Corynebacteriales	Corynebacteriaceae	Corynebacterium	NA
IL.5	ASV_q1k_ad2	0,179179567	0,455647804	0,94338797	Bacteria	Firmicutes	Bacilli	Staphylococcales	Staphylococcaceae	Staphylococcus	NA
IL.5	ASV_q1o_24k	-0,325448271	0,193207488	0,921622647	Bacteria	Actinobacteriota	Actinobacteria	Corynebacteriales	Corynebacteriaceae	Corynebacterium	accolens/fastidiosum
IL.5	ASV_q1r_s4c	0,106069401	0,471930625	0,931195995	Bacteria	Actinobacteriota	Actinobacteria	Actinomycetales	Actinomycetaceae	Actinotignum	schaalii/timonense

Variable	FeatureID	spearman.erho	spearman.ep	spearman.eBH	Domain	Phylum	Class	Order	Family	Genus	Species
IL.5	ASV_qc8_76v	-0,08818692	0,519890114	0,947264944	Bacteria	Proteobacteria	Gammaproteobacteria	Enterobacterales	Pasteurellaceae	Haemophilus	influenzae
IL.5	ASV_qft_24l	0,168746775	0,492287472	0,92900294	Bacteria	Bacteroidota	Bacteroidia	Bacteroidales	Prevotellaceae	Alloprevotella	NA
IL.5	ASV_qis_xxl	-0,013045021	0,548028557	0,948331708	Bacteria	Firmicutes	Clostridia	Peptostreptococcales-Tissierellales	Family XI	Anaerococcus	nagyae
IL.5	ASV_qk1_qy8	0,053050826	0,520678979	0,94027579	Bacteria	Proteobacteria	Gammaproteobacteria	Enterobacterales	Pasteurellaceae	Lonepinella	NA
IL.5	ASV_r73_72u	-0,097361971	0,510064863	0,929953791	Bacteria	Actinobacteriota	Actinobacteria	Corynebacteriales	Corynebacteriaceae	Corynebacterium	NA
IL.5	ASV_r8p_k1e	0,11380934	0,622353008	0,958329516	Bacteria	Firmicutes	Bacilli	Staphylococcales	Staphylococcaceae	Staphylococcus	aureus
IL.5	ASV_r9n_48y	0,109552374	0,52403452	0,945970204	Bacteria	Firmicutes	Bacilli	Staphylococcales	Staphylococcaceae	Staphylococcus	NA
IL.5	ASV_rad_25g	-0,031072626	0,547657529	0,944859105	Bacteria	Proteobacteria	Gammaproteobacteria	Enterobacterales	Pasteurellaceae	NA	NA
IL.5	ASV_raj_rv1	-0,190370227	0,453254527	0,946498719	Bacteria	Firmicutes	Bacilli	Staphylococcales	Staphylococcaceae	Staphylococcus	aureus/capitis/caprae/epidermidis/haemolyticus/warneri
IL.5	ASV_rmx_t9x	-0,048342363	0,627777045	0,96178201	Bacteria	Firmicutes	Bacilli	Staphylococcales	Staphylococcaceae	Staphylococcus	aureus
IL.5	ASV_rs1_k0s	0,083881579	0,560075205	0,941044689	Bacteria	Firmicutes	Bacilli	Staphylococcales	Staphylococcaceae	Staphylococcus	NA
IL.5	ASV_rtx_tpz	-0,01370614	0,533867825	0,944734512	Bacteria	Proteobacteria	Gammaproteobacteria	Pseudomonadales	Moraxellaceae	Moraxella	catarrhalis/nonliquefaciens
IL.5	ASV_s8r_baf	0,047261997	0,719427947	0,966866549	Bacteria	Firmicutes	Clostridia	Peptostreptococcales-Tissierellales	Family XI	Anaerococcus	NA
IL.5	ASV_sjv_qdw	-0,095991357	0,558671853	0,947497569	Bacteria	Actinobacteriota	Actinobacteria	Corynebacteriales	Corynebacteriaceae	Corynebacterium	NA
IL.5	ASV_snw_j03	-0,047858617	0,520197774	0,944796694	Bacteria	Firmicutes	Bacilli	Lactobacillales	Streptococcaceae	Streptococcus	NA
IL.5	ASV_ssr_vha	0,015496001	0,689083704	0,963328971	Bacteria	Firmicutes	Bacilli	Staphylococcales	Staphylococcaceae	Staphylococcus	aureus
IL.5	ASV_t7p_n06	0,231940144	0,424407923	0,925395066	Bacteria	Firmicutes	Clostridia	Peptostreptococcales-Tissierellales	Family XI	Anaerococcus	NA
IL.5	ASV_t8l_ysz	0,368437178	0,184947553	0,886704603	Bacteria	Firmicutes	Bacilli	Staphylococcales	Staphylococcaceae	NA	NA
IL.5	ASV_ten_mjo	-0,120372162	0,488987427	0,936062482	Bacteria	Actinobacteriota	Actinobacteria	Corynebacteriales	Corynebacteriaceae	Corynebacterium	NA
IL.5	ASV_tu2_xgy	0,06127451	0,633056407	0,957626226	Bacteria	Actinobacteriota	Actinobacteria	Corynebacteriales	Corynebacteriaceae	Corynebacterium	NA
IL.5	ASV_tvh_ar7	0,183646156	0,470485634	0,936665533	Bacteria	Firmicutes	Bacilli	Lactobacillales	Streptococcaceae	Streptococcus	anginosus/constellatus/intermedius/lutetiensis
IL.5	ASV_v7r_7sn	0,009384675	0,580589826	0,951480075	Bacteria	Proteobacteria	Gammaproteobacteria	Burkholderiales	Comamonadaceae	Rubrivivax	NA
IL.5	ASV_xex_kso	-0,147268447	0,475749606	0,935515902	Bacteria	Actinobacteriota	Actinobacteria	Micrococcales	Bogoriellaceae	Georgenia	NA
IL.13	ASV_17p_uuy	0,091333402	0,658828019	0,960377885	Bacteria	Actinobacteriota	Actinobacteria	Corynebacteriales	Corynebacteriaceae	Corynebacterium	NA
IL.13	ASV_19p_rjx	0,013854806	0,576286382	0,942388405	Bacteria	Bacteroidota	Bacteroidia	Bacteroidales	Prevotellaceae	Prevotella_9	NA
IL.13	ASV_1af_96k	-0,079984424	0,554058397	0,917862541	Bacteria	Firmicutes	Bacilli	Lactobacillales	Carnobacteriaceae	Dolosigranulum	NA
IL.13	ASV_1dc_ewy	0,131394322	0,511380837	0,922185687	Bacteria	Firmicutes	Bacilli	Lactobacillales	Carnobacteriaceae	Dolosigranulum	NA
IL.13	ASV_1kn_zel	0,153591111	0,527392921	0,930636795	Bacteria	Actinobacteriota	Actinobacteria	Corynebacteriales	Corynebacteriaceae	Lawsonella	NA
IL.13	ASV_1ob_3tg	-0,190952204	0,409023335	0,860181245	Bacteria	Firmicutes	Bacilli	Staphylococcales	Staphylococcaceae	Staphylococcus	NA
IL.13	ASV_1wh_81x	0,04546866	0,608909717	0,942708634	Bacteria	Firmicutes	Negativicutes	Veillonellales-Selenomonadales	Veillonellaceae	Negativicoccus	succinicivorans
IL.13	ASV_242_lzz	0,159871849	0,534949808	0,946844269	Bacteria	Actinobacteriota	Actinobacteria	Corynebacteriales	Corynebacteriaceae	Corynebacterium	aurimucosum/pseudogenitalium/tuberculostearicum
IL.13	ASV_2d3_euj	0,410099018	0,135132883	0,767671847	Bacteria	Actinobacteriota	Actinobacteria	Corynebacteriales	Corynebacteriaceae	Corynebacterium	NA

Variable	FeatureID	spearman.erho	spearman.ep	spearman.eBH	Domain	Phylum	Class	Order	Family	Genus	Species
IL.13	ASV_2db_0um	-0,038468507	0,556471658	0,925286934	Bacteria	Firmicutes	Negativicutes	Veillonellales-Selenomonadales	Veillonellaceae	Dialister	NA
IL.13	ASV_2ee_ogi	-0,200951267	0,437985725	0,905824258	Bacteria	Firmicutes	Bacilli	Staphylococcales	Staphylococcaceae	Staphylococcus	lugdunensis
IL.13	ASV_2o1_p31	-0,027054863	0,525584586	0,930568218	Bacteria	Firmicutes	Clostridia	Lachnospirales	Lachnospiraceae	Lachnospiraceae AC2044 group	bacterium
IL.13	ASV_2x6_j18	-0,045848576	0,524111313	0,923566136	Bacteria	Firmicutes	Clostridia	Peptostreptococcales-Tissierellales	Peptostreptococcaceae	Criibacterium	NA
IL.13	ASV_2xa_4rw	-0,193910698	0,420464226	0,888974161	Bacteria	Bacteroidota	Bacteroidia	Bacteroidales	Prevotellaceae	Prevotella_9	NA
IL.13	ASV_2xm_w96	0,015528052	0,557523732	0,940404609	Bacteria	Firmicutes	Clostridia	Lachnospirales	Lachnospiraceae	Dorea	formicigenerans
IL.13	ASV_30c_6sw	-0,154835942	0,476299428	0,901792861	Bacteria	Proteobacteria	Gammaproteobacteria	Burkholderiales	Oxalobacteraceae	Massilia	timonae
IL.13	ASV_339_mvy	-0,16930508	0,430157713	0,872488751	Bacteria	Proteobacteria	Gammaproteobacteria	Enterobacterales	Pasteurellaceae	Haemophilus	NA
IL.13	ASV_3em_n0d	-0,19433103	0,418161418	0,881688755	Bacteria	Firmicutes	Bacilli	Lactobacillales	Lactobacillaceae	Liquorilactobacillus	NA
IL.13	ASV_3en_7ow	-0,012424059	0,608744009	0,948285148	Bacteria	Firmicutes	Bacilli	Staphylococcales	Gemellaceae	Gemella	morbillorum
IL.13	ASV_3i5_y1w	-0,178107813	0,433974325	0,875428288	Bacteria	Proteobacteria	Gammaproteobacteria	Enterobacterales	Pasteurellaceae	Haemophilus	NA
IL.13	ASV_3kg_4bk	0,001099331	0,640758788	0,951956001	Bacteria	Firmicutes	Bacilli	Lactobacillales	Carnobacteriaceae	Dolosigranulum	pigrum
IL.13	ASV_3kh_nw9	0,278664279	0,335188138	0,854982778	Bacteria	Actinobacteriota	Actinobacteria	Corynebacteriales	Corynebacteriaceae	Corynebacterium	NA
IL.13	ASV_3pn_tj1	0,041289585	0,50537103	0,919721829	Bacteria	Actinobacteriota	Actinobacteria	Corynebacteriales	Corynebacteriaceae	Corynebacterium	NA
IL.13	ASV_3tk_6ez	-0,146122126	0,453071119	0,888000033	Bacteria	Actinobacteriota	Actinobacteria	Corynebacteriales	Corynebacteriaceae	Corynebacterium	NA
IL.13	ASV_440_o98	-0,074479685	0,473687844	0,90473323	Bacteria	Firmicutes	Clostridia	Peptostreptococcales-Tissierellales	Family XI	Anaerococcus	NA
IL.13	ASV_46z_g65	-0,003645576	0,570654728	0,937678186	Bacteria	Firmicutes	Negativicutes	Veillonellales-Selenomonadales	Veillonellaceae	Negativicoccus	NA
IL.13	ASV_47r_9ra	0,0169103	0,559464111	0,932994222	Bacteria	Actinobacteriota	Actinobacteria	Corynebacteriales	Corynebacteriaceae	Corynebacterium	NA
IL.13	ASV_49k_gjb	0,030175023	0,553566643	0,932286843	Bacteria	Firmicutes	Clostridia	Oscillospirales	Oscillospiraceae	UCG-002	NA
IL.13	ASV_4b4_zwy	0,010395146	0,537274376	0,934065286	Bacteria	Campylobacterota	Campylobacteria	Campylobacterales	Campylobacteraceae	Campylobacter	ureolyticus
IL.13	ASV_4cz_ka1	-0,151335866	0,475330851	0,895343239	Bacteria	Firmicutes	Bacilli	Staphylococcales	Staphylococcaceae	Staphylococcus	NA
IL.13	ASV_4kw_hck	-0,041265335	0,536721768	0,928252759	Bacteria	Firmicutes	Clostridia	Peptostreptococcales-Tissierellales	Family XI	Anaerococcus	NA
IL.13	ASV_4m8_kdu	0,003015077	0,577446352	0,943594541	Bacteria	Bacteroidota	Bacteroidia	Bacteroidales	Prevotellaceae	Prevotella_9	NA
IL.13	ASV_4ni_ro3	0,127077831	0,525757218	0,927368823	Bacteria	Actinobacteriota	Actinobacteria	Corynebacteriales	Corynebacteriaceae	Corynebacterium	NA
IL.13	ASV_4pz_tc7	-0,122130841	0,637854243	0,957388315	Bacteria	Firmicutes	Bacilli	Staphylococcales	Staphylococcaceae	Staphylococcus	argenteus/aureus/equorum/phage/schweitzeri/simiae
IL.13	ASV_4qr_yd2	-0,167308501	0,45181646	0,908170908	Bacteria	Firmicutes	Bacilli	Lactobacillales	Streptococcaceae	Streptococcus	NA
IL.13	ASV_4rd_dxc	0,4528921	0,107120253	0,715611737	Bacteria	Actinobacteriota	Actinobacteria	Corynebacteriales	Corynebacteriaceae	Corynebacterium	NA
IL.13	ASV_513_wtm	-0,119875595	0,479340254	0,916612307	Bacteria	Proteobacteria	Gammaproteobacteria	Enterobacterales	Enterobacteriaceae	Raoultella	NA
IL.13	ASV_53d_zfm	0,004866157	0,851944983	0,979593292	Bacteria	Actinobacteriota	Actinobacteria	Corynebacteriales	Corynebacteriaceae	Lawsonella	NA
IL.13	ASV_53v_62f	-0,178471562	0,49381037	0,939787833	Bacteria	Firmicutes	Bacilli	Staphylococcales	Staphylococcaceae	Staphylococcus	NA

Variable	FeatureID	spearman.erho	spearman.ep	spearman.eBH	Domain	Phylum	Class	Order	Family	Genus	Species
IL.13	ASV_566_wbp	0,258261987	0,378127296	0,867431778	Bacteria	Actinobacteriota	Actinobacteria	Corynebacteriales	Corynebacteriaceae	Corynebacterium	NA
IL.13	ASV_5do_e0k	-0,148522871	0,436131168	0,906339598	Bacteria	Actinobacteriota	Actinobacteria	Corynebacteriales	Corynebacteriaceae	Corynebacterium	NA
IL.13	ASV_5j9_202	0,105842956	0,587021547	0,948915397	Bacteria	Actinobacteriota	Actinobacteria	Corynebacteriales	Corynebacteriaceae	Corynebacterium	NA
IL.13	ASV_5mh_1v6	-0,190742037	0,450937568	0,899164156	Bacteria	Firmicutes	Bacilli	Staphylococcales	Staphylococcaceae	Staphylococcus	NA
IL.13	ASV_5oo_dg6	-0,280394109	0,31526344	0,865765635	Bacteria	Actinobacteriota	Actinobacteria	Micrococcales	Micrococcaceae	Rothia	mucilaginosa
IL.13	ASV_5r4_ie2	0,123812171	0,508550484	0,919151966	Bacteria	Actinobacteriota	Actinobacteria	Corynebacteriales	Corynebacteriaceae	Corynebacterium	NA
IL.13	ASV_5xz_bqw	-0,195082779	0,403764169	0,874558753	Bacteria	Firmicutes	Bacilli	Lactobacillales	Lactobacillaceae	Liquorilactobacillus	NA
IL.13	ASV_66n_hd0	0,021501624	0,70743848	0,96278901	Bacteria	Firmicutes	Bacilli	Staphylococcales	Staphylococcaceae	Staphylococcus	aureus
IL.13	ASV_6dr_o2s	-0,167995583	0,44966953	0,900519586	Bacteria	Firmicutes	Bacilli	Staphylococcales	Staphylococcaceae	Staphylococcus	NA
IL.13	ASV_6ut_tpw	-0,147455874	0,46429259	0,89529352	Bacteria	Proteobacteria	Gammaproteobacteria	Burkholderiales	Comamonadaceae	Tepidimonas	NA
IL.13	ASV_727_4zo	0,076379265	0,546304921	0,929756306	Bacteria	Firmicutes	Bacilli	Lactobacillales	Carnobacteriaceae	Dolosigranulum	NA
IL.13	ASV_79g_5uy	0,031330938	0,587256991	0,94173678	Bacteria	Firmicutes	Clostridia	Lachnospirales	Lachnospiraceae	Roseburia	NA
IL.13	ASV_7cr_wom	-0,060923962	0,591622895	0,944474412	Bacteria	Firmicutes	Bacilli	Staphylococcales	Staphylococcaceae	Staphylococcus	NA
IL.13	ASV_7jt_wq3	-0,087914159	0,590089665	0,939505721	Bacteria	Actinobacteriota	Actinobacteria	Corynebacteriales	Corynebacteriaceae	Corynebacterium	NA
IL.13	ASV_7ka_s2c	-0,000476916	0,582889005	0,933917292	Bacteria	Actinobacteriota	Actinobacteria	Corynebacteriales	Corynebacteriaceae	Corynebacterium	NA
IL.13	ASV_7x9_tvb	-0,007064819	0,884903894	0,981727269	Bacteria	Firmicutes	Clostridia	Peptostreptococcales-Tissierellales	Family XI	Peptoniphilus	gorbachii/lacydonensis/rhinitidis
IL.13	ASV_873_9b5	0,154132694	0,512450519	0,919791766	Bacteria	Firmicutes	Bacilli	Staphylococcales	Staphylococcaceae	NA	NA
IL.13	ASV_8lf_xek	0,008156067	0,593239527	0,941892567	Bacteria	Bacteroidota	Bacteroidia	Bacteroidales	Prevotellaceae	Prevotella_9	copri
IL.13	ASV_8lv_u9v	-0,089352989	0,489175637	0,912382281	Bacteria	Firmicutes	Bacilli	Lactobacillales	Streptococcaceae	Streptococcus	NA
IL.13	ASV_8pb_fin	0,01520472	0,578035372	0,938335421	Bacteria	Bacteroidota	Bacteroidia	Cytophagales	Hymenobacteraceae	Hymenobacter	NA
IL.13	ASV_8re_um4	0,134514483	0,553944171	0,940696337	Bacteria	Firmicutes	Bacilli	Staphylococcales	Staphylococcaceae	Staphylococcus	NA
IL.13	ASV_8v2_sgp	-0,045937492	0,61737498	0,949709144	Bacteria	Actinobacteriota	Actinobacteria	Corynebacteriales	Corynebacteriaceae	Corynebacterium	NA
IL.13	ASV_8zu_e97	0,052711312	0,54812917	0,934417473	Bacteria	Firmicutes	Bacilli	Staphylococcales	Staphylococcaceae	Staphylococcus	NA
IL.13	ASV_926_7zf	-0,044401662	0,65472909	0,961623091	Bacteria	Firmicutes	Bacilli	Staphylococcales	Staphylococcaceae	Staphylococcus	NA
IL.13	ASV_9cd_tui	-0,281849106	0,320566687	0,833211869	Bacteria	Firmicutes	Bacilli	Lactobacillales	Enterococcaceae	Tetragenococcus	NA
IL.13	ASV_9d0_o06	0,138248975	0,541886983	0,92897956	Bacteria	Firmicutes	Negativicutes	Veillonellales-Selenomonadales	Veillonellaceae	Negativicoccus	NA
IL.13	ASV_9eq_kr6	-0,0588223	0,53726359	0,935844759	Bacteria	Firmicutes	Bacilli	Lactobacillales	Streptococcaceae	Streptococcus	mitis/parasanguinis
IL.13	ASV_9jb_2ek	-0,136599978	0,559746752	0,939075451	Bacteria	Actinobacteriota	Actinobacteria	Corynebacteriales	Corynebacteriaceae	Corynebacterium	NA
IL.13	ASV_9kg_f3f	-0,083363251	0,519565098	0,91467342	Bacteria	Actinobacteriota	Actinobacteria	Corynebacteriales	NA	NA	NA
IL.13	ASV_9l0_247	0,039454672	0,576938979	0,935892756	Bacteria	Firmicutes	Bacilli	Staphylococcales	Gemellaceae	Gemella	morbillorum
IL.13	ASV_9mx_kho	-0,083460251	0,50199176	0,928926436	Bacteria	Actinobacteriota	Actinobacteria	Frankiales	Sporichthyaceae	NA	NA

Variable	FeatureID	spearman.erho	spearman.ep	spearman.eBH	Domain	Phylum	Class	Order	Family	Genus	Species
IL.13	ASV_9o6_1ja	-0,14064972	0,589301571	0,947536934	Bacteria	Firmicutes	Bacilli	Staphylococcales	Staphylococcaceae	Staphylococcus	aureus
IL.13	ASV_9vy_jj2	-0,060851212	0,56472086	0,937022305	Bacteria	Firmicutes	Bacilli	Staphylococcales	Staphylococcaceae	Staphylococcus	NA
IL.13	ASV_9wz_9u 7	-0,013216224	0,52045415	0,929272076	Bacteria	Actinobacteriota	Actinobacteria	NA	NA	NA	NA
IL.13	ASV_aew_fyi	0,071060442	0,521765798	0,927870932	Bacteria	Actinobacteriota	Actinobacteria	Corynebacteriales	Corynebacteriaceae	Corynebacterium	mucifaciens
IL.13	ASV_ajo_fen	0,42591807	0,119182795	0,739670338	Bacteria	Actinobacteriota	Actinobacteria	Corynebacteriales	Corynebacteriaceae	Corynebacterium	NA
IL.13	ASV_aqj_vcn	0,014501471	0,651254814	0,957905552	Bacteria	Firmicutes	Bacilli	Lactobacillales	Streptococcaceae	Streptococcus	mitis/phage/pneumoniae/pseudopneumoniae
IL.13	ASV_arl_xwg	0,00665257	0,570362025	0,941530687	Bacteria	Firmicutes	Clostridia	Lachnospirales	Lachnospiraceae	Blautia	NA
IL.13	ASV_aw8_q9 6	0,213795658	0,424870092	0,911980338	Bacteria	Actinobacteriota	Actinobacteria	Corynebacteriales	Corynebacteriaceae	Corynebacterium	NA
IL.13	ASV_bg2_zbh	-0,194072364	0,438456389	0,892982491	Bacteria	Firmicutes	Bacilli	Staphylococcales	Staphylococcaceae	Staphylococcus	NA
IL.13	ASV_bh3_94k	0,016101968	0,551500747	0,932278227	Bacteria	Firmicutes	Bacilli	Lactobacillales	Streptococcaceae	Lactococcus	NA
IL.13	ASV_bnk_uhh	0,018284464	0,723836166	0,966170084	Bacteria	Firmicutes	Clostridia	Peptostreptococcales-Tissierellales	Family XI	Anaerococcus	provencensis
IL.13	ASV_bsd_ayc	-0,144529713	0,49039251	0,905320917	Bacteria	Planctomycetota	Planctomycetes	Pirellulales	Pirellulaceae	NA	NA
IL.13	ASV_btl_vxl	0,093661397	0,585001714	0,947630849	Bacteria	Firmicutes	Clostridia	Peptostreptococcales-Tissierellales	Family XI	Anaerococcus	vaginalis
IL.13	ASV_bwx_we f	0,068004948	0,545623262	0,934616788	Bacteria	Actinobacteriota	Actinobacteria	Corynebacteriales	Corynebacteriaceae	Corynebacterium	amycolatum/jeikeium/lactis/urealyticum/vitaeruminis
IL.13	ASV_c46_jpb	-0,153631528	0,504140248	0,927095582	Bacteria	Actinobacteriota	Actinobacteria	Corynebacteriales	Corynebacteriaceae	Corynebacterium	NA
IL.13	ASV_c4o_xm s	0,219421647	0,421902788	0,888643575	Bacteria	Firmicutes	Bacilli	Staphylococcales	Staphylococcaceae	NA	NA
IL.13	ASV_cep_rux	-0,011971393	0,623067425	0,951485212	Bacteria	Firmicutes	Clostridia	Peptostreptococcales-Tissierellales	Family XI	Anaerococcus	NA
IL.13	ASV_cje_lx4	-0,058062468	0,497600189	0,916698883	Bacteria	Proteobacteria	Gammaproteobacteria	Burkholderiales	Neisseriaceae	NA	NA
IL.13	ASV_co0_sbi	0,001503497	0,567804867	0,931304044	Bacteria	Actinobacteriota	Actinobacteria	Corynebacteriales	Corynebacteriaceae	Corynebacterium	NA
IL.13	ASV_cqp_otd	0,015503803	0,551241815	0,928421763	Bacteria	Firmicutes	Negativicutes	Veillonellales-Selenomonadales	Veillonellaceae	Negativicoccus	NA
IL.13	ASV_cy2_em p	-0,10404846	0,501963118	0,907031397	Bacteria	Actinobacteriota	Actinobacteria	Corynebacteriales	Corynebacteriaceae	Corynebacterium	NA
IL.13	ASV_d0q_oe1	0,013094974	0,625612376	0,946580901	Bacteria	Bacteroidota	Bacteroidia	Bacteroidales	Prevotellaceae	Prevotella_9	NA
IL.13	ASV_da2_u30	-0,005456239	0,506131586	0,91623274	Bacteria	Bacteroidota	Bacteroidia	Bacteroidales	Prevotellaceae	Prevotella	buccalis
IL.13	ASV_dhe_20n	-0,152257364	0,557689839	0,947427603	Bacteria	Firmicutes	Bacilli	Staphylococcales	Staphylococcaceae	Staphylococcus	aureus
IL.13	ASV_dlu_bk7	0,337737162	0,219711136	0,841647758	Bacteria	Proteobacteria	Gammaproteobacteria	Burkholderiales	Neisseriaceae	NA	NA
IL.13	ASV_dmt_o1x	-0,054764474	0,601656275	0,938373637	Bacteria	Proteobacteria	Gammaproteobacteria	Enterobacterales	Pasteurellaceae	Haemophilus	haemolyticus/influenzae
IL.13	ASV_dui_4x1	0,13063449	0,5328715	0,924444632	Bacteria	Firmicutes	Bacilli	Lactobacillales	Carnobacteriaceae	Dolosigranulum	NA
IL.13	ASV_dz0_fy2	0,262449145	0,359714068	0,872348981	Bacteria	Actinobacteriota	Actinobacteria	Corynebacteriales	Corynebacteriaceae	Corynebacterium	NA
IL.13	ASV_e7g_uqv	-0,054538142	0,475116236	0,917252831	Bacteria	Actinobacteriota	Thermoleophilia	Gaiellales	NA	NA	NA
IL.13	ASV_e8x_8cs	-0,070454193	0,692502531	0,964263314	Bacteria	Actinobacteriota	Actinobacteria	Corynebacteriales	Corynebacteriaceae	Lawsonella	NA
IL.13	ASV_elz_ffm	0,072442689	0,562815105	0,934483269	Bacteria	Actinobacteriota	Actinobacteria	Corynebacteriales	Corynebacteriaceae	Corynebacterium	NA
IL.13	ASV_f1j_s2e	-0,173831738	0,47412315	0,908183567	Bacteria	Actinobacteriota	Actinobacteria	Corynebacteriales	Corynebacteriaceae	Corynebacterium	aurimucosum/simulans/striatum/xerosis

Variable	FeatureID	spearman.erho	spearman.ep	spearman.eBH	Domain	Phylum	Class	Order	Family	Genus	Species
IL.13	ASV_f6c_v0m	-0,109367283	0,557857117	0,914744664	Bacteria	Actinobacteriota	Actinobacteria	Corynebacteriales	Corynebacteriaceae	Corynebacterium	NA
IL.13	ASV_f8c_ir4	-0,008802733	0,580338845	0,945187493	Bacteria	Bacteroidota	Bacteroidia	Bacteroidales	Bacteroidaceae	Bacteroides	plebeius
IL.13	ASV_fb6_dje	-0,202220348	0,411424255	0,889256395	Bacteria	Firmicutes	Bacilli	Staphylococcales	Staphylococcaceae	Staphylococcus	aureus/devriesei/epidermidis/haemolyticus/hominis
IL.13	ASV_fgq_ohv	0,105568123	0,554107505	0,926888322	Bacteria	Actinobacteriota	Actinobacteria	Corynebacteriales	Corynebacteriaceae	Corynebacterium	NA
IL.13	ASV_fmb_men	0,006959736	0,568419877	0,939314439	Bacteria	Actinobacteriota	Actinobacteria	Corynebacteriales	Corynebacteriaceae	Corynebacterium	NA
IL.13	ASV_fqm_8pm	0,180031642	0,497129445	0,929610706	Bacteria	Firmicutes	Bacilli	Lactobacillales	Streptococcaceae	Streptococcus	anginosus/cristatus/infantis/mitis/oralis/pneumoniae/pseudopneumoniae/sanguinis/timonensis
IL.13	ASV_fug_ldj	-0,180225642	0,418263036	0,892903293	Bacteria	Bacteroidota	Bacteroidia	Bacteroidales	Prevotellaceae	Prevotella_9	NA
IL.13	ASV_g3d_cs4	-0,088172825	0,488837392	0,917074567	Bacteria	Proteobacteria	Gammaproteobacteria	Enterobacterales	Pasteurellaceae	Haemophilus	influenzae
IL.13	ASV_geq_907	-0,071262525	0,745971606	0,970791504	Bacteria	Firmicutes	Clostridia	Peptostreptococcales-Tissierellales	Family XI	Anaerococcus	octavius
IL.13	ASV_gg8_mtq	-0,035655512	0,561237451	0,935067868	Bacteria	Bacteroidota	Bacteroidia	Bacteroidales	Muribaculaceae	NA	NA
IL.13	ASV_gzb_mis	0,013014141	0,574546704	0,937122978	Bacteria	Firmicutes	Clostridia	Lachnospirales	Lachnospiraceae	Coprococcus	catus
IL.13	ASV_h1r_zyt	-0,058878883	0,560211388	0,927415385	Bacteria	Fusobacteriota	Fusobacteriia	Fusobacteriales	Fusobacteriaceae	Fusobacterium	NA
IL.13	ASV_h40_0md	0,177816813	0,48292989	0,901203217	Bacteria	Actinobacteriota	Actinobacteria	Corynebacteriales	Corynebacteriaceae	Corynebacterium	NA
IL.13	ASV_h4w_6wi	-0,096749224	0,492046908	0,920707715	Bacteria	Firmicutes	Bacilli	Staphylococcales	Staphylococcaceae	NA	NA
IL.13	ASV_hbi_ebs	0,081261588	0,56702142	0,93828145	Bacteria	Actinobacteriota	Actinobacteria	Corynebacteriales	Corynebacteriaceae	Lawsonella	clevelandensis
IL.13	ASV_het_bu3	-0,067754365	0,531439446	0,926884507	Bacteria	Campylobacterota	Campylobacteria	Campylobacterales	Campylobacteraceae	Campylobacter	NA
IL.13	ASV_hjn_92c	0,079475175	0,704264062	0,965729193	Bacteria	Firmicutes	Clostridia	Peptostreptococcales-Tissierellales	Family XI	Finegoldia	magna
IL.13	ASV_hkw_6s3	-0,053697477	0,551915731	0,905317536	Bacteria	Actinobacteriota	Actinobacteria	Corynebacteriales	Corynebacteriaceae	Corynebacterium	NA
IL.13	ASV_hpm_svr	0,366360188	0,193386892	0,799691516	Bacteria	Actinobacteriota	Actinobacteria	Corynebacteriales	Corynebacteriaceae	Corynebacterium	NA
IL.13	ASV_hq8_93x	0,319008116	0,259882379	0,841165581	Bacteria	Actinobacteriota	Actinobacteria	Corynebacteriales	Corynebacteriaceae	Corynebacterium	NA
IL.13	ASV_hqq_2ph	-0,177113565	0,453828747	0,89164012	Bacteria	Proteobacteria	Gammaproteobacteria	Burkholderiales	Oxalobacteraceae	Massilia	timonae
IL.13	ASV_i0i_mxo	-0,132978652	0,490812134	0,912319298	Bacteria	Actinobacteriota	Actinobacteria	Corynebacteriales	Corynebacteriaceae	Corynebacterium	pyruviciproducens
IL.13	ASV_i35_0op	0,483261123	0,065717925	0,686455712	Bacteria	Actinobacteriota	Actinobacteria	Corynebacteriales	Corynebacteriaceae	Corynebacterium	aurimucosum/jeikeium/propinquum/pseudodiphtheriticum
IL.13	ASV_i88_mm0	-0,197184441	0,404500967	0,874648231	Bacteria	Actinobacteriota	Actinobacteria	Corynebacteriales	Corynebacteriaceae	Corynebacterium	NA
IL.13	ASV_ic5_nai	-0,280523442	0,355165846	0,841891686	Bacteria	Firmicutes	Bacilli	Lactobacillales	Streptococcaceae	Streptococcus	sobrinus
IL.13	ASV_igm_m49	-0,264122392	0,352347649	0,844310246	Bacteria	NA	NA	NA	NA	NA	NA
IL.13	ASV_ii5_tjz	0,017209382	0,627565523	0,944263781	Bacteria	Firmicutes	Bacilli	Staphylococcales	Staphylococcaceae	Staphylococcus	NA
IL.13	ASV_ikg_ra0	0,360912033	0,197683573	0,816321274	Bacteria	Actinobacteriota	Actinobacteria	Corynebacteriales	Corynebacteriaceae	Corynebacterium	NA
IL.13	ASV_iov_l70	0,158812934	0,494646599	0,913974954	Bacteria	Firmicutes	Bacilli	Lactobacillales	Carnobacteriaceae	Dolosigranulum	pigrum
IL.13	ASV_iqo_ea7	-0,142896883	0,48466956	0,904316319	Bacteria	Firmicutes	Bacilli	Staphylococcales	Staphylococcaceae	Staphylococcus	NA
IL.13	ASV_jjw_h6c	0,189820539	0,454786826	0,937118702	Bacteria	Actinobacteriota	Actinobacteria	Corynebacteriales	Corynebacteriaceae	Corynebacterium	NA

Variable	FeatureID	spearman.erho	spearman.ep	spearman.eBH	Domain	Phylum	Class	Order	Family	Genus	Species
IL.13	ASV_jl5_6pn	-0,220060229	0,412801907	0,913302236	Bacteria	Firmicutes	Bacilli	Staphylococcales	Staphylococcaceae	Staphylococcus	aureus
IL.13	ASV_jp2_wik	0,43254639	0,087468795	0,773785067	Bacteria	Firmicutes	Bacilli	Staphylococcales	Staphylococcaceae	Staphylococcus	epidermidis
IL.13	ASV_jph_cup	0,40749619	0,13376544	0,777100114	Bacteria	Actinobacteriota	Actinobacteria	Corynebacteriales	Corynebacteriaceae	Corynebacterium	NA
IL.13	ASV_jy3_f61	-0,140245555	0,486713767	0,89811505	Bacteria	Actinobacteriota	Actinobacteria	Corynebacteriales	Corynebacteriaceae	Corynebacterium	NA
IL.13	ASV_jy9_jqd	0,021234874	0,549975972	0,933871203	Bacteria	Firmicutes	Clostridia	Lachnospirales	Lachnospiraceae	[Eubacterium] ventriosum group	NA
IL.13	ASV_k2s_cq m	0,15424586	0,511792905	0,928610823	Bacteria	Actinobacteriota	Actinobacteria	Corynebacteriales	Corynebacteriaceae	Corynebacterium	NA
IL.13	ASV_k3h_3z5	0,008002484	0,527758292	0,931556679	Bacteria	Proteobacteria	Gammaproteobacteria	Xanthomonadales	Xanthomonadaceae	Lysobacter	NA
IL.13	ASV_k3h_qcz	-0,033764016	0,49845353	0,920557666	Bacteria	Firmicutes	Bacilli	Staphylococcales	Staphylococcaceae	Staphylococcus	NA
IL.13	ASV_kaj_b2n	-0,100677717	0,506430694	0,935103099	Bacteria	Firmicutes	Bacilli	Lactobacillales	P5D1-392	NA	NA
IL.13	ASV_kcs_cbi	0,157907603	0,500740946	0,902109875	Bacteria	Actinobacteriota	Actinobacteria	Corynebacteriales	Corynebacteriaceae	Corynebacterium	NA
IL.13	ASV_ki3_dfk	0,244140431	0,385720447	0,892253092	Bacteria	Actinobacteriota	Actinobacteria	Corynebacteriales	Corynebacteriaceae	Corynebacterium	NA
IL.13	ASV_kkl_mxy	-0,315257457	0,266990632	0,851457691	Bacteria	Actinobacteriota	Actinobacteria	Corynebacteriales	Corynebacteriaceae	Corynebacterium	kroppenstedtii
IL.13	ASV_kru_fff	-0,179069727	0,404671577	0,875019259	Bacteria	Proteobacteria	Gammaproteobacteria	Enterobacterales	Erwiniaceae	NA	NA
IL.13	ASV_ktj_lyl	-0,13409415	0,485970842	0,911364287	Bacteria	Proteobacteria	Gammaproteobacteria	Burkholderiales	Neisseriaceae	NA	NA
IL.13	ASV_l3n_w4d	-0,086758244	0,510270695	0,927087286	Bacteria	Synergistota	Synergistia	Synergistales	Synergistaceae	Jonquetella	anthropi
IL.13	ASV_lky_mqj	-0,09727464	0,514992688	0,917211352	Bacteria	Firmicutes	Bacilli	Lactobacillales	Streptococcaceae	Streptococcus	NA
IL.13	ASV_lmq_jff	0,132647236	0,517130292	0,923856018	Bacteria	Firmicutes	Bacilli	Lactobacillales	Carnobacteriaceae	Dolosigranulum	NA
IL.13	ASV_lnh_9fs	-0,239395524	0,369374456	0,879638397	Bacteria	Actinobacteriota	Actinobacteria	Corynebacteriales	Corynebacteriaceae	Corynebacterium	afermentans/coyleae
IL.13	ASV_lvd_sr9	-0,144650962	0,550201624	0,941175143	Bacteria	Actinobacteriota	Actinobacteria	Corynebacteriales	Corynebacteriaceae	Corynebacterium	NA
IL.13	ASV_lvh_wn0	-0,242701601	0,38852591	0,884544223	Bacteria	Actinobacteriota	Actinobacteria	Micrococcales	Dermabacteraceae	Dermabacter	jinjuensis
IL.13	ASV_m52_gp v	-0,105180124	0,495881307	0,910024774	Bacteria	Actinobacteriota	Actinobacteria	Corynebacteriales	Corynebacteriaceae	Corynebacterium	NA
IL.13	ASV_mbn_3b 6	0,033570017	0,566189972	0,924130708	Bacteria	Firmicutes	Clostridia	Lachnospirales	Lachnospiraceae	[Ruminococcus] torques group	NA
IL.13	ASV_mcc_yp e	-0,062960958	0,567318217	0,935732862	Bacteria	Firmicutes	Bacilli	Staphylococcales	Staphylococcaceae	Staphylococcus	epidermidis
IL.13	ASV_mdt_jke	-0,184816966	0,424441972	0,894755752	Bacteria	Proteobacteria	Gammaproteobacteria	Enterobacterales	Pasteurellaceae	Haemophilus	haemolyticus/influenzae
IL.13	ASV_mgr_u9 o	0,267396135	0,360100406	0,870352193	Bacteria	Actinobacteriota	Actinobacteria	Corynebacteriales	Corynebacteriaceae	Corynebacterium	NA
IL.13	ASV_mlb_2sd	-0,188931374	0,452698573	0,910630383	Bacteria	Firmicutes	Bacilli	Staphylococcales	Staphylococcaceae	Staphylococcus	NA
IL.13	ASV_mm9_js 5	-0,150002118	0,549839298	0,933750043	Bacteria	Firmicutes	Bacilli	Staphylococcales	Staphylococcaceae	Staphylococcus	aureus
IL.13	ASV_mvn_oe m	0,04861307	0,588843261	0,935637492	Bacteria	Actinobacteriota	Actinobacteria	Corynebacteriales	Corynebacteriaceae	Corynebacterium	NA
IL.13	ASV_n1a_pw 6	-0,101631548	0,516323135	0,923329207	Bacteria	Proteobacteria	Alphaproteobacteria	Rhizobiales	Methyloligellaceae	NA	NA
IL.13	ASV_n5j_9sq	-0,011971393	0,548832259	0,934839884	Bacteria	Firmicutes	Bacilli	Staphylococcales	Staphylococcaceae	Staphylococcus	NA
IL.13	ASV_n79_65k	-0,143082799	0,46936155	0,89250663	Bacteria	Proteobacteria	Gammaproteobacteria	Burkholderiales	Neisseriaceae	NA	NA
IL.13	ASV_nbq_6pg	-0,106885704	0,452102715	0,902043314	Bacteria	Firmicutes	Clostridia	Peptostreptococcales-Tissierellales	Family XI	Anaerococcus	prevotii/tetradius

Variable	FeatureID	spearman.erho	spearman.ep	spearman.eBH	Domain	Phylum	Class	Order	Family	Genus	Species
IL.13	ASV_nk5_hwz	-0,049599235	0,535115252	0,930044118	Bacteria	Bacteroidota	Bacteroidia	Flavobacteriales	Weeksellaceae	Cloacibacterium	normanense
IL.13	ASV_ntr_hh5	-0,002028913	0,60057213	0,942558758	Bacteria	Proteobacteria	Gammaproteobacteria	Enterobacterales	Pasteurellaceae	Haemophilus	haemolyticus/influenzae/parainfluenzae
IL.13	ASV_nvc_oao	-0,135274314	0,579091531	0,940674241	Bacteria	Deinococcota	Deinococci	Thermales	Thermaceae	Thermus	parvatiensis/thermophilus
IL.13	ASV_o0r_len	-0,081698088	0,66776872	0,96245063	Bacteria	Firmicutes	Bacilli	Staphylococcales	Staphylococcaceae	Staphylococcus	aureus/capitis/caprae/epidermidis/haemolyticus/saprophyticus/warneri
IL.13	ASV_o10_2p1	-0,262982644	0,368152964	0,841629351	Bacteria	Proteobacteria	Gammaproteobacteria	Pseudomonadales	Pseudomonadaceae	Pseudomonas	brassicacearum/chlororaphis/corrugata/fluorescens/jessenii/kilonensis/mohnii/putida/synxantha
IL.13	ASV_o1f_10k	-0,207741254	0,391435786	0,867118057	Bacteria	Proteobacteria	Gammaproteobacteria	Burkholderiales	Comamonadaceae	NA	NA
IL.13	ASV_ode_i48	-0,013709306	0,542497605	0,937610412	Bacteria	Actinobacteriota	Actinobacteria	Corynebacteriales	Corynebacteriaceae	Corynebacterium	NA
IL.13	ASV_on0_uxs	0,003281827	0,666020497	0,963209077	Bacteria	Actinobacteriota	Actinobacteria	Corynebacteriales	Corynebacteriaceae	Corynebacterium	afermentans/ihumii
IL.13	ASV_onw_luq	-0,059832714	0,559839778	0,935204967	Bacteria	Proteobacteria	Gammaproteobacteria	Burkholderiales	Neisseriaceae	Eikenella	NA
IL.13	ASV_ouf_s4v	-0,202891263	0,44835212	0,88767065	Bacteria	Actinobacteriota	Actinobacteria	Corynebacteriales	Corynebacteriaceae	Corynebacterium	NA
IL.13	ASV_oyd_63r	-0,194880696	0,405530375	0,902340294	Bacteria	Firmicutes	Bacilli	Staphylococcales	Staphylococcaceae	Staphylococcus	NA
IL.13	ASV_p9u_72k	-0,01513197	0,540015249	0,927040702	Bacteria	Firmicutes	Clostridia	Peptococcales	Peptococcaceae	Peptococcus	NA
IL.13	ASV_paz_uyl	-0,092214483	0,676837275	0,962897611	Bacteria	Actinobacteriota	Actinobacteria	Propionibacteriales	Propionibacteriaceae	Cutibacterium	acnes/avidum
IL.13	ASV_pix_o0d	-0,183386219	0,43593067	0,88504676	Bacteria	Desulfobacterota	Desulfovibrionia	Desulfovibrionales	Desulfohalobiaceae	Desulfovermiculus	NA
IL.13	ASV_pjp_yzq	-0,193304449	0,414463193	0,890059921	Bacteria	Firmicutes	Bacilli	Staphylococcales	Staphylococcaceae	Staphylococcus	NA
IL.13	ASV_pni_ryk	-0,133043319	0,476116815	0,901450627	Bacteria	Actinobacteriota	Actinobacteria	Corynebacteriales	Corynebacteriaceae	Corynebacterium	NA
IL.13	ASV_pwy_pjt	0,243518016	0,379825455	0,879282611	Bacteria	Actinobacteriota	Actinobacteria	Corynebacteriales	Corynebacteriaceae	Corynebacterium	NA
IL.13	ASV_q1f_0em	0,271987459	0,348072162	0,880962034	Bacteria	Actinobacteriota	Actinobacteria	Corynebacteriales	Corynebacteriaceae	Corynebacterium	NA
IL.13	ASV_q1k_ad2	0,189974122	0,451663716	0,910333016	Bacteria	Firmicutes	Bacilli	Staphylococcales	Staphylococcaceae	Staphylococcus	NA
IL.13	ASV_q1o_24k	0,046842824	0,846500055	0,978835146	Bacteria	Actinobacteriota	Actinobacteria	Corynebacteriales	Corynebacteriaceae	Corynebacterium	accolens/fastidiosum
IL.13	ASV_q1r_s4c	-0,103005712	0,476810718	0,913527776	Bacteria	Actinobacteriota	Actinobacteria	Actinomycetales	Actinomycetaceae	Actinotignum	schaalii/timonense
IL.13	ASV_qc8_76v	0,012634225	0,617821596	0,943193552	Bacteria	Proteobacteria	Gammaproteobacteria	Enterobacterales	Pasteurellaceae	Haemophilus	influenzae
IL.13	ASV_qft_24l	-0,065038371	0,498618068	0,929940413	Bacteria	Bacteroidota	Bacteroidia	Bacteroidales	Prevotellaceae	Alloprevotella	NA
IL.13	ASV_qis_xxl	-0,117701183	0,478032817	0,913731585	Bacteria	Firmicutes	Clostridia	Peptostreptococcales-Tissierellales	Family XI	Anaerococcus	nagyae
IL.13	ASV_qk1_qy8	-0,118097265	0,449199812	0,896750589	Bacteria	Proteobacteria	Gammaproteobacteria	Enterobacterales	Pasteurellaceae	Lonepinella	NA
IL.13	ASV_r73_72u	-0,08404225	0,524357491	0,922850043	Bacteria	Actinobacteriota	Actinobacteria	Corynebacteriales	Corynebacteriaceae	Corynebacterium	NA
IL.13	ASV_r8p_k1e	-0,042599082	0,71976495	0,967300917	Bacteria	Firmicutes	Bacilli	Staphylococcales	Staphylococcaceae	Staphylococcus	aureus
IL.13	ASV_r9n_48y	-0,096417808	0,526734212	0,926692143	Bacteria	Firmicutes	Bacilli	Staphylococcales	Staphylococcaceae	Staphylococcus	NA
IL.13	ASV_rad_25g	-0,172231241	0,437557623	0,900927418	Bacteria	Proteobacteria	Gammaproteobacteria	Enterobacterales	Pasteurellaceae	NA	NA
IL.13	ASV_raj_rv1	0,308556387	0,219391018	0,883510063	Bacteria	Firmicutes	Bacilli	Staphylococcales	Staphylococcaceae	Staphylococcus	aureus/capitis/caprae/epidermidis/haemolyticus/warneri
IL.13	ASV_rmx_t9x	-0,170598411	0,50590097	0,933363975	Bacteria	Firmicutes	Bacilli	Staphylococcales	Staphylococcaceae	Staphylococcus	aureus
IL.13	ASV_rs1_k0s	0,011712727	0,485822716	0,917985467	Bacteria	Firmicutes	Bacilli	Staphylococcales	Staphylococcaceae	Staphylococcus	NA
IL.13	ASV_rtx_tpz	-0,080348174	0,517593178	0,918653003	Bacteria	Proteobacteria	Gammaproteobacteria	Pseudomonadales	Moraxellaceae	Moraxella	catarrhalis/nonliquefaciens

Variable	FeatureID	spearman.erho	spearman.ep	spearman.eBH	Domain	Phylum	Class	Order	Family	Genus	Species
IL.13	ASV_s8r_baf	-0,212890327	0,422787148	0,919330549	Bacteria	Firmicutes	Clostridia	Peptostreptococcales-Tissierellales	Family XI	Anaerococcus	NA
IL.13	ASV_sjv_qdw	-0,138111559	0,490067556	0,923012911	Bacteria	Actinobacteriota	Actinobacteria	Corynebacteriales	Corynebacteriaceae	Corynebacterium	NA
IL.13	ASV_snw_j03	-0,025357366	0,56264159	0,934364443	Bacteria	Firmicutes	Bacilli	Lactobacillales	Streptococcaceae	Streptococcus	NA
IL.13	ASV_ssr_vha	-0,174340987	0,50460633	0,932999663	Bacteria	Firmicutes	Bacilli	Staphylococcales	Staphylococcaceae	Staphylococcus	aureus
IL.13	ASV_t7p_n06	0,183871218	0,460337536	0,916578088	Bacteria	Firmicutes	Clostridia	Peptostreptococcales-Tissierellales	Family XI	Anaerococcus	NA
IL.13	ASV_t8l_ysz	0,244342514	0,379632063	0,898156217	Bacteria	Firmicutes	Bacilli	Staphylococcales	Staphylococcaceae	NA	NA
IL.13	ASV_ten_mjo	0,066461035	0,546774214	0,938029405	Bacteria	Actinobacteriota	Actinobacteria	Corynebacteriales	Corynebacteriaceae	Corynebacterium	NA
IL.13	ASV_tu2_xgy	0,345545646	0,235460651	0,811062522	Bacteria	Actinobacteriota	Actinobacteria	Corynebacteriales	Corynebacteriaceae	Corynebacterium	NA
IL.13	ASV_tvh_ar7	-0,040554003	0,546778306	0,929532384	Bacteria	Firmicutes	Bacilli	Lactobacillales	Streptococcaceae	Streptococcus	anginosus/constellatus/intermedius/lutetiensis
IL.13	ASV_v7r_7sn	-0,16089843	0,45721898	0,894203196	Bacteria	Proteobacteria	Gammaproteobacteria	Burkholderiales	Comamonadaceae	Rubrivivax	NA
IL.13	ASV_xex_kso	-0,302671732	0,300308533	0,839914904	Bacteria	Actinobacteriota	Actinobacteria	Micrococcales	Bogoriellaceae	Georgenia	NA

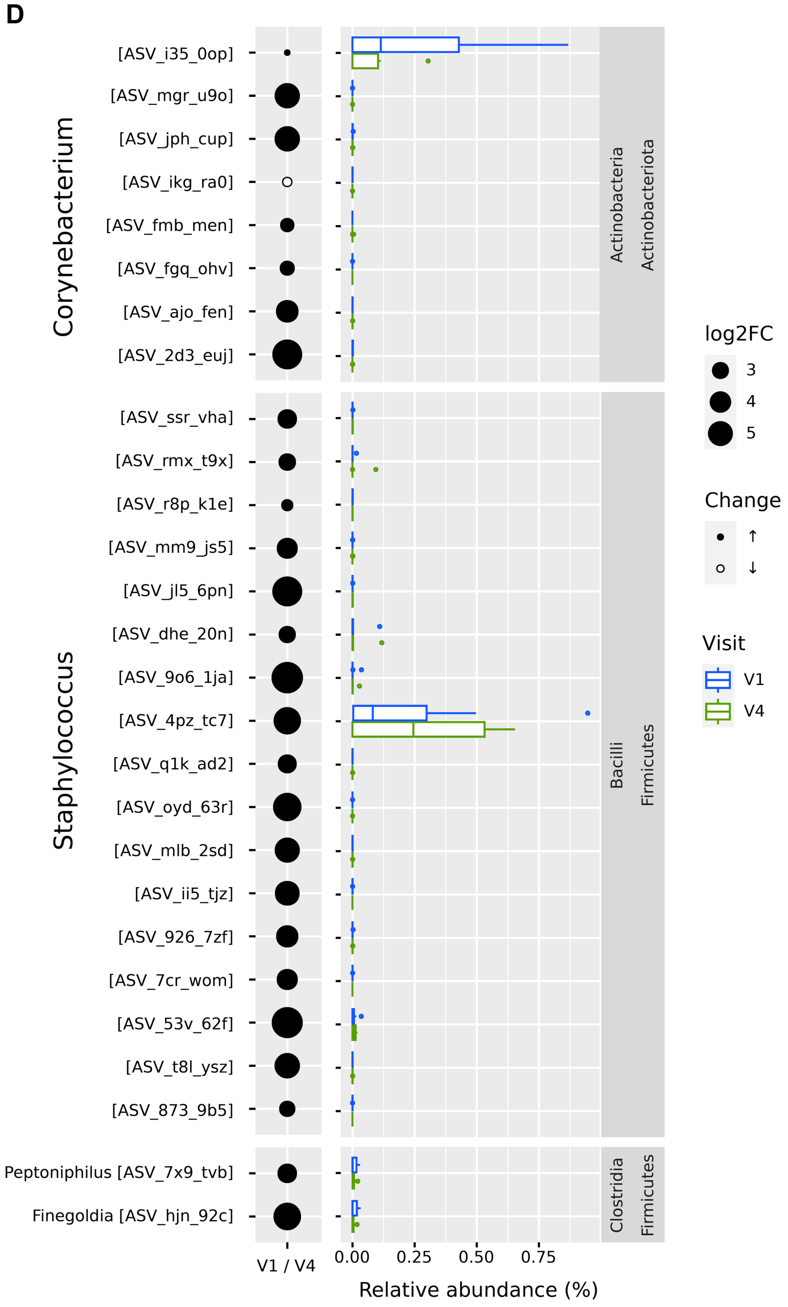

Supplementary Figure 1. Pairwise differential abundance testing of ASVs as analysed by DESeq2 with α=0.05 between V1 (before treatment, blue) and V4 (after 24 weeks of dupilumab, green). The circles represent significant log2 fold changes and the color of the circles represents an increase or decrease in relative abundance in V4 compared to V1. Bar charts represent the average relative abundance of significantly different ASVs on both timepoints.

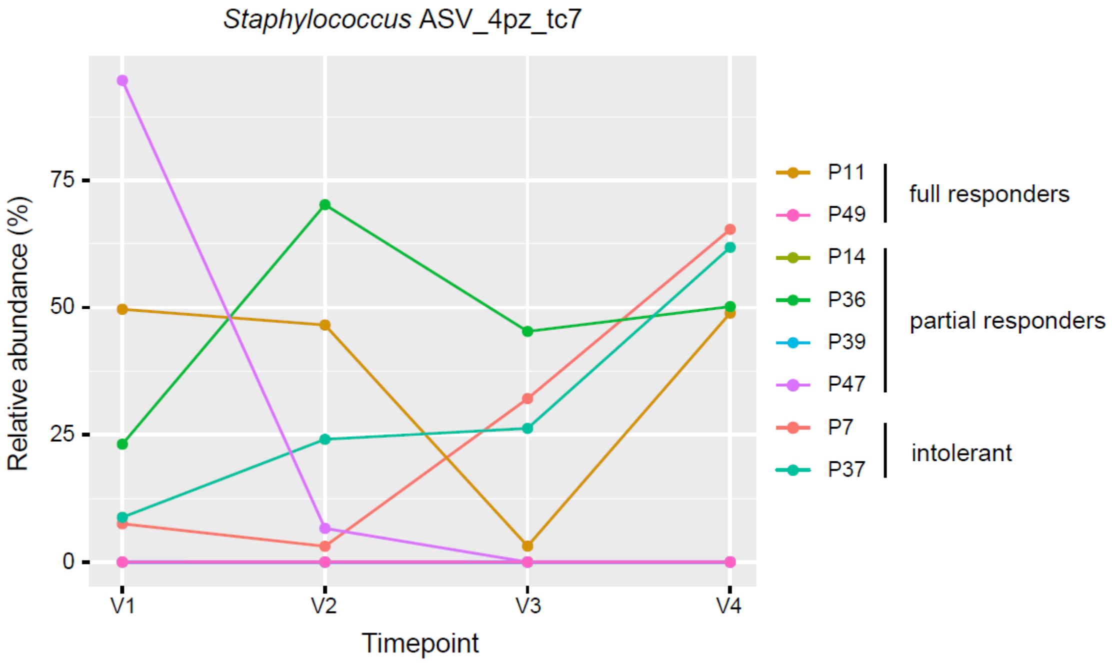

Supplementary Figure 2. Relative abundance (%) of *Staphylococcus* ASV_4pz_tc7 in individual patients at V1 (before treatment), V2 (after 4 weeks of treatment), V3 (after 12 weeks of treatment) and at V4 (after 24 weeks of treatment). Patients who remained aspirin intolerant (n=2), and developed full (n=2) or partial (n=4) aspirin tolerance are indicated.

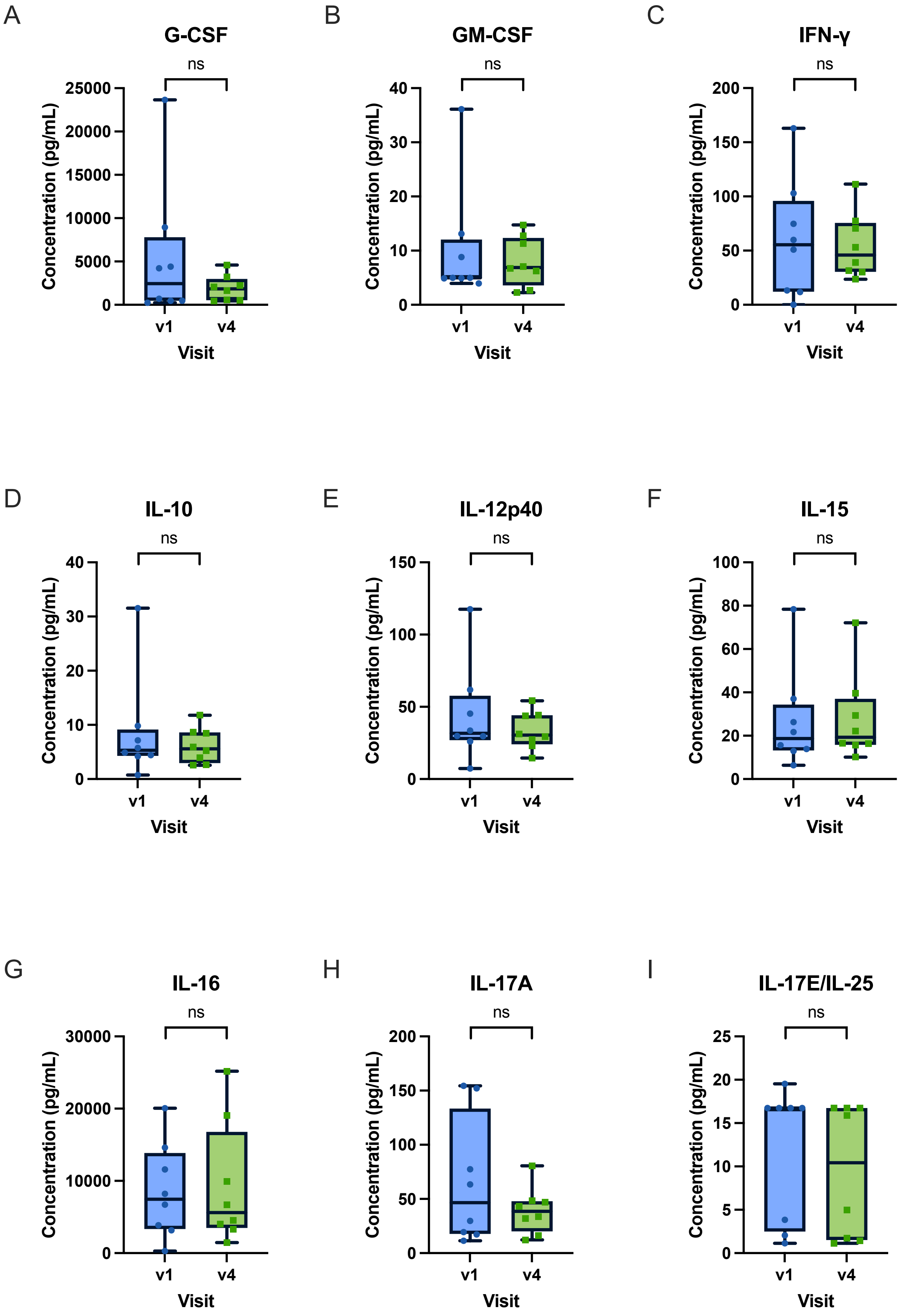
A
G-CSF
ns
Concentration (pg/mL)
0
5000
10000
15000
20000
25000
v1
v4
Visit
B
GM-CSF
ns
Concentration (pg/mL)
0
10
20
30
40
v1
v4
Visit
C
IFN-γ
ns
Concentration (pg/mL)
0
50
100
150
200
v1
v4
Visit
D
IL-10
ns
Concentration (pg/mL)
0
10
20
30
40
v1
v4
Visit
E
IL-12p40
ns
Concentration (pg/mL)
0
50
100
150
v1
v4
Visit
F
IL-15
ns
Concentration (pg/mL)
0
20
40
60
80
100
v1
v4
Visit
G
IL-16
ns
Concentration (pg/mL)
0
10000
20000
30000
v1
v4
Visit
H
IL-17A
ns
Concentration (pg/mL)
0
50
100
150
200
v1
v4
Visit
I
IL-17E/IL-25
ns
Concentration (pg/mL)
0
5
10
15
20
25
v1
v4
Visit

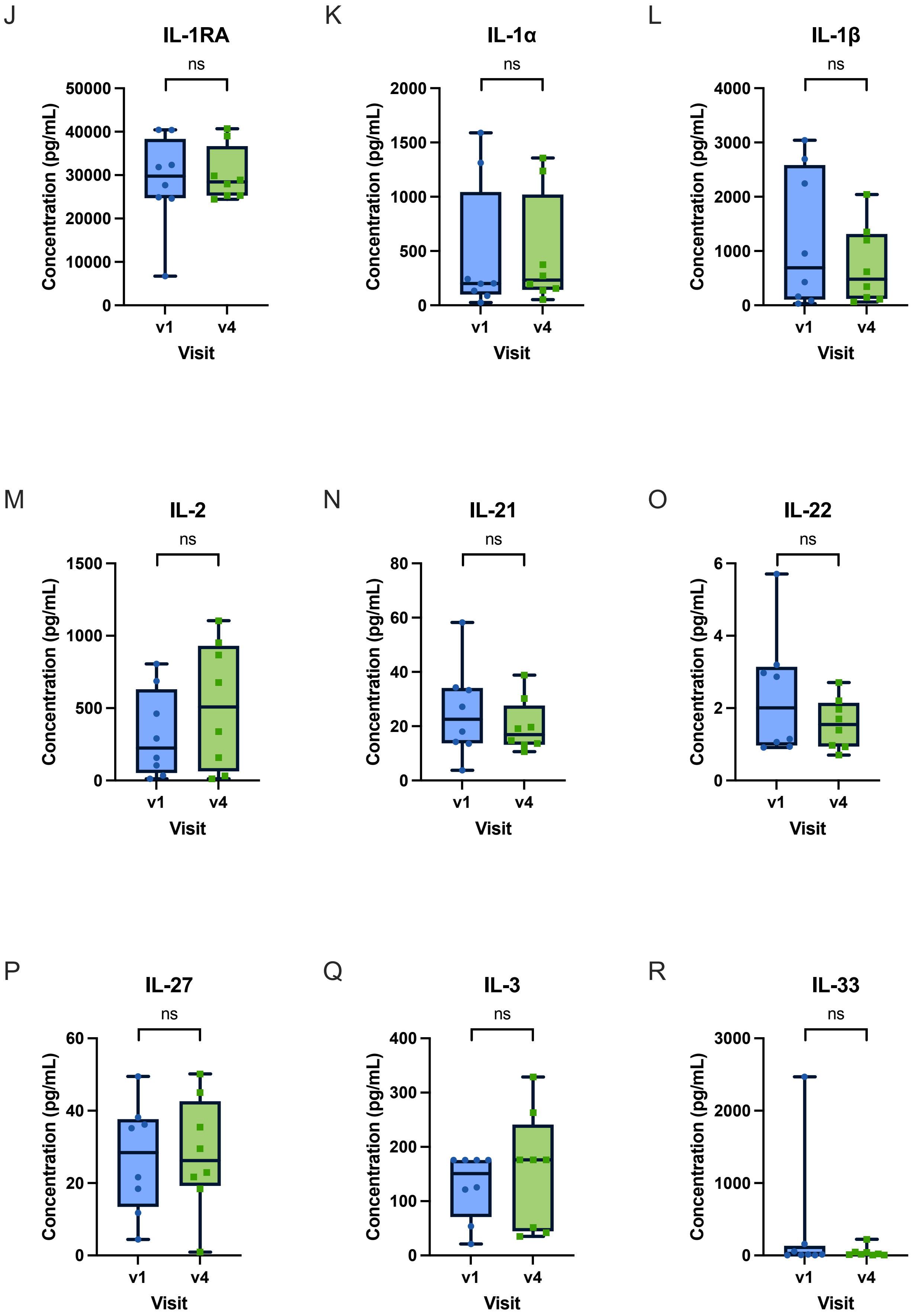
J
IL-1RA
ns
Concentration (pg/mL)
50000
40000
30000
20000
10000
0
v1
v4
Visit
K
IL-1α
ns
Concentration (pg/mL)
2000
1500
1000
500
0
v1
v4
Visit
L
IL-1β
ns
Concentration (pg/mL)
4000
3000
2000
1000
0
v1
v4
Visit
M
IL-2
ns
Concentration (pg/mL)
1500
1000
500
0
v1
v4
Visit
N
IL-21
ns
Concentration (pg/mL)
80
60
40
20
0
v1
v4
Visit
O
IL-22
ns
Concentration (pg/mL)
6
4
2
0
v1
v4
Visit
P
IL-27
ns
Concentration (pg/mL)
60
40
20
0
v1
v4
Visit
Q
IL-3
ns
Concentration (pg/mL)
400
300
200
100
0
v1
v4
Visit
R
IL-33
ns
Concentration (pg/mL)
3000
2000
1000
0
v1
v4
Visit

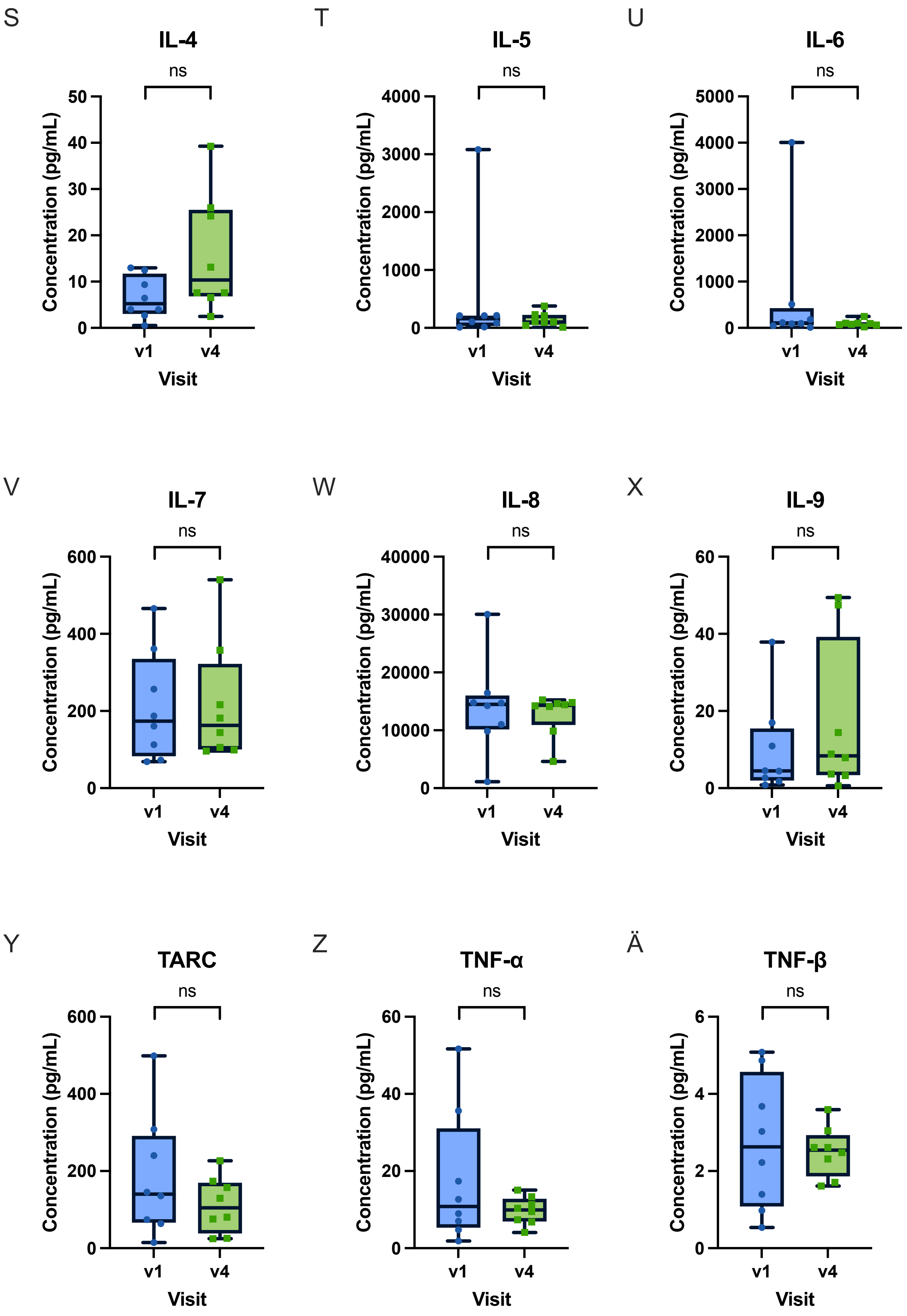
S
IL-4
ns
Concentration (pg/mL)
Visit
v1
v4
T
IL-5
ns
U
IL-6
ns
V
IL-7
ns
W
IL-8
ns
X
IL-9
ns
Y
TARC
ns
Z
TNF-α
ns
Ä
TNF-β
ns

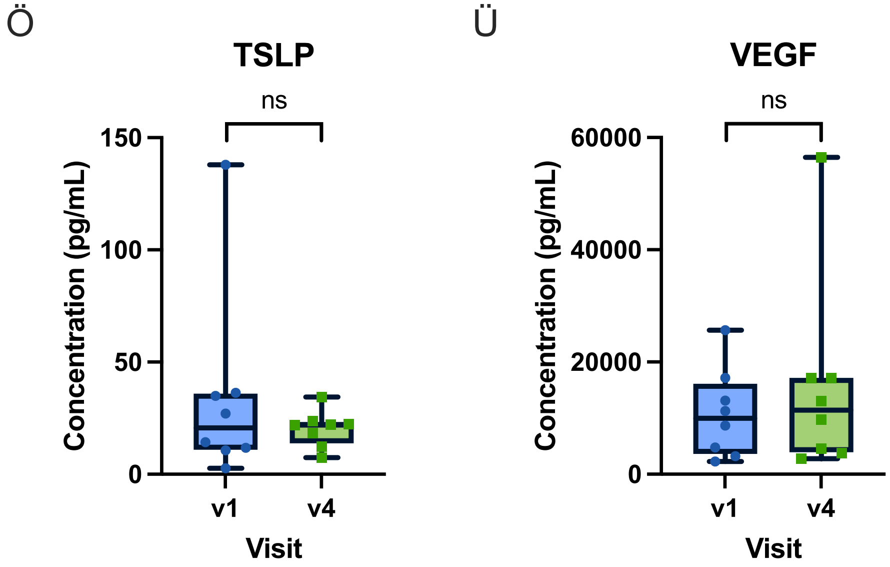

Supplementary Figure 3. Changes in selected biomarkers in nasal secretions of N-ERD patients during 24 weeks of dupilumab treatment. (A-Ü) Levels of (A) nasal G-CSF, (B) GM-CSF, (C) IFN-γ, (D) IL-10, (E) IL-12p40, (F) IL-15, (G) IL-16, (H) IL-17A, (I) IL17E/IL-25, (J) IL-1RA, (K) IL-1 α, (L) IL-1β, (M) IL-2), (N) IL-21, (O) IL-22, (P) IL-27, (Q) IL-3, (R) IL-33, (S) IL-4), (T) IL-5, (U) IL-6, (V) IL-7, (W) IL-8, (X) IL-9, (Y) TARC, (Z) TNF-α, (Ä) TNF-β, (Ö) TSLP, and (Ü) VEGF in N-ERD patients (n=8) are displayed at baseline (V1) and after 24 weeks (V4) of dupilumab treatment. The line within each box represents the median, the bottom border represents the 25th percentile, and top border the 75th percentile of the data. Whiskers extend 1.5 times the interquartile range. The significance of changes between baseline and week 24 are indicated in individual graphs. ns: non-significant.

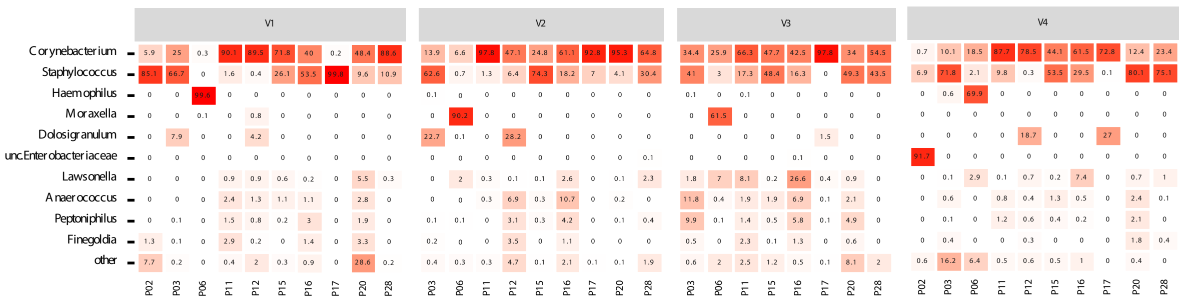

Supplementary Figure 4. Stacked bar charts representing microbial taxonomic profiles of individual N-ERD patients (n=10) during 24 weeks of dupilumab treatment. Samples are ordered by visits (panel V1-V4) and individual patients (x-axes). The fraction of a given bar marked by specific colour depicts the relative abundance of the indicated genus in the individual patient. In this figure patients with intermediate missing samples are included.
